# Detecting and quantifying causal associations in large nonlinear time series datasets

**DOI:** 10.1126/sciadv.aau4996

**Published:** 2019-11-27

**Authors:** Jakob Runge, Peer Nowack, Marlene Kretschmer, Seth Flaxman, Dino Sejdinovic

**Affiliations:** 1 German Aerospace Center, Institute of Data Science, 07745 Jena, Germany.; 2 Grantham Institute, Imperial College, London SW7 2AZ, UK.; 3 Department of Physics, Blackett Laboratory, Imperial College, London SW7 2AZ, UK.; 4 Data Science Institute, Imperial College, London SW7 2AZ, UK.; 5 Potsdam Institute for Climate Impact Research, 14473 Potsdam, Germany.; 6 Department of Mathematics, Imperial College, London SW7 2AZ, UK.; 7 The Alan Turing Institute for Data Science, London NW1 3DB, UK.; 8 Department of Statistics, University of Oxford, Oxford OX1 3LB, UK.

## Abstract

Identifying causal relationships and quantifying their strength from observational time series data are key problems in disciplines dealing with complex dynamical systems such as the Earth system or the human body. Data-driven causal inference in such systems is challenging since datasets are often high dimensional and nonlinear with limited sample sizes. Here, we introduce a novel method that flexibly combines linear or nonlinear conditional independence tests with a causal discovery algorithm to estimate causal networks from large-scale time series datasets. We validate the method on time series of well-understood physical mechanisms in the climate system and the human heart and using large-scale synthetic datasets mimicking the typical properties of real-world data. The experiments demonstrate that our method outperforms state-of-the-art techniques in detection power, which opens up entirely new possibilities to discover and quantify causal networks from time series across a range of research fields.

## Teaser

A novel causal discovery method for estimating nonlinear interdependency networks from large time series datasets.

## INTRODUCTION

How do major climate modes such as the El Niño Southern Oscillation (ENSO) influence remote regions via global teleconnections? How are physiological processes in the human body coupled? Also, through which pathways do different brain regions interact? Identifying causal association networks of multiple variables and quantifying causal strength are key challenges in the analysis of complex dynamical systems, especially since, here, interventional real experiments, the gold standard of scientific discovery, are often unethical or practically impossible. In climate research, model simulations can help to test causal mechanisms, but these are very expensive, time consuming, and represent only an approximation of the real-world physical processes (*1*). We here introduce an approach that learns causal association networks directly from time series data. These data-driven approaches have become increasingly attractive as recent decades have seen an explosion in data availability from simulations and real-world observations, for example, in Earth sciences (*2*). We therefore identify an urgent need for the development of novel causal discovery methods that can take advantage of this recent surge of big data, which, as we show here, has the potential to facilitate progress in many areas of sciences.

In a typical observational analysis scenario, for example, in climate science, a researcher has a hypothesis on the causal influence between two processes given observed time series data. The data may consist of different climatological variables (e.g., temperature and pressure) at one location, or of time series that represent regional averages of climatological variables, for example, commonly defined climate indices. For example, she may be interested in the influence of the regional ENSO index on an index characterizing the temperature variability over certain land areas of North America. Suppose the time series show a clear correlation, suggesting a relationship between the two processes. To exclude other possible hypotheses that may explain such a correlation, she will then include other relevant variables. In highly interconnected systems, there are typically many possible drivers she could test, quickly leading to high-dimensional causal discovery problems.

**Fig. 1:**
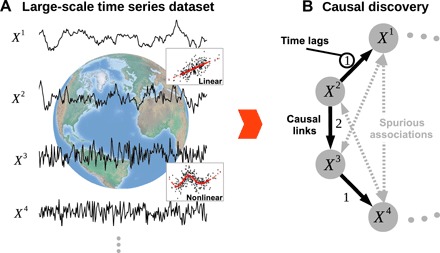
Causal discovery problem. Consider a large-scale time series dataset (**A**) from a complex system such as the Earth system of which we try to estimate the underlying causal dependencies (**B**), accounting for linear and nonlinear dependencies and including their time lags (link labels). Pairwise correlations yield spurious dependencies due to common drivers (e.g., *X*^1^ ← *X*^2^ → *X*^3^) or transitive indirect paths (e.g., *X*^2^ → *X*^3^ → *X*^4^). Causal discovery aims to unveil such spurious dependencies, leading to estimated causal networks that are, therefore, much sparser than correlation networks.

The goal in time series causal discovery from complex dynamical systems is to statistically reliably estimate causal links, including their time lags. Climatic teleconnections, for example, can take days to months. Two key challenges are the typically high dimensionality of these causal discovery problems and the often strong interdependencies. For instance, in a system comprising dozens to hundreds of variables (e.g., different regional climate indices), correlations will arise not only because of direct causal effects but also because of autocorrelation effects within each time series, indirect links, or common drivers (Fig. 1). Ideally, a causal discovery method detects as many true causal relationships as possible (high detection power) and controls the number of false positives (incorrect link detections). Causal discovery can help to better understand physical mechanisms, to build more parsimonious prediction models, and to more reliably estimate the strength of causal effects, which can be done in different frameworks, for example, the potential outcome (*3*) or graphical model frameworks (*4*, *5*). Put simply, causal discovery will be useful in situations where researchers wish to study complex dynamical systems in a way that goes beyond simple correlation analyses. Of course, any causal interpretation will rest on a number of assumptions (*4*, *5*) as we further discuss below.

A major current approach not only in Earth data analysis (*6*–*9*) but also in neuroscience (*10*, *11*) is to estimate time-lagged causal associations using autoregressive models in the framework of Granger causality (*12*, *13*). If implemented using standard regression techniques, then the high dimensionality of typical datasets leads to very low detection power (the “curse of dimensionality”) since sample sizes are often only on the order of a few hundred (e.g., for a monthly time resolution with 30 years of satellite data). This shortcoming leads to a dilemma that has limited applications of Granger causality mostly to bivariate analyses that cannot, however, account for indirect links or common drivers. Complementary to linear Granger causality, state-space methods (*14*, *15*) better address nonlinear state-dependent couplings, but these are also difficult to extend to high-dimensional scenarios.

There are methods that can cope with high dimensionality, such as regularized regression techniques (*16*–*18*), but mainly in the context of prediction and not causal discovery where assessing the significance of causal links is more important. An exception is Lasso regression (*17*), which also allows discovering active variables. Another approach with some recent applications in geosciences (*19*–*24*) is algorithms aimed specifically at causal discovery (*4*, *5*, *25*), which use iterative independence and conditional independence testing. However, both regularized regression (*26*) and recent implementations of causal discovery algorithms do not deal well with the strong interdependencies due to the spatiotemporal nature of the variables, as we show here. In particular, controlling false positives at a desired level is difficult for these methods and becomes even more challenging for nonlinear estimators. In summary, these problems lead to brittle estimates of causal networks and causal effects, and a more reliable methodology is required. In (*2*), the authors present an overview of the state of the art in causal inference methods and discuss related challenges with a focus on Earth sciences.

We present a causal network discovery method based on the graphical causal model framework (*5*) that scales well with large time series datasets featuring linear and nonlinear, time-delayed dependencies. Through analytical results, real-world applications, and extensive numerical experiments, we demonstrate that the proposed method has substantial advantages over the current state of the art in dealing with interdependent time series datasets on the order of dozens to hundreds of variables for sample sizes of a few hundred or more, yielding reliable false-positive control and higher detection power. We also find that more reliable causal network estimates yield more precise estimates of causal effects, bridging causal discovery with causal effect inference frameworks such as the potential outcome framework. Our approach enables causal analyses among more variables, opening up new opportunities to more credibly estimate causal networks and causal effects from time series in Earth system science, physiology, neuroscience, and other fields.

## CAUSAL DISCOVERY

### Motivating example from climate science

In the following, we illustrate the causal discovery problem on a well-known long-range teleconnection. We highlight two main factors that lead the common autoregressive Granger causal modeling approach to have low detection power: reduced effect size due to conditioning on irrelevant variables and high dimensionality.

Given a finite time series sample, every causal discovery method has to balance the trade-off between too many false positives (incorrect link detections) and too few true positives (correct link detections). A causality method ideally controls false positives at a predefined significance level (e.g., 5%) and maximizes detection power. The power of a method to detect a causal link depends on the available sample size, the significance level, the dimensionality of the problem (e.g., the number of coefficients in an autoregressive model), and the effect size, which, here, is the magnitude of the effect as measured by the test statistic (e.g., the partial correlation coefficient). Since the sample size and the significance level are usually fixed in the present context, a method’s power can only be improved by reducing the dimensionality or increasing the effect size (or both).

**Fig. 2:**
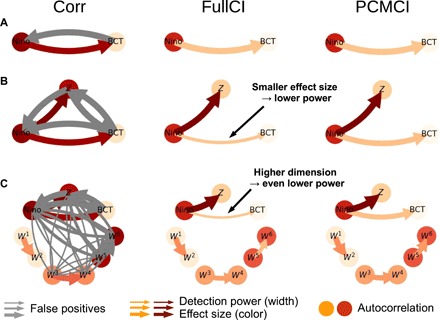
Motivational climate example. Correlation, FullCI partial correlation, and PCMCI partial correlation between the monthly climate index Nino (3.4 region) (*27*) and land air temperature over British Columbia (*28*) (**A**) for 1979–2017 (*T* = 468 months), as well as artificial variables [*Z* and *W^i^* in (**B** and **C**)]. Node colors depict autocorrelation strength, edge colors the partial correlation effect size, and edge widths the detection rate estimated from 500 realizations of the artificial variables *Z* and *W^i^* at a significance level of 5%. The maximum lag is τ_max_ = 6. Correlation does not allow for a causal interpretation, leading to spurious correlations (gray edges) (A). FullCI identifies the correct direction Nino→BCT but loses power because of smaller effect size (B) and higher dimensionality (C) if more variables are added. PCMCI avoids conditioning on irrelevant variables, leading to larger effect size, lower dimensionality, and, hence, higher detection power. See fig. S2 for more details.

Consider a typical causal discovery scenario in climate research (Fig. 2). We wish to test whether the observational data support the hypothesis that tropical Pacific surface temperatures, as represented by the monthly Nino 3.4 index (further referred to as Nino; see map and region in fig. S2) (*27*), causally affected extratropical land air temperatures (*28*) over British Columbia (BCT) for 1979–2017 (*T* = 468 months). We chose this example since it is well established and physically understood that atmospheric wave trains induced by increased sea surface temperatures over the tropical Pacific can affect North American temperatures but not the other way around (*9*, *29*–*31*). Thus, the ground truth here is Nino → BCT on the (intra)seasonal time scale, allowing us to validate causality methods.

We start with a time-lagged correlation analysis and find that the two variables are correlated in both directions, that is, for both positive and negative lags (Fig. 2A and see fig. S2 for lag functions), suggesting also an influence from BCT on Nino. The correlation Nino → BCT has an effect size of ≈ 0.3 (*P* < 10^−4^) at a lag of 2 months. In the networks in Fig. 2, the link colors denote effect sizes (gray links are spurious), and the node colors denote the autocorrelation strength.

Lagged correlation cannot be used to infer causal directionality and not even the correct time lag of a coupling (*20*). Hence, we now move to causal methods. To test Nino → BCT, the most straightforward approach then is to fit a linear autoregressive model of BCT on past lags of itself and Nino and test whether and which past coefficients of Nino are significantly different from zero. This is equivalent to a lag-specific version of Granger causality, but one can phrase this problem also more generally as testing for conditional independence between Nino_*t*−τ_ and BCT*_t_* conditional on (or controlling for) the common past $X_{t}^{-}=({\text{Nino}}_{t-1},{\text{BCT}}_{t-1},\ldots )$, denoted ${\text{Nino}}_{t-\tau }⫫{\text{BCT}}_{t}|X_{t}^{-}\setminus \{{\text{Nino}}_{t-\tau }\}$. For estimating conditional independencies (see below), the time index *t* runs through the samples up to the time series length *T*. $X_{t}^{-}$ is, in practice, truncated at a maximum time lag τ_max_, which depends on the application and can be chosen according to the maximum causal time lag expected in the complex system or based on the largest lag with significant correlation. We call this general approach full conditional independence testing (FullCI; see table S3 for an overview of methods considered in this paper) and illustrate it in a linear partial correlation implementation for this example, that is, we test $\rho ({\text{Nino}}_{t-\tau },{\text{BCT}}_{t}|X_{t}^{-}\setminus \{{\text{Nino}}_{t-\tau }\})\neq 0$ for different lags τ, which is the effect size for FullCI.

Using a maximum time lag τ_max_ = 6 months, we find a significant FullCI partial correlation for Nino → BCT at lag 2 of 0.1 (*P* = 0.037) (Fig. 2A) and no significant dependency in the other direction. That is, the effect size of FullCI is strongly reduced compared to the correlation (≈ 0.3) when taking into account the past. However, as mentioned before, such a bivariate analysis can usually not be interpreted causally, because other processes might explain the relationship. To further test our hypothesis, we include another variable *Z* that may explain the dependency between Nino and BCT (Fig. 2B). Here, we generate *Z* artificially for illustration purposes and define$Z_{t}=2\cdot {\text{Nino}}_{t-1}+\eta_{t}^{Z}$ for independent standard normal noise $\eta_{t}^{Z}$. Thus, Nino drives *Z* with lag 1, but *Z* has no causal effect on BCT, which we assume a priori unknown. Here, we simulated different realizations of *Z* to measure detection power and false-positive rates. We find that the correlation would be even more misguiding a causal interpretation since we observe spurious links between all variables (Fig. 2B). The FullCI partial correlation, with $X_{t}^{-}$ including the past of all three processes and not just BCT and Nino, now has an effect size of 0.09 for Nino_*t*−2_ → BCT*_t_* compared to 0.1 in the bivariate case. At a 5% significant level, this link is only detected in 53% of the realizations (true-positive rate, arrow width in Fig. 2).

What happened here? As mentioned above, detection power depends on dimensionality and effect size. Conditioning on the past of variable *Z* slightly increases the dimensionality of the conditional independence test, but this only partly explains the low detection power. If *Z* is constructed in such a way that it is independent of Nino, then the FullCI partial correlation is 0.1 again, as in the bivariate case, and the true-positive rate is 85%. The more important factor is that, since Nino drives *Z*, *Z* contains information about Nino, and because *Z* is part of the conditioning set $X_{t}^{-}$, it now “explains away” some part of the partial correlation $\rho ({\text{Nino}}_{t-2},{\text{BCT}}_{t}|X_{t}^{-}\setminus \{{\text{Nino}}_{t-2}\})$, thereby leading to an effect size that is just 0.01 smaller, which already strongly reduces the detection rate.

Suppose we got one of the realizations of *Z* for which the link Nino_*t*−2_ → BCT*_t_* is still significant. To further illustrate the effect of high dimensionality on detection power, we now include six more variables *W^i^* (*i* = 1, …, 6), which are all independent of Nino, BCT, and *Z* but coupled between each other in the following way (Fig. 2C): $W_{t}^{i}=a^{i}W_{t-1}^{i}+cW_{t-2}^{i-1}+\eta_{t}^{i}$ for *i* = 2, 4, 6 and $W_{t}^{i}=a^{i}W_{t-1}^{i}+\eta_{t}^{i}$ for *i* = 1, 3, 5, all with the same coupling coefficient *c* = 0.15 and *a*^1,2^ = 0.1, *a*^3,4^ = 0.5, and *a*^5,6^ = 0.9. Now, the FullCI effect size for Nino_*t*−2_ → BCT*_t_* is still 0.09, but the detection power is even lower than before and decreases from 53% to only 40% because of the higher dimensionality. Thus, the true causal link Nino_*t*−2_ → BCT*_t_* is likely to be overlooked.

Effect size is also affected by autocorrelation effects of the included variables: The coupled variable pairs $W_{t-2}^{i-1}\to W_{t}^{i}$ (*i* = 2, 4, 6) differ in their autocorrelation (as visualized by their node color in Fig. 2C) and, although the coupling coefficient *c* is the same for each pair, their FullCI partial correlations are 0.15, 0.13, and 0.11 (from lower to higher autocorrelation). Similar to the above case, conditioning on past lags, here $W_{t-1}^{i}$ (*i* = 1, 3, 5), explains away information, leading to a smaller effect size and lower power the higher their autocorrelation is. Conversely, we here observe more spurious correlations for higher autocorrelations (Fig. 2C, left).

This example illustrates a ubiquitous dilemma of causal discovery in many fields: To strengthen the credibility of causal interpretations, we need to include more variables that might explain a spurious relationship, but these lead to lower power to detect true causal links due to higher dimensionality and possibly lower effect size. Low detection power also implies that causal effect estimates become less reliable as we show in Results. Ideally, we want to condition only on the few relevant variables that actually explain a relationship.

### Causal network discovery with PCMCI

The previous example has shown the need for an automated procedure that better identifies the typically few relevant variables to condition on. We now introduce such a causal discovery method that helps to overcome the above dilemma and more reliably estimates causal networks from time series data.

**Fig. 3:**
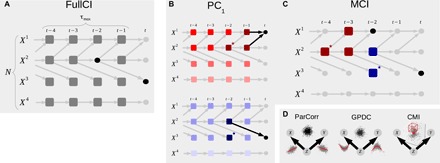
Proposed causal discovery method. (**A**) Time series graph (*32*–*34*) representing the time-lagged causal dependency structure underlying the data. FullCI tests the presence of a causal link by $X_{t-\tau }^{i}⫫X_{t}^{j}|X_{t}^{-}\setminus \{X_{t-\tau }^{i}\}$, where ⫫ denotes (conditional) independence and $X_{t}^{-}\setminus \{X_{t-\tau }^{i}\}$ the past of all *N* variables up to a maximum time lag τ_max_ excluding $X_{t-\tau }^{i}$ (gray boxes). (**B**) Illustration of PC_1_ condition selection algorithm for the variables *X*^1^ (top) and *X*^3^ (bottom): The algorithm starts by initializing the preliminary parents $\hat{P}(X_{t}^{j})=X_{t}^{-}$. In the first iteration (*p* = 0), variables without even an unconditional dependency (e.g., uncorrelated) are removed from $\hat{P}(X_{t}^{j})$ (lightest shade of red and blue, respectively). In the second iteration (*p* = 1), variables that become independent conditional on the driver in $\hat{P}(X_{t}^{j})$ with largest dependency in the previous iteration are removed. In the third iteration (*p* = 2), variables are removed that are independent conditionally on the two strongest drivers and so on until there are no more conditions to test in $\hat{P}(X_{t}^{j})$. In this way, PC_1_ adaptively converges to typically only few relevant conditions (dark red/blue) that include the causal parents P with high probability and potentially some false positives (marked with a star). (**C**) These low-dimensional conditions are then used in the MCI conditional independence test: For testing $X_{t-2}^{1}\to X_{t}^{3}$, the conditions $\hat{P}(X_{t}^{3})$ (blue boxes) are sufficient to establish conditional independence, while the additional conditions on the parents $\hat{P}(X_{t-2}^{1})$ (red boxes) account for autocorrelation and make MCI an estimator of causal strength. (**D**) Both the PC_1_ and the MCI stage can be flexibly combined with linear (ParCorr) or nonlinear (GPDC and CMI) independence tests (see section S4 and table S1). ParCorr assumes linear additive noise models and GPDC only additivity. The gray scatter plots illustrate regressions of *X*, *Y* on *Z* and the black scatter plots the residuals. The red cubes in CMI illustrate the data-adaptive model–free *k*-nearest neighbor test (*44*), which does not require additivity.

Graphical models (*4*, *5*) are a convenient way to represent causal interdependencies of a system. While the networks depicted in Fig. 1B and Fig. 2 are easier to visualize, they do not fully represent the spatiotemporal dependency structure underlying complex dynamical systems. Time series graphs (*32*–*34*) provide a more comprehensive view (see Fig. 3 and section S1 for more details). Consider an underlying time-dependent system $X_{t}=(X_{t}^{1},\ldots ,X_{t}^{N})$ with

$$X_{t}^{j}=f_{j}(P(X_{t}^{j}),\eta_{t}^{j})$$ (1)

where *f_j_* is some potentially nonlinear functional dependency and $\eta_{t}^{j}$ represents mutually independent dynamical noise. The nodes in a time series graph represent the variables $X_{t}^{j}$ at different lag times, and $P(X_{t}^{j})\subset X_{t}^{-}=(X_{t-1},X_{t-2},\ldots )$ denotes the causal parents of variable $X_{t}^{j}$ (Fig. 3B, nodes with black arrows) among the past of all *N* variables. A causal link $X_{t-\tau }^{i}\to X_{t}^{j}$ exists if $X_{t-\tau }^{i}\in P(X_{t}^{j})$. Another way to define links is that $X_{t-\tau }^{i}$ is not conditionally independent of $X_{t}^{j}$ given the past of all variables, defined by Xt−τi⫫Xtj∣Xt−∖{Xt−τi}, with ⫫ denoting the absence of a (conditional) independence (*34*). The goal in causal discovery is then to estimate the causal parents from time series data. FullCI directly tests the link-defining conditional independence, but recall that in Fig. 3A, the high dimensionality of including *N*τ_max_ − 1 conditions on the one hand, and the reduced effect size due to conditioning on $X_{t-1}^{1}$ and $X_{t-1}^{2}$ (similar to the example in Fig. 2), on the other, leads to a potentially drastically reduced detection power of FullCI.

Causal discovery theory (*4*, *5*) tells us that the parents $P(X_{t}^{j})$ of a variable $X_{t}^{j}$ are a sufficient conditioning set that allows establishing conditional independence [causal Markov property (*5*)]. Thus, in contrast to conditioning on the whole past of all processes as in FullCI, conditioning only on a set that at least includes the parents of a variable $X_{t}^{j}$ suffices to identify spurious links. Markov discovery algorithms (*5*, *35*) such as the PC algorithm (named after its inventors) (*25*) allow us to detect these parents and can be flexibly implemented with different kinds of conditional independence tests that can accommodate nonlinear functional dependencies and variables that are discrete or continuous. These properties allow for greater flexibility than attempting to directly fit the possibly very complex functional dependencies in Eq. 1. However, as shown in our numerical experiments, the PC algorithm should not be directly used for the time series case.

Our proposed approach is also based on the conditional independence framework (*5*) and adapts it to the highly interdependent time series case. The method, which we name PCMCI, consists of two stages: (i) PC_1_ condition selection (Fig. 3B and algorithm S1) to identify relevant conditions $\hat{P}(X_{t}^{j})$ for all time series variables $X_{t}^{j}\in \{X_{t}^{1},\ldots ,X_{t}^{N}\}$ and (ii) the momentary conditional independence (MCI) test (Fig. 3C and algorithm S2) to test whether $X_{t-\tau }^{i}\to X_{t}^{j}$ with

MCI:Xt−τi⫫Xtj∣Pˆ(Xtj)∖{Xt−τi},Pˆ(Xt−τi) (2)

Thus, MCI conditions on both the parents of $X_{t}^{j}$ and the time-shifted parents of $X_{t-\tau }^{i}$. The two stages (i) and (ii) serve the following purposes: PC_1_ is a Markov set discovery algorithm based on the PC-stable algorithm (*36*) that removes irrelevant conditions for each of the *N* variables by iterative independence testing (illustrated by shades of red and blue in Fig. 3B). A liberal significance level α_PC_ in the tests lets PC_1_ adaptively converge to typically only few relevant conditions (dark red/blue) that include the causal parents P in Eq. 1 with high probability but might also include some false positives (marked with a star in Fig. 3B). The MCI test (Fig. 3C) then addresses false-positive control for the highly interdependent time series case.

More precisely, in the PC_1_ algorithm, we start for every variable $X_{t}^{j}$ by initializing the preliminary parents $\hat{P}(X_{t}^{j})=(X_{t-1},X_{t-2},\ldots ,X_{t-\tau_{\text{max}}})$. In the first iteration (*p* = 0), we conduct unconditional independence tests and remove $X_{t-\tau }^{i}$ from $\hat{P}(X_{t}^{j})$ if the null hypothesis $X_{t-\tau }^{i}⫫X_{t}^{j}$ cannot be rejected at a significance level α_PC_. In Fig. 3B, for the parents of $X_{t}^{1}$, this would likely be the case for the lagged variables $X_{t-\tau }^{4}$ (light shades of red). In each next iteration (*p* → *p* + 1), we first sort the preliminary parents by their (absolute) test statistic value and then conduct conditional independence tests $X_{t-\tau }^{i}⫫X_{t}^{j}|S$, where S are the strongest *p* parents in $\hat{P}(X_{t}^{j})\setminus \{X_{t-\tau }^{i}\}$. After each iteration, independent parents are removed from $\hat{P}(X_{t}^{j})$, and the algorithm converges if no more conditions can be tested (see details in Materials and Methods). In Fig. 3B, for $X_{t}^{3}$ (blue shades), the algorithm converges already after *p* = 1-dimensional conditions have been tested. Since these tests are all very low dimensional compared to FullCI (or Granger causality), they have higher detection power.

In the second stage, the MCI test (Fig. 3C) uses the estimated conditions as follows. For testing $X_{t-2}^{1}\to X_{t}^{3}$, the conditions $\hat{P}(X_{t}^{3})$ (blue boxes in Fig. 3C) are sufficient to establish conditional independence (Markov property), that is, to identify indirect and common cause links. The additional condition on the lagged parents $\hat{P}(X_{t-2}^{1})$ (red boxes) accounts for autocorrelation, leading to correctly controlled false-positive rates at the expected level as further discussed below in our theoretical results. The significance of each link can be assessed based on the *P* values of the MCI test. These can, subsequently, also be adjusted according to procedures such as false discovery rate control (*37*). The main free parameter of PCMCI is the significance level α_PC_ in PC_1_, which should be regarded as a hyperparameter and can be chosen on the basis of model selection criteria such as the Akaike information criterion (AIC) or cross-validation. Further technical details can be found in Materials and Methods.

Our method addresses the problem of detecting the topological structure of causal networks, that is, the existence or absence of links (at different time lags). A follow-up question is to quantify causal effects, that is, the strength of causal links, which can be done not only in the framework of graphical causal models (*4*, *5*, *38*, *39*) but also using other frameworks such as structural causal modeling (*4*) or potential outcomes (*3*, *40*). The three frameworks are equivalent (*41*) but differ in their notation and how assumptions are formulated. In the “Estimating causal effects” section, we will demonstrate how our causal discovery method can be used to more reliably estimate causal effects in high-dimensional settings.

### Linear and nonlinear implementations

Both the PC_1_ and the MCI stage can be flexibly combined with any kind of conditional independence test. Here, we present results for linear partial correlation (ParCorr) and two types of nonlinear (GPDC and CMI) independence tests (Fig. 3D). GPDC is based on Gaussian process regression (*42*) and a distance correlation (*43*) test on the residuals, which is suitable for a large class of nonlinear dependencies with additive noise. CMI is a fully nonparametric test based on a *k*-nearest neighbor estimator of conditional mutual information that accommodates almost any type of dependency (*44*). The drawback of greater generality for GPDC or CMI, however, is lower power for linear relationships in the presence of small sample sizes. These conditional independence tests are further discussed in section S4 and table S1.

### Assumptions of causal discovery from observational data

Our method and notation follows the graphical causal model framework (*4*, *5*). For a causal interpretation based solely on observational data, this framework rests on the standard assumptions (*5*) of Causal Sufficiency (or Unconfoundedness), implying that all common drivers are among the observed variables, the Causal Markov Condition, implying that $X_{t}^{j}$ is independent of $X_{t}^{-}\setminus P(X_{t}^{j})$ given its parents $P(X_{t}^{j})$, and the Faithfulness assumption, which requires that all observed conditional independencies arise from the causal graphical structure. For the present time series case, we assume no contemporaneous causal effects and, since typically only a single realization is available, we also assume stationarity. Another option would be to use independent ensembles of realizations of lagged processes. We elaborate on these assumptions in Discussion. See (*2*, *34*) for an overview of causal discovery on time series.

## RESULTS

### Theoretical properties of PCMCI

We here briefly discuss several advantageous properties of PCMCI, in particular, its computational complexity, consistency, generally larger effect size than FullCI, and interpretability as causal strength, as explained in more detail in section S5.

In the condition selection stage, PCMCI efficiently exploits sparsity in the causal network and has a complexity in the number of variables *N* and maximum time lag τ_max_ that is polynomial. In the numerical experiments, we show that runtimes are comparable or faster than state-of-the-art methods. Consistency implies that PCMCI provably estimates the true causal graph in the limit of infinite sample size under the standard assumptions of causal discovery (*5*, *34*) and also in the nonlinear case, provided that the correct class of conditional independence tests is used. In section S5.3, we also elaborate on why MCI, empirically, well controls false positives even for highly autocorrelated variables, which is due to the conditioning on the parents $\hat{P}(X_{t-\tau }^{i})$ of the lagged variable. Theoretical results for finite samples would require strong assumptions (*45*, *46*) or are mostly impossible, especially for nonlinear associations. Because of the condition selection stage, MCI typically has a much lower conditioning dimensionality than FullCI. Further, avoiding conditioning on irrelevant variables also can be shown to always yield a greater (or equal) effect size than FullCI. Irrelevant variables are not explanatory for causal relationships, and they may also lead to smaller effect sizes if they are caused by the considered driver variable. Both of these factors lead to typically much higher detection power than FullCI (or Granger causality) for small and large numbers of variables as further discussed in section S5.4. Last, while detecting the causal network structure is the main goal of PCMCI, the MCI test statistic also yields a well-interpretable notion of a normalized causal strength, as further discussed in section S5.5 and (*38*, *39*). Thus, the value of the MCI statistic (e.g., partial correlation or CMI) allows us to rank causal links in large-scale studies in a meaningful way.

### Real-world applications

To validate causal discovery methods, we ideally would have real-world datasets with known underlying ground truth of causal dependencies. Such datasets are rare especially for the causal interdependencies of large numbers of variables. Here, we analyze small-scale climate and cardiovascular examples where the underlying physical mechanisms are well understood. In the next section, we also validate the method on large-scale synthetic datasets that mimic the properties of real-world data. In (*2*), the causality benchmark platform www.causeme.net is introduced, which facilitates method evaluation on a growing body of synthetic and real-world datasets.

Returning to the motivating climate example including synthetic variables (Fig. 2, right), PCMCI efficiently estimates the true causal relationships with high power in all three cases, in contrast to FullCI. The condition selection algorithm PC_1_ identifies only the relevant conditions and finds, in particular, that *Z* is not a parent of BCT. The MCI conditional independence test for the link Nino_*t*−2_ → BCT*_t_* then has the same partial correlation effect size ≈0.10 (*P* = 0.036 in case A) in all three cases (Fig. 2, A to C). The detection power is >80% even for the high-dimensional case in Fig. 2C. Furthermore, PCMCI correctly estimates the causal effect strength ≈ 0.14 among the links $W_{t-2}^{i-1}\to W_{t}^{i}$ (*i* = 2, 4, 6), resulting in similar detection power irrespective of different autocorrelations in different *W^i^* time series.

**Fig. 4:**
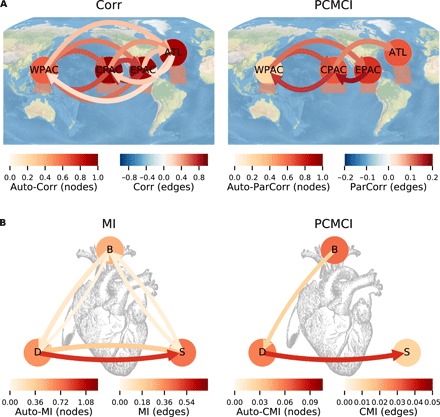
Real-world applications. (**A**) Tropical climate example of dependencies between monthly surface pressure anomalies for 1948–2012 (*T* = 780 months) in the West Pacific (WPAC; regions depicted as shaded boxes below nodes), as well as surface air temperature anomalies in the Central (CPAC) and East Pacific (EPAC), and tropical Atlantic (ATL) (*65*). The left panel shows correlation (Corr), and the right panel shows PCMCI in the ParCorr implementation with τ_max_ = 7 months to also capture long time lags. Significance was assessed at a strict 1% level. (**B**) Cardiovascular example of links between heart rate (B) and diastolic (D) and systolic (S) blood pressure (*T* = 600) of 13 healthy pregnant women. The left panel shows MI, and the right panel shows PCMCI in the CMI implementation with τ_max_ = 5 heart beats and default parameters *k*_CMI_ = 60 and *k*_perm_ = 5 (see table S1). The graphs are obtained by analyzing PCMCI separately for the 13 datasets and showing only links that are significant at the 1% level in at least 80% of the subjects. In all panels, node colors depict autodependency strength and edge colors the cross-link strength at the lag with maximum absolute value. See lag functions in fig. S3 and Materials and Methods for more details on the datasets. Note the different scales in colorbars.

In Fig. 4A, we show that PCMCI can reconstruct the Walker circulation (*47*) in the tropical Pacific including the link to the Atlantic, where the underlying physical mechanism is theoretically well understood and has been validated with detailed physical simulation experiments (*48*): Warm surface air temperature anomalies in the East Pacific (EPAC) are carried westward by trade winds across the Central Pacific (CPAC). Then, the moist air rises over the West Pacific (WPAC), and the circulation is closed by the cool and dry air sinking eastward across the entire tropical Pacific. Furthermore, the CPAC region links temperature anomalies to the tropical Atlantic (ATL) via an atmospheric bridge (*49*). Pure lagged correlation analysis results in a completely connected graph with significant correlations at almost all time lags (see lag functions in fig. S3), while PCMCI with the linear ParCorr conditional independence test better identifies the Walker circulation and Atlantic teleconnection. In particular, the link from EPAC to WPAC is correctly identified as indirectly mediated through CPAC.

From the Earth system, we turn to the human heart in Fig. 4B. We investigate time series of heart rate (B), as well as diastolic (D) and systolic (S) blood pressure of pregnant healthy women (*50*, *51*). It is well understood that the heart rate influences the cardiac stroke volume, which, in turn, drives diastolic blood pressure (Starling’s law). Furthermore, the mechanism by which diastolic blood pressure drives systolic blood pressure is the effect of the stroke volume, the corresponding pulse pressure, and the total peripheral resistance (*52*). Here, we cannot assume linear interdependencies and, thus, use the information-theoretic CMI implementation of PCMCI. With mutual information (MI), we obtain only a fully connected graph, while the physiologically plausible causal chain *B* → *D* → *S* is correctly reconstructed with PCMCI.

These examples for a relatively small number of variables show how causal discovery with PCMCI helps to identify physical mechanisms from time series. As further detailed in Discussion, since these analyses typically cannot assume that no unobserved common drivers exist, care should be taken with a causal interpretation of direct links. On the other hand, the absence of direct links can indeed be interpreted as the absence of direct causal associations under weaker assumptions, such as in the case of those from the EPAC to the WPAC and from heart rate to systolic blood pressure.

### Model setup for high-dimensional synthetic data experiments

**Fig. 5:**
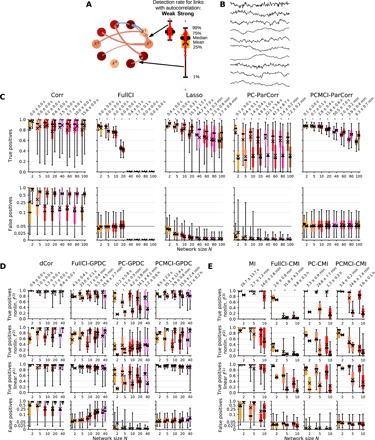
Numerical experiments for causal network estimation. (**A**) The full model setup is described in section S6 and table S2. In total, 20 coupling topologies for each network size *N* were randomly created, where all cross-link coefficients are fixed while the variables have different autocorrelations. An example network for *N* = 10 with node colors denoting autocorrelation strength is shown, and the arrow colors denote the (positive or negative) coefficient strength. The arrow width illustrates the detection rate of a particular method. As indicated here, the boxplots in the figures below show the distribution of detection rates across individual links with the left (right) boxplot depicting links between weakly (strongly) autocorrelated variable pairs, defined by the average autocorrelation of both variables being smaller or larger than 0.7. (**B**) Example time series realization of a model depicting partially highly autocorrelated variables. Each method’s performance was assessed on 100 such realizations for each random network model. (**C**) Performance of different methods for models with linear relationships with time series length *T* = 150. Table S3 provides implementation details. The bottom row shows boxplot pairs (for weakly and strongly autocorrelated variables) of the distributions of false positives, and the top row shows the distributions of true positives for different network sizes *N* along the *x* axis in each plot. Average runtime and its SD are given on top. (**D**) Numerical experiments for nonlinear GPDC implementation with *T* = 250, where dCor denotes distance correlation. (**E**) Results for CMI implementation with *T* = 500, where MI denotes mutual information. In both panels, we differentiate between linear and two types of nonlinear links (top three rows). See table S2 for model setups and section S4 and table S1 for a description of the nonlinear conditional independence tests.

Following our illustrative examples, we evaluate and compare the performance of PCMCI together with other common causal methods more systematically in numerical experiments that mimic the properties of real-world data. Here, we model six of the major challenges of time series from complex systems: high dimensionality, time-lagged causal dependencies, autocorrelation, strong nonlinearity, observational noise, and nonstationarity (*2*). Figure 5A gives an example model for *N* = 10 variables, where the edge colors denote the (positive or negative) coefficient corresponding to causal links and the node color depicts the autocorrelation strength. Figure 5B shows a time series realization illustrating some strongly autocorrelated variables. We create a number of models with different random network topologies of *N* time series variables with each network having *L* = *N* linear or nonlinear causal dependencies (except for the bivariate case *N* = 2 with *L* = 1). From each of these models, we generate 100 time series datasets (each of length *T*) to assess true- and false-positive rates of individual causal links in a model with the different causal methods. As illustrated in Fig. 5A, the boxplots in the following figures show the distribution of these individual link false- and true-positive rates across the large variety of random networks, for each network size *N* differentiated between weakly and strongly autocorrelated pairs of variables in the left and right boxplots, respectively (defined by the average autocorrelation of both variables being smaller or larger than 0.7). We depict only results for cross-links here, not for auto-links within a variable. The full model setup is detailed in section S6, table S2 lists the experimental setups, and section S2 and table S3 give details on the compared methods.

### High dimensionality with linear relationships

In Fig. 5C, we first investigate the performance of linear causal discovery methods on numerical experiments with linear causal links; nonlinear models are shown in Fig. 5 (D and E). The setup has a sample length of *T* = 150 observations and *N* = 2, …, 100 variables. All cross-links have the same absolute coupling coefficient value (but with different signs) and, hence, the same causal strength. Next to correlation (Corr) and FullCI (similar to Granger causality, here implemented with an efficient vector-autoregressive model estimator), we compare PCMCI with the original PC algorithm as a standalone method and Lasso regression (pseudo-code given in algorithm S3) as the most widely used representative of regularized high-dimensional regression techniques that can be used for causal variable selection. Table S3 gives an overview of the compared methods, and implementation details for alternative methods are given in section S2. The maximum time lag is τ_max_ = 5, and the significance level is 5% for all methods.

**Fig. 6:**
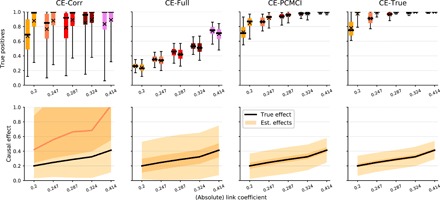
Numerical experiments for causal effect estimation. Shown is detection power (top row) and causal effect size (bottom row) as given by univariate linear regression (CE-Corr), multivariate regression on the whole past of the multivariate process (CE-Full), and multivariate regression on just the parents obtained with PCMCI (CE-PCMCI) for different link coefficient strengths *c* along the *x* axis in each plot. The last column denotes the regression on the true parents (CE-True). In the bottom row, the orange shades give the 1, 25, 75, and 99% quantiles and the median of the respective causal effects of all links (mean over 100 realizations for each link), and the black line denotes the true causal effect strength |*c*|, which is the same for all links in a model.

Correlation is obviously inadequate for causal discovery with very high false-positive rates (first column in Fig. 5C). However, even detection rates for true links vary widely with some links with under 20% true positives despite the equal coefficient strength for all causal links. This counterintuitive result is further investigated in Fig. 6. In contrast, all causal methods control false positives well around or below the chosen 5% significance level with Lasso and the PC algorithm overcontrolling at lower than expected rates. An exception here are some highly autocorrelated links that are not correctly controlled with the PC algorithm (whiskers extending to 25% false positives in Fig. 5C) since it does not appropriately deal with the time series case.

While FullCI has a detection power of around 80% for *N* = 5, this rate drops to 40% for *N* = 20, and FullCI cannot be applied anymore for larger *N* when the dimensionality is larger than the sample size (*N*τ_max_ > *T* = 150). However, for *N* = 5 as well, some links between strongly autocorrelated variables have a detection rate of just 60%. Lasso has higher detection power than FullCI on average and can be applied also to the high-dimensional *N*τ_max_ > *T* case. The PC algorithm displays not much difference in detection power between *N* = 5 and *N* = 100, but the rates are lower than for Lasso on average, and higher autocorrelation also here has a detrimental effect. Note that it is difficult to compare power levels here since Lasso and PC cannot be easily calibrated to have an expected significance level.

PCMCI robustly shows high detection power even for network sizes with dimensions exceeding the sample size and displays almost the same power for links with the same causal effect, regardless of whether autocorrelations are weak or strong, up to *N* = 20.

An analysis of covariance (ANCOVA) was performed to more quantitatively investigate the dependence of detection power and false positives on the number of variables *N* and the sample size *T* (see section S6, fig. S17, and tables S9 to S12). It reveals that FullCI has indeed the strongest decrease, and PCMCI and Lasso have similar decreases in detection power for higher numbers of variables *N*, with PCMCI slightly outperforming Lasso for smaller *N*. Similarly, PCMCI benefits slightly more than Lasso from larger sample sizes. ANCOVA interaction effects regarding detection rates between different levels of *N* and *T* are present for both FullCI and PCMCI where power decreases less strongly with *N* for larger sample sizes. Lasso and the PC algorithm have no interaction effects in detection power. For false positives, we did not observe a relevant dependence of FullCI and PCMCI on either *N* or *T*, while PC and Lasso have decreasing levels for larger *N* as noted above and not much change for different *T*.

Runtime depends on implementation details, but all methods are in the same order of magnitude, except for FullCI, which was estimated with an efficient solver in this linear case. For nonlinear implementations, it can be much slower than PCMCI or PC (see the next section). PCMCI efficiently exploits sparsity. Our numerical experiments show that for smaller networks, PCMCI is faster than Lasso and vice versa for larger networks, but both have similar runtimes for larger *T* (fig. S16 and tables S4 to S7). Most of the time of PCMCI is spent on the condition selection stage, mainly because of the hyperparameter optimization of α_PC_ via AIC in the implementation shown. Fixing α_PC_ is much faster and still gives good results (figs. S4 and S16) but may not control false positives as well. The runtime of the standalone PC algorithm strongly depends on the number of conditioning sets tested. In theory, all combinations of conditioning sets are tested, which results, next to low power, in a slow and highly varying runtime especially for nonlinear implementations (fig. S16), but here, we limited the number of combinations (see section S2). In these linear numerical experiments, PC is still faster than PCMCI since no hyperparameter optimization was conducted, while for nonlinear implementations, it is often slower (see the next section).

In summary, our key result here is that PCMCI has high power even for network sizes with dimensions, given by *N*τ_max_, exceeding the sample size. Average power levels (marked by “x” in Fig. 5C) are higher than FullCI (or Granger causality) and PC for all considered network sizes. PCMCI has similar or larger average power levels compared to Lasso, but an important difference is the worst-case performance: Even for small networks (*N* = 10), a significant part of the links is constantly overlooked with Lasso, while for PCMCI, 99% of the links have a detection power greater than 70%.

### High dimensionality with nonlinear relationships

Figure 5 (D and E) displays results for nonlinear models where we differentiate between linear and two types of nonlinear links [upper three rows, *T* = 250 (D) and *T* = 500 (E)]. In essence, here, we find that PCMCI’s ability to avoid high dimensionality is even more crucial not only for detection power but also to correctly control false positives.

In Fig. 5D, FullCI, PC, and PCMCI are all implemented with the GPDC conditional independence test, and dCor denotes the distance correlation as the nonlinear analog to correlation (see section S4 and table S1). Distance correlation alone detects nonlinear links but does not account for indirect or common driver effects, leading to high false positives, especially for strong autocorrelation. FullCI here works well only up to *N* = 5 but cannot control false positives anymore for *N* ≥ 10 since the GPDC test does not work well in these high dimensions [see also analysis of variance (ANOVA) analyses in tables S6 and S7]. PC overcontrols false positives again (except for strong autocorrelation) and has the lowest power levels among all methods. PCMCI has the highest power levels, which only slightly decrease for larger networks. Here, we find for nonlinear dependencies that weakly and strongly autocorrelated links also result in different power levels, unlike for linear links (see section S5.5). False positives are mostly controlled correctly, but there is a slight inflation of false positives for larger networks, again, because even with condition selection, the dimensionality increases for larger networks, and GPDC does not work well in high dimensions. For GPDC, runtime for PC and PCMCI is larger than for FullCI.

Figure 5E depicts results for the fully nonparametric implementation with CMI. Then, FullCI has the slowest runtime and almost no power, especially for nonlinear links, while PCMCI correctly controls false positives and has, on average, higher power than PC. Nevertheless, strong nonlinearities are difficult to detect for the relatively high-dimensional cases studied here and with *T* = 500 samples.

### Further experiments

In the Supplementary Materials, we investigate some further methodological variants (see sections S2 and S3) and show that our results are robust also for larger sample sizes (figs. S4, S5, S9, S11, and S13 and tables S4, S6, and S7) and higher network coupling densities (figs. S6 and S7, and table S5). Further, we investigate the effect of violations of underlying theoretical assumptions, in particular, observational noise, nonstationarity, and nonfaithful processes, the latter represented by strongly nonlinear, purely deterministic dependencies.

All methods display a similar sensitivity to observational noise with PCMCI and Lasso being slightly more affected than FullCI according to an ANOVA analysis (fig. S14 and table S8) with levels up to 25% of the dynamical noise SD having only minor effects. For levels of the same order as the dynamical noise, we observe a stronger degradation with also the false positives not being correctly controlled, except for FullCI, since common drivers are no longer well detected anymore. See (*34*) for a discussion on observational noise.

In fig. S15 and table S8, we investigate the effect of a nonstationary trend, here modeled by an added sinusoidal signal with different amplitudes. Lasso is especially sensitive here and has both a lower detection power and an inflated rate of false positives, while PCMCI is robust even for high trend amplitudes.

Last, in fig. S18, we study the effect of strong common drivers for low-dimensional deterministic chaotic systems. Purely deterministic systems may violate Faithfulness since they can render variables connected by a true causal link as independent conditionally on another variable that fully determines either of them. A nonlinear dynamics-inspired method (*15*, *53*) that is adapted to these systems is convergent cross mapping (CCM; see section S2.4) (*14*), which we here compare with PCMCI in the CMI implementation. We find that for purely deterministic dependencies (fig. S18, A and C), CCM has higher detection rates that only degrade for very strong coupling strengths. PCMCI is not well suited for highly deterministic systems since it strongly conditions on the past of the driver system and, hence, removes most of the information that could be measured in the response system. If we study the same system driven by dynamical noise, the PCMCI detection rates strongly increase and outperform CCM (fig. S18, B and D). An advantage of PCMCI here is that it better controls false positives than CCM, which can have very high and uncontrolled false-positive levels. Note that, to test a causal link *X* → *Y*, CCM only uses the time series of *X* and *Y* with the underlying assumption that the dynamics of a common driver can be reconstructed using delay embedding. See (*34*) for a more in-depth study.

### Estimating causal effects

In this section, we show how our proposed method can be used to more precisely quantify causal effects assuming linear dependencies. In Discussion, we elaborate on different ways to more generally quantify causal strength. But first, we briefly discuss the different, but equivalent (*41*), theoretical frameworks to causal effect inference. In the graphical causal model framework (*4*), a causal effect of a link $X_{t-\tau }^{i}\to X_{t}^{j}$ is based on the interventional distribution $P(X_{t}^{j}|\text{do}(X_{t-\tau }^{i}=x))$, which is the probability distribution of $X_{t}^{j}$ at time *t* if $X_{t-\tau }^{i}$ was forced exogenously to have a value *x*. Causal effects can also be studied using the framework of potential outcomes (*3*), which mainly targets the social sciences and medicine. In this framework, causal effects can be defined as the difference between two potential outcomes, one where a subject *u* has received a treatment, denoted *Y_u_*(*X* = 1), and one where no treatment was given, denoted *Y_u_*(*X* = 0). In observational causal inference, *Y_u_*(*X* = 1) and *Y_u_*(*X* = 0) are never measured simultaneously (one subject cannot be treated and untreated at the same time), requiring an assumption called strong ignorability to identify causal effects. In our case, one could write $X_{t}^{j}$ as $X_{t}^{j}(X_{t}^{-})$, where time *t* replaces unit *u* and where we are interested in testing whether or not an entry $X_{t-\tau }^{i}$ in $X_{t}^{-}$ appears in the treatment function determining the causal influence from the past.

Both in Pearl’s causal effect framework and in the potential outcome framework (*3*), one typically assumes that the causal interdependency structure is known qualitatively (existence and absence of links), and the interest lies more in quantifying causal effects. In the case of linear continuous variables, the average causal effect and equivalently the potential outcome for $X_{t-\tau }^{i}\to X_{t}^{j}$ can be estimated from observational data with linear regression using a suitable adjustment set of regressors. In the following, we assume Causal Sufficiency and compare three approaches to estimate the linear causal effect when the true causal interdependency structure is unknown. CE-Corr simply is a univariate linear regression of $X_{t}^{j}$ on $X_{t-\tau }^{i}$, CE-Full is a multivariate regression of $X_{t}^{j}$ on the whole past of the multivariate process $X_{t}^{-}$ up to a maximum time lag τ_max_ = 5 and, finally, CE-PCMCI is a multivariate regression of $X_{t}^{j}$ on just the parents $P(X_{t}^{j})$ obtained with PCMCI.

In Fig. 6, we investigate these approaches numerically. Different from the model setup before, we now fix a network size of *N* = 20 time series variables (*T* = 150) and consider models with different link coefficients *c* (*x* axis in Fig. 6). The bottom panels show the distribution of causal effects. The absolute value of the true causal effect is ∣*c*∣ (black lines). The top panels show the distribution of true-positive rates (across all links in the model) for an *F*-test under the null hypothesis that the effect is zero at a significance level of 5%. The rightmost panels show the results for a regression on the true parents (CE-True).

CE-Corr values for links with the same causal effect span the whole range from zero to high effect values, indicating that CE-Corr is rather unrelated to the causal effect strength. Some CE-Corr values are much smaller and even tend to zero, which provides evidence for the observation in Fig. 5 that the detection power of correlation (or the other unconditional measures dCor and MI) can, counterintuitively, even be lower than that of FullCI or PCMCI. The distribution of CE-Full values is centered around the true causal effect as expected since $X_{t}^{-}$ includes the true parents as a sufficient adjustment set. However, the high dimensionality of this adjustment set leads to a large estimation variance that, in particular, implies that causal effects are less reliably estimated as evidenced by the low true-positive rates in the top panel. Last, CE-PCMCI better estimates causal effects and even comes close to the detection rate for CE-True based on the true parents. While the parents are a sufficient adjustment set to estimate causal effects, other adjustment sets may yield even better estimates, but in any case, knowledge of the dependency structure as estimated with PCMCI is beneficial (*54*).

## DISCUSSION AND CONCLUSION

Causal discovery on large-scale time series datasets is plagued by a dilemma: Including more variables makes an analysis more credible regarding a causal interpretation, but if the added variables are irrelevant, that is, not explanatory for causal relationships, they not only increase dimensionality but may also lead to smaller effect sizes, in particular, if they are caused by the considered driver variable. Both of these factors result in lower power and increase the risk that important true causal links are overlooked. Furthermore, some nonlinear tests do not even control false positives anymore in high dimensions.

Our method alleviates this problem by a condition selection stage to remove irrelevant variables and a conditional independence test designed for highly interdependent time series. The former improves power levels for large-scale causal discovery analyses, while the latter also yields more power than classical techniques in analyses involving only few variables, implying an improved “causal signal-to-noise ratio.” At the same time, the MCI test demonstrates correctly controlled false-positive rates even for highly autocorrelated time series data. Our numerical experiments show that PCMCI has significantly higher detection power than established methods such as Lasso, the PC algorithm, or Granger causality and its nonlinear extensions for time series datasets on the order of dozens to hundreds of variables. Further experiments indicate that PCMCI is robust to nonstationary trends, and all methods have a similar sensitivity to observational noise. PCMCI is not well suited for highly deterministic systems where not much new information is generated at each time step. In these cases, a state-space method gave higher detection power; however, we also found that it did not control false positives well. PCMCI allows accommodating a large variety of conditional independence tests adapted to different types of data (see section S4), for example, discrete or continuous time series.

Our causal effects analysis has demonstrated that a more reliable knowledge of the causal network also facilitates more precise estimates of the strength of causal links. There are different approaches to quantify causal strength from information-theoretic (*39*, *55*–*58*) to model-based measures such as the linear regression coefficients (*21*, *38*), as shown in the causal effect analysis. The MCI test statistic itself can be interpreted as a measure of causal strength (*39*, *55*), allowing us to directly rank causal links in exploratory PCMCI studies on large datasets with many time series in a meaningful way. These rankings can help to identify the strongest inferred causal links, which may be of main interest in some domain contexts. Next to assessing the causal strength of individual links, the estimated causal network can also be used to identify causal mediation pathways and estimate aggregate measures of the causal influence of individual variables (*38*, *39*, *58*).

Currently, our method focuses on time-lagged dependencies and assumes stationary data, and a causal interpretation rests, most importantly, on the assumption of Causal Sufficiency. This has several important implications for the practical use of PCMCI: For time-lagged dependencies, there is no ambiguity in terms of cause-effect directionality, that is, the orientation of causal edges. Recently, a growing body of literature addresses the inference of causality without relying on time lags (*59*, *60*), which could help to determine causal directionality for contemporaneous links.

The assumption of stationarity may be violated in real time series, for example, because of obvious confounders such as the seasonal cycle or different dynamical regimes underlying climate time series. In practice, time series can often be made stationary by removing or filtering these influences or by restricting the analysis to the part of the time series where stationarity can be assumed. In essence, these two approaches exploit some background knowledge on the cause of the nonstationarity. If, however, the causal dependency on a common nonstationarity is unknown, the resulting causal networks can contain spurious links (*34*). Our numerical results indicate that PCMCI is, however, more robust to nonstationarity than Lasso and PC.

As for any causal discovery method on observational data (*4*, *5*), Causal Sufficiency is probably the strongest assumption. Nonincluded or unobserved variables can still be the cause of a link in any nonexperimental analysis, which has to be taken into account for any scientific conclusions drawn. Potential causal links inferred from the available observational data can yield new hypotheses to be rejected or confirmed by further data analyses involving more variables (as illustrated in our climate example) or guide the design of numerical and real experiments. However, the finding of noncausality, that is, the absence of a causal link, relies on weaker assumptions (*34*): Given that the observed data faithfully represents the underlying process and that dependencies are powerfully enough captured by the test statistic, the absence of evidence for a statistical relationship makes it unlikely that a linking physical mechanism, in fact, exists. These findings of noncausality are, in that sense, more robust.

Growing data availability promises an unprecedented opportunity for novel insight through causal discovery across many disciplines of science—if the underlying assumptions are carefully taken into consideration and the methodological challenges are met (*2*). PCMCI addresses the challenges of large-scale multivariate, autocorrelated, linear, and nonlinear time series datasets opening up new opportunities to more credibly discover causal networks and estimate causal effects in many areas of science.

## MATERIALS AND METHODS

In this section, we explain the proposed causal discovery method in more detail and provide a description of real-world data. The Supplementary Materials contain implementation details of the alternative methods used in this work, details on the conditional independence tests, further theoretical discussions, a description of the numerical experiments setup, and pseudo-codes of algorithms, tables, and further figures.

PCMCI is implemented in the Tigramite open-source software package for Python, available from https://github.com/jakobrunge/tigramite. Tigramite contains classes for PCMCI and the different conditional independence tests, as well as a module that contains several plotting functions to generate high-quality plots of time series, lag functions, and causal graphs, as shown in Fig. 4. Tigramite also contains modules to estimate causal effects and analyze mediation pathways (*38*), as well as for selecting optimal predictors (*61*). Documentation can be found on the repository site.

### Detailed description of PCMCI

In our framework, the dependency structure of a set of time series variables is represented in a graphical model (*62*). While the process graph depicted in Fig. 1B is easier to visualize, it does not fully represent the spatiotemporal dependency structure underlying complex dynamical systems. Time series graphs (*32*–*34*) provide a more comprehensive view, as shown in Fig. 3. If, for example, graphical models are estimated without taking lagged variables into account, then associations can easily be confounded by the influence of common drivers at past times. For a formal definition of time series graphs, see section S1.

Our causal discovery technique to estimate the time series graph $\hat{G}$ is based on a two-stage procedure:

1. Condition selection via PC_1_: Obtain an estimate $\hat{P}(X_{t}^{j})$ of (a superset of) the parents $P(X_{t}^{j})$ for all variables $X_{t}^{j}\in X_{t}=(X_{t}^{1},X_{t}^{2},\ldots ,X_{t}^{N})$ with algorithm S1.

2. Use these parents as conditions in the MCI causal discovery stage (algorithm S2), which tests all variable pairs $\left(X_{t-\tau }^{i},X_{t}^{j}\right)$ with *i*, *j* ∈ {1, …, *N*} and time delays τ ∈ {1, …, τ_max_} and establishes a link, that is, $X_{t-\tau }^{i}\to X_{t}^{j}\in \hat{G}$, if and only if

MCI:Xt−τi⫫Xtj∣ Pˆ(Xtj)∖{Xt−τi},PˆpX(Xt−τi)  (3)

where ${\hat{P}}_{p_{X}}(X_{t-\tau }^{i})\subseteq \hat{P}(X_{t-\tau }^{i})$ denotes the *p_X_* strongest parents according to the sorting in algorithm S1. This parameter is just an optional choice. One can also restrict the maximum number of parents used for $\hat{P}(X_{t}^{j})$, but here, we impose no restrictions. For τ = 0, one can also consider undirected contemporaneous links (*39*).

Both stages, condition selection and MCI, consist of conditional independence tests. These tests can be implemented with different test statistics. Here, we used the tests ParCorr, GPDC, and CMI as detailed in section S4 and table S1.

PC_1_ in the first stage is a variant of the skeleton-discovery part of the PC algorithm (*25*) in its more robust modification called PC-stable (*36*) and adapted to time series. The algorithm is briefly discussed in the main text, more formally (pseudo-code in algorithm S1): For every variable, $X_{t}^{j}\in X_{t}$, first the preliminary parents $\hat{P}(X_{t}^{j})=(X_{t-1},X_{t-2},\ldots ,X_{t-\tau_{\text{max}}})$ are initialized. Starting with *p* = 0, iteratively *p* → *p* + 1 is increased in an outer loop and, in an inner loop, it is tested for all variables $X_{t-\tau }^{i}$ from $\hat{P}(X_{t}^{j})$ whether the null hypothesis

PC:Xt−τi⫫Xtj∣S for any S with ∣S∣=p (4)

can be rejected at a significance threshold α_PC_. For the PC algorithm implemented here, S iterates through different combinations of subsets of $\hat{P}(X_{t}^{j})\setminus \{X_{t-\tau }^{i}\}$ with cardinality *p*, up to a maximum number of combinations *q*_max_. Our fast variant PC_1_ is obtained by only testing the *p* parents with strongest dependency, that is, restricting the maximum number of combinations *q*_max_ per iteration to *q*_max_ = 1. In the first iteration (*p* = 0), S is empty and, thus, unconditional dependencies are tested. In each next iteration, the cardinality is increased *p* → *p* + 1, and Eq. 4 is tested again. If the null hypothesis cannot be rejected, then the link is removed from $\hat{P}(X_{t}^{j})$ at the end of each *p* iteration. The algorithm converges for a link $X_{t-\tau }^{i}\to X_{t}^{j}$ once $S=\hat{P}(X_{t}^{j})\setminus \{X_{t-\tau }^{i}\}$, and the null hypothesis $X_{t-\tau }^{i}⫫X_{t}^{j}|\;\hat{P}(X_{t}^{j})\setminus \{X_{t-\tau }^{i}\}$ is rejected (if the null hypothesis cannot be rejected, then the link is removed). $\hat{P}(X_{t}^{j})$ is sorted after every iteration according to the absolute test statistic value (ParCorr, GPDC, or CMI) and S is picked in lexicographic order (only relevant for *q*_max_ > 1). Other causal variable selection algorithms use similar heuristics (*35*, *63*). The MCI stage is inspired by the information-theoretic measure momentary information transfer introduced in different variants in (*55*, *64*).

The free parameters of PCMCI (in addition to free parameters of the conditional independence test statistic) are the maximum time delay τ_max_, the significance threshold α_PC_, and the maximum number *p_X_* of conditions of the driver variable in Eq. 3. We abbreviate different parameter choices by ${\text{PC}}_{1}^{\alpha }$+MCI*_p_X__*, if not clear from the context.

#### *Choice of* τ*_max_*

The maximum time delay depends on the application and should be chosen according to the maximum physical time lag expected in the complex system. If a relevant time lag, which may explain a dependency between two other variables, is not included, then the Causal Sufficiency assumption would be violated. In practice, we recommend a rather large choice, e.g., the last lag with significant unconditional dependency, because a too large choice of τ_max_ merely leads to longer runtimes of PCMCI but not so much to an increased estimation dimension as for FullCI.

#### *Choice of* α*_PC_*

α_PC_ should not be seen as a significance test level in PC_1_ since the iterative hypothesis tests do not allow for a precise assessment of uncertainties in this stage. α_PC_ here rather takes the role of a regularization parameter as in model selection techniques. The conditioning sets $\hat{P}$ estimated with PC_1_ should include the true parents and, at the same time, be small in cardinality to reduce the estimation dimension of the MCI test and improve its power. However, the first demand is typically more important (see section S5.3). In fig. S8, we investigated the performance of PCMCI implemented with ParCorr, GPDC, and CMI for different α_PC_. Too small values of α_PC_ result in many true links not being included in the condition set for the MCI tests and, hence, increase false positives. Too high levels of α_PC_ lead to high dimensionality of the condition set, which reduces detection power and increases the runtime. Note that for a threshold α_PC_ = 1 in PC_1_, no parents are removed and all *N*τ_max_ variables would be selected as conditions. Then, the MCI test becomes a FullCI test. As in any variable selection method (*35*), α_PC_ can be optimized using cross-validation or based on scores such as Bayesian Information Criterion (BIC) or AIC. For all ParCorr experiments (except for the ones labeled with ${\text{PC}}_{1}^{\alpha }$+MCI*_p_X__*), we optimized α_PC_ with AIC as a selection criterion. More precisely, for each $X_{t}^{j}$, we ran PC_1_ separately for each α_PC_ ∈ {0.1, 0.2, 0.3, 0.4}, yielding different conditioning sets ${\hat{P}}^{\alpha }(X_{t}^{j})$. Then, we fit a linear model for each α_PC_

$$X_{t}^{j}={\hat{P}}^{\alpha }(X_{t}^{j})\beta \;$$ (5)

yielding the residual sum of squares (RSS), and selected α_PC_ according to AIC (modulo constants)

$$\alpha_{\text{PC}}^{*}={\text{argmin}}_{\alpha_{\text{PC}}}n\text{log}\;({\mathrm{RSS}}^{\alpha })+2|{\hat{P}}^{\alpha }(X_{t}^{j})|\;$$ (6)

where *n* is the sample size (typically the time series length *T* minus a cutoff due to τ_max_) and ∣ · ∣ denotes cardinality. For GPDC, one can similarly select α_PC_ based on the log marginal likelihood of the fitted Gaussian process, while for CMI, one can use cross-validation based on nearest-neighbor predictions for different ${\hat{P}}^{\alpha }(X_{t}^{j})$. But since GPDC and CMI are already quite computationally demanding, we picked α_PC_ = 0.2 in all experiments, based on our findings in fig. S8. In the bottom panels of figs. S4 to S7, we analyzed α_PC_ = 0.2 also for ParCorr for all numerical experiments and found that this option also gave good results for sparse networks and runs even faster than Lasso. However, there is potentially a higher risk of inflated false positives. Unfortunately, we have no finite sample consistency results for choosing α_PC_.

#### Choice of p_X_

While the parents $\hat{P}(X_{t}^{j})$ are sufficient to assess conditional independence, the additional conditions ${\hat{P}}_{p_{X}}(X_{t-\tau }^{i})\subseteq \hat{P}(X_{t-\tau }^{i})$ are used to account for autocorrelation and make the MCI test statistic a measure of causal strength as analyzed in section S5.5. To limit high dimensionality, one can strongly restrict the number of conditions ${\hat{P}}_{p_{X}}(X_{t-\tau }^{i})$ with the free parameter *p_X_*. To avoid having another free parameter, we kept *p_X_* unrestricted in most experiments. In some experiments (see figs. S4, S5, S6, S7, S10, and S12), we found that a small value *p_X_* = 3 already suffices to reduce inflated false positives due to strong autocorrelation and estimate causal strength. The reason is that, typically, the largest driver will be the autodependency, and conditioning out its influence already diminishes the effect of strong autocorrelations. In theory, a too small *p_X_* should lead to a less well-calibrated test (see section S5.3), but in practice, it seems like a sensible trade-off. In section S3, we describe a PCMCI variant for *p_X_* = 0 and a bivariate variant that does not condition on external variables. Both of these cannot guarantee consistent causal graph estimates and likely feature inflated false positives especially for strong autocorrelation.

#### False discovery rate control

PCMCI can also be combined with false discovery rate controls, e.g., using the Hochberg-Benjamini approach (*37*). This approach controls the expected number of false discoveries by adjusting the *P* values resulting from the MCI stage for the whole time series graph. More precisely, we obtain the *q* values as

$$q\;=\;\text{min}\;(P\frac{m}{r},1)$$ (7)

where *P* is the original *P* value, *r* is the rank of the original *P* value when *P* values are sorted in ascending order, and *m* is the number of computed *P* values in total, that is, *m* = *N*^2^τ_max_ to adjust only directed links for τ > 0 and correspondingly if also contemporaneous links for τ = 0 are taken into account. In our numerical experiments, we did not control the false discovery rate since we were interested in the individual link performances.

### Real-world applications

The climate time series are regional averages (see boxes in Fig. 4) from the reanalysis (*65*) for the period 1948–2012 with 780 months. WPAC denotes monthly surface pressure anomalies in the West Pacific, CPAC and EPAC surface air temperature anomalies in the Central and East Pacific, respectively, and ATL surface air temperature anomalies in the tropical Atlantic. Anomalies are taken with respect to the whole period. The data are freely available from www.esrl.noaa.gov.

The cardiovascular analysis is based on an ensemble of 13 datasets of healthy pregnant women as studied in (*50*), where the data are described in detail. The study was approved by the local ethics committee, and it obtained the informed consent of all of the subjects. The time series contain 600 samples (cutting of a transient of 300) and are sampled at heart beats. *B* denotes the time series of intervals between successive heart beats, and *D* the diastolic and *S* the systolic blood pressure.

### Further information in the Supplementary Materials

A more detailed definition of time series graphs is given in section S1. Section S2 details the alternative methods FullCI, Lasso, PC algorithm, CCM, and the unconditional correlation, distance correlation, and MI. Section S3 discusses further variants of PCMCI, one variant that excludes the conditioning on the parents of the driver variable, i.e., *p_X_* = 0, and another variant that excludes conditioning on external variables. The conditional independence tests used here (ParCorr, GPDC, and CMI), which form the basis of PCMCI, the PC algorithm, and FullCI, are in detail explained in section S4. Theoretical properties of PCMCI are discussed in section S5. In particular, the polynomial computational complexity is derived in section S5.1, consistency is proven in section S5.2, and section S5.3 expands on the correct control of false positives at the specified significance level, also in the presence of strong autocorrelation. Section S5.4 proves that MCI is larger than or equal to FullCI and explains how conditioning on irrelevant variables reduces effect size for FullCI. The interpretation of MCI as a notion of causal strength is given in section S5.5. The detailed setup of numerical experiments is laid out in section S6, including AN(C)OVA analyses and performance metrics. The remaining part of the Supplementary Materials provides pseudo-codes of algorithms and tables and further figures as referenced in the main text.

## Supplementary Material

http://advances.sciencemag.org/cgi/content/full/5/11/eaau4996/DC1

Download PDF

Detecting and quantifying causal asociations in large nonlinear time series datasets

## SUPPLEMENTARY MATERIALS

Supplementary material for this article is available at http://advances.sciencemag.org/cgi/content/full/5/11/eaau4996/DC1 Section S1. Time series graphs Section S2. Alternative methods Section S3. Further PCMCI variants Section S4. Conditional independence tests Section S5. Theoretical properties of PCMCI Section S6. Numerical experiments Algorithm S1. Pseudo-code for condition selection algorithm. Algorithm S2. Pseudo-code for MCI causal discovery stage. Algorithm S3. Pseudo-code for adaptive Lasso regression. Table S1. Overview of conditional independence tests. Table S2. Model configurations for different experiments. Table S3. Overview of methods compared in numerical experiments. Table S4. Summarized ANOVA results for high-dimensionality ParCorr experiments. Table S5. Summarized ANOVA results for high-density ParCorr experiments. Table S6. Summarized ANOVA results for high-dimensionality GPDC and CMI experiments. Table S7. Summarized ANOVA results for sample size experiments. Table S8. Summarized ANOVA results for noise and nonstationarity experiments. Table S9. ANCOVA results for FullCI. Table S10. ANCOVA results for Lasso. Table S11. ANCOVA results for PC. Table S12. ANCOVA results for FullCI. Fig. S1. Illustration of notation. Fig. S2. Motivational climate example. Fig. S3. Real climate and cardiovascular applications. Fig. S4. Experiments for linear models with short time series length. Fig. S5. Experiments for linear models with longer time series length. Fig. S6. Experiments for dense linear models with short time series length. Fig. S7. Experiments for dense linear models with longer time series length. Fig. S8. Experiments for different method parameters. Fig. S9. Experiments for linear methods with different sample sizes. Fig. S10. Experiments for nonlinear models (part 1). Fig. S11. Experiments for nonlinear models with different sample sizes (part 1). Fig. S12. Experiments for nonlinear models (part 2). Fig. S13. Experiments for nonlinear models with different sample sizes (part 2). Fig. S14. Experiments for observational noise models. Fig. S15. Experiments for nonstationary models. Fig. S16. Runtimes for numerical experiments. Fig. S17. ANCOVA interaction plots. Fig. S18. Comparison of PCMCI and CCM on logistic maps. References (*66*–*80*)
