# Supplementary material for: Detecting and quantifying causal associations in large nonlinear time series datasets
Source: Sci Adv. 2019 Nov 27;5(11):eaau4996. doi: 10.1126/sciadv.aau4996 (PMC6881151; doi:10.1126/sciadv.aau4996)
Supplement: http://advances.sciencemag.org/cgi/content/full/5/11/eaau4996/DC1 [file supp_5_11_eaau4996__index.html]

Science Advances | Science AdvancesAAASSearchScience AdvancesMenu

## Supplementary Materials

**This PDF file includes:**

- Section S1. Time series graphs
- Section S2. Alternative methods
- Section S3. Further PCMCI variants
- Section S4. Conditional independence tests
- Section S5. Theoretical properties of PCMCI
- Section S6. Numerical experiments
- Algorithm S1. Pseudo-code for condition selection algorithm.
- Algorithm S2. Pseudo-code for MCI causal discovery stage.
- Algorithm S3. Pseudo-code for adaptive Lasso regression.
- Table S1. Overview of conditional independence tests.
- Table S2. Model configurations for different experiments.
- Table S3. Overview of methods compared in numerical experiments.
- Table S4. Summarized ANOVA results for high-dimensionality ParCorr experiments.
- Table S5. Summarized ANOVA results for high-density ParCorr experiments.
- Table S6. Summarized ANOVA results for high-dimensionality GPDC and CMI experiments.
- Table S7. Summarized ANOVA results for sample size experiments.
- Table S8. Summarized ANOVA results for noise and nonstationarity experiments.
- Table S9. ANCOVA results for FullCI.
- Table S10. ANCOVA results for Lasso.
- Table S11. ANCOVA results for PC.
- Table S12. ANCOVA results for FullCI.
- Fig. S1. Illustration of notation.
- Fig. S2. Motivational climate example.
- Fig. S3. Real climate and cardiovascular applications.
- Fig. S4. Experiments for linear models with short time series length.
- Fig. S5. Experiments for linear models with longer time series length.
- Fig. S6. Experiments for dense linear models with short time series length.
- Fig. S7. Experiments for dense linear models with longer time series length.
- Fig. S8. Experiments for different method parameters.
- Fig. S9. Experiments for linear methods with different sample sizes.
- Fig. S10. Experiments for nonlinear models (part 1).
- Fig. S11. Experiments for nonlinear models with different sample sizes (part 1).
- Fig. S12. Experiments for nonlinear models (part 2).
- Fig. S13. Experiments for nonlinear models with different sample sizes (part 2).
- Fig. S14. Experiments for observational noise models.
- Fig. S15. Experiments for nonstationary models.
- Fig. S16. Runtimes for numerical experiments.
- Fig. S17. ANCOVA interaction plots.
- Fig. S18. Comparison of PCMCI and CCM on logistic maps.
- References (*66*–*80*)

Download PDF

**Files in this Data Supplement:**

- Adobe PDF - aau4996\_SM.pdf
