# Supplementary material for: Detecting and quantifying causal associations in large nonlinear time series datasets
Source: Sci Adv. 2019 Nov 27;5(11):eaau4996. doi: 10.1126/sciadv.aau4996 (PMC6881151; doi:10.1126/sciadv.aau4996)
Supplement: Download PDF [file aau4996_SM.pdf]

Science Advances
AAAS

advances.sciencemag.org/cgi/content/full/5/11/eaau4996/DC1

Supplementary Materials for

Detecting and quantifying causal associations in large nonlinear time series datasets

Jakob Runge*, Peer Nowack, Marlene Kretschmer, Seth Flaxman, Dino Sejdinovic

*Corresponding author. Email: jakob.runge@dlr.de

Published 27 November 2019, *Sci. Adv.* **5**, eaau4996 (2019)
DOI: 10.1126/sciadv.aau4996

This PDF file includes:

Section S1. Time series graphs
Section S2. Alternative methods
Section S3. Further PCMCI variants
Section S4. Conditional independence tests
Section S5. Theoretical properties of PCMCI
Section S6. Numerical experiments
Algorithm S1. Pseudo-code for condition selection algorithm.
Algorithm S2. Pseudo-code for MCI causal discovery stage.
Algorithm S3. Pseudo-code for adaptive Lasso regression.
Table S1. Overview of conditional independence tests.
Table S2. Model configurations for different experiments.
Table S3. Overview of methods compared in numerical experiments.
Table S4. Summarized ANOVA results for high-dimensionality ParCorr experiments.
Table S5. Summarized ANOVA results for high-density ParCorr experiments.
Table S6. Summarized ANOVA results for high-dimensionality GPDC and CMI experiments.
Table S7. Summarized ANOVA results for sample size experiments.
Table S8. Summarized ANOVA results for noise and nonstationarity experiments.
Table S9. ANCOVA results for FullCI.
Table S10. ANCOVA results for Lasso.
Table S11. ANCOVA results for PC.
Table S12. ANCOVA results for FullCI.
Fig. S1. Illustration of notation.
Fig. S2. Motivational climate example.
Fig. S3. Real climate and cardiovascular applications.
Fig. S4. Experiments for linear models with short time series length.
Fig. S5. Experiments for linear models with longer time series length.
Fig. S6. Experiments for dense linear models with short time series length.
Fig. S7. Experiments for dense linear models with longer time series length.
Fig. S8. Experiments for different method parameters.

Fig. S9. Experiments for linear methods with different sample sizes.
Fig. S10. Experiments for nonlinear models (part 1).
Fig. S11. Experiments for nonlinear models with different sample sizes (part 1).
Fig. S12. Experiments for nonlinear models (part 2).
Fig. S13. Experiments for nonlinear models with different sample sizes (part 2).
Fig. S14. Experiments for observational noise models.
Fig. S15. Experiments for nonstationary models.
Fig. S16. Runtimes for numerical experiments.
Fig. S17. ANCOVA interaction plots.
Fig. S18. Comparison of PCMCI and CCM on logistic maps.
References (*66–80*)

Section S1. Time series graphs

Definition 1 (Definition of time series graph)**.** *Let* $\mathbf{X}$ *be a multivariate discrete-time stochastic process and* $\mathcal{G} = (\mathbf{X} \times \mathbb{Z}, E)$ *the associated time series graph. The nodes in that graph are the individual time-dependent variables* $\mathbf{X} = (X^1, \ldots, X^N)$ *at each time* $t \in \mathbb{Z}$*. Let* $\mathbf{X}_t$ *denote the process at a particular time step that we call the* present*, and* $\mathbf{X}_t^- = (\mathbf{X}_{t-1}, \mathbf{X}_{t-2}, \ldots)$ *the past process. Consider an underlying time-dependent data-generating process*

$$X_t^j = f_j\left(\mathcal{P}(X_t^j), \eta_t^j\right) \quad j \in \{1, \ldots, N\} \tag{S1}$$

where f_j *is some potentially nonlinear functional dependency and* η_t^j *represents mutually independent dynamical noise, i.e.,* $\eta_t^j \perp\!\!\!\perp \eta_{t'}^k$ *for* $k \neq j$ *or* $t \neq t'$*.* $\mathcal{P}(X_t^j) \subset \mathbf{X}_t^-$ *denotes the causal parents of variable* X_t^j*. Variables* $X_{t-\tau}^i$ *and* X_t^j *for* $\tau > 0$ *are connected by a lag-specific* directed link *"*$X_{t-\tau}^i \to X_t^j$*" in* $\mathcal{G}$ *pointing forward in time if* $X_{t-\tau}^i \in \mathcal{P}(X_t^j)$*.*

Another way to define the edges E of the graph is

$$X_{t-\tau}^i \not\perp\!\!\!\perp X_t^j \mid \mathbf{X}_t^- \setminus \{X_{t-\tau}\} \tag{S2}$$

for $\tau > 0$ and where '$\not\perp\!\!\!\perp$' denotes the absence of a conditional independence and $\mathbf{X}_t^- \setminus \{X_{t-\tau}^i\}$ the past of the multivariate process excluding $X_{t-\tau}^i$. Both definitions are equivalent for processes sufficing the assumptions used here (section "Assumptions of causal discovery from observational data"), in particular the Markov property which holds since the noise terms η in Eq. (S1) are assumed independent.
The graph is actually infinite in time, but in practice only considered up to some maximum time lag $\tau_{\max}$. Throughout this work we assume that we have only one time series realization available and assume stationarity: The above definition for links at time t holds for links at every $t' \in \mathbb{Z}$. Then the parents $\mathcal{P}(X_t^j)$ for all variables $X_t^j \in \mathbf{X}_t$ represent the graph $\mathcal{G}$. Contemporaneous links for $\tau = 0$ ($\mathcal{P}(X_t^j) \subset \mathbf{X}_{t+1}^-$ in Eq. (S1)) can be defined in different ways (*34, 39*). Here they are left undirected, but other techniques (*59, 60*) could be applied to determine causal directionality for contemporaneous links.

Section S2. Alternative methods

In the following we define alternative causal and non-causal methods considered here, Supplementary Tab. S3 gives an overview of the methods compared in the numerical experiments.

S2.1 FullCI

The most straightforward way to test the existence of causal links is to directly test

$$\text{FullCI:} \quad X_{t-\tau}^i \not\perp\!\!\!\perp X_t^j \mid \mathbf{X}_t^- \setminus \{X_{t-\tau}^i\} \tag{S3}$$

As before, the past $\mathbf{X}_t^-$ is truncated at a maximum time lag $\tau_{\max}$. Also this test can be implemented with any of the conditional independence tests defined in Supplementary Sect. S4 below.
In its original formulation, Granger causality between time series variables X and Y is based on fitting a linear or nonlinear model, including other covariates, to Y and a causal link $X \to Y$ is assessed by quantifying whether the inclusion of the past of variable X in the model significantly reduces the prediction error about Y (*12*). Our formulation can be interpreted as a general, lag-specific version. For linear models we implement FullCI by fitting a vector-autoregressive (VAR) model using the `statsmodels` package, which also allows to obtain the corresponding p-values.

S2.2 Lasso

In the linear case, FullCI can be phrased as testing for nonzero coefficients in a VAR model. If implemented using standard ordinary least-square regression, the problem becomes ill-defined if the number of coefficients exceeds the number of samples. One way to address this problem are *regularized* high-dimensional regression techniques. In particular, Lasso (*17*) is a common regression method that can also be used for variable selection. Lasso regression is known to be inconsistent in some scenarios, which is overcome by the *Adaptive Lasso* (*18*) that yields a consistent estimator by utilizing an adaptively weighted penalty. Here, we implemented an adaptive Lasso that consists of computing several Lasso regressions with iterative feature re-weighting, see Supplementary Algorithm S3. After several iterations, the active set of variables is determined as the non-zero coefficients. Then all zero-coefficients are assigned a p-value of one, while the p-values for the active set of variables is determined by an OLS regression including only the active variables. To select the optimal regularization parameter, we used a time-series based cross-validation scheme. Some tests based on AIC-hyperparameter-selection did not yield good results. We found that the p-values tend to be over-conservative and false positive levels cannot be reliably controlled just below a desired threshold.

S2.3 PC algorithm

The original PC algorithm was formulated for general random variables without assuming a time order. It consists of several phases where first, in the skeleton-discovery phase, an undirected graphical model (*62*) is estimated whose links are then oriented using a set of rules (*5, 25*) in subsequent phases. Since here time order yields an orientation of time-lagged links, we implement only the skeleton-discovery phase of the PC algorithm, as given in Supplementary Algorithm S1, but in contrast to our fast variant, the original version does not restrict the number of condition combinations $q_{\max}$ to test. In our numerical experiments, we use a large choice of $q_{\max} = 10$ and $\alpha_{\mathrm{PC}} = 0.2$. In contrast to FullCI or PCMCI, it is not straightforward to assess the confidence of causal links with the PC algorithm, because links are iteratively removed. Here we use a p-value assessment for causal links according to ref. (*66*)

$$p(X^i_{t-\tau} \to X^j_t) = \max_{\{\mathcal{S}\}} p\left(X^i_{t-\tau} \perp\!\!\!\perp X^j_t | \mathcal{S}\right) \tag{S4}$$

that is, the maximum of all p-values from the conditional independence tests for different condition sets $\mathcal{S}$ in Eq. (4) defines the aggregated p-value of a causal link. This causal discovery method we term PC in the numerical experiments. Also here, we found that the p-values tend to be over-conservative for the most part with outliers for strong autocorrelations, and that false positive levels cannot be reliably controlled just below a desired threshold.

S2.4 Convergent cross-mapping

Convergent cross-mapping (CCM) (*14*) assumes an underlying dynamical system and is based on state-space reconstruction (see also refs. (*15, 53,71*)). In this framework a causal relationship between two dynamical variables X and Y can be established if they belong to a common dynamical system. If variable X can be predicted using the reconstructed system based on the time-delay embedding of variable Y, then X has a causal effect on Y. CCM is here estimated with embedding dimension $E = 2$ and alternatively by optimizing the embedding dimension using the simplex algorithm (*14*). To test significance we used the surrogate test `ebisuzaki` with 500 surrogates in the R-package `rEDM`. CCM requires two criteria (*14*): (1) a significant CCM value at library size n and (2) an increasing CCM

value over increasing library length. As a p-value of CCM we, hence, take $\max(p_n, p_{conv})$, where p_n is the p-value of CCM at library size n and p_{conv} is the p-value for the hypothesis of an increasing linear trend of the CCM value with increasing library length, here from $n' = 10$ to $n' = n$. Note that to test $X \to Y$, CCM and related works (*53*) only use the time series of X and Y with the underlying assumption that the dynamics of any common driver can be reconstructed using delay embedding. In Supplementary Fig. S18 we depict a comparison with PCMCI on coupled logistic maps described in Supplementary Sect. S6 below.

S2.5 Unconditional pairwise tests

We also investigate unconditional pairwise test measures $I(X_{t-\tau}; Y_t)$. In the ParCorr implementation this is simply the Pearson correlation coefficient (Corr), in the GPDC implementation the distance correlation (dCor), and for CMI the mutual information (MI), see definitions in Supplementary Sect. S4.

Section S3. Further PCMCI variants

S3.1 PCMCI$_0$

In the MCI stage of the PCMCI method, one can also test

$$\text{MCI}_0: \quad X^i_{t-\tau} \not\perp\!\!\!\perp X^j_t \mid \mathcal{P}(X^j_t) \setminus \{X^i_{t-\tau}\} \tag{S5}$$

for all links, that is, the MCI test without the condition on the parents $\widehat{\mathcal{P}}(X^i_{t-\tau})$, or, equivalently, $p_X = 0$, denoted PC$_1$+MCI$_0$. Note that for $X^i_{t-\tau} \in \widehat{\mathcal{P}}(X^j_t)$ this is the test in the last iteration of the PC algorithm, but here also links that were removed in PC$_1$ are tested again. In our numerical experiments (Supplementary Figs. S4,S5,S6,S7) we found that PC$_1$+MCI$_0$ has inflated false positives.
We also test a variant, called PC$_1$+MCI$_0$pw, where all time series are pre-whitened prior to running PC$_1$+MCI$_0$, that is, we preprocessed all N time series by estimating the univariate lag-1 autocorrelation coefficients $\hat{a}_i = \rho(X^i_{t-1}; X^i_t)$ and regressing out the AR(1) autocorrelation part of the signals

$$\tilde{X}^i_t = X^i_t - \hat{a}_i X^i_{t-1} \quad \forall t \text{ and } i \in \{1, \ldots, N\} \tag{S6}$$

Then the PC$_1$+MCI$_0$ test is applied to these residuals $\tilde{\mathbf{X}}$. Our numerical results in Supplementary Figs. S4,S5,S6,S7 show that this approach also fails to control false positives.

S3.2 BivCI

In the numerical experiments, we also evaluate a bivariate conditional independence test (BivCI)

$$\text{BivCI:} \quad X^i_{t-\tau} \not\perp\!\!\!\perp X^j_t \mid X^{j-}_t \tag{S7}$$

where $X^{j-}_t = (X^j_{t-1}, X^j_{t-2}, \ldots, X^j_{t-\tau_{\max}})$ denotes the past of X^j_t. This corresponds to a pairwise lag-specific version of Granger causality or Transfer Entropy (*67*). Thus, this test removes autocorrelation to some extent, but does not exclude common drivers or indirect dependencies. Also auto-dependencies induced by common drivers are not removed. As analyzed in Supplementary Figs. S4,S5,S6,S7, BivCI reduces autocorrelation effects to some extent, but false positives are not correctly controlled, as expected.

Section S4. Conditional independence tests

Similarly to the PC algorithm, the PCMCI framework (Supplementary Algorithms S1 and S2) that we propose can be used in conjunction with any conditional independence test – these will typically be based on estimating different dependence measures with associated test statistics. Here we implement three tests: partial correlation (ParCorr), a nonlinear two-step conditional independence test we term GPDC, and a fully non-parametric test based on conditional mutual information (*44*). Supplementary Tab. S1 gives an overview of the tests. Note that the conditional independence tests in the present context are conducted with a sample size of $n = T - 2\tau_{\max}$, where T is the time series length and the first $2\tau_{\max}$ samples are cut off since this is the maximum time lag of a lagged parent in $\widehat{\mathcal{P}}(X^i_{t-\tau})$ relative to X^j_t. Further, we might need to account for missing and masked samples.

S4.1 ParCorr

Partial correlation for testing $X \perp\!\!\!\perp Y \mid \mathbf{Z}$ here is estimated (Supplementary Tab. S1) in a two-stage procedure with a multivariate regression of X and Y, separately, on $\mathbf{Z}$ followed by a correlation test on the residuals. Its advantages are fast computation and that the distribution under the null hypothesis of conditional independence is known analytically, but it is applicable only to the multivariate Gaussian case which can only capture linear dependencies.

S4.2 GPDC

GPDC also belongs to the class of residual-based conditional independence tests. Instead of a linear regression, here the first step is a Gaussian process (GP) regression (*42*) and the second step a test for the (unconditional) independence of the uniformized residuals with the *distance correlation coefficient* (DC) (*43*). GP regression is a widely used Bayesian nonparametric regression approach. Distance correlation (*43*) is a measure of dependence between two random variables and is zero if and only if the variables are independent. Thus, distance correlation measures both linear and nonlinear association. See Supplementary Tab. S1 for details. Note that the underlying assumption of GPDC is that of an additive functional dependency, see ref. (*72*) for a more general, but still residual-based, test. The estimator for distance correlation is defined as

$$\mathrm{dCor}(X,Y) = \frac{\mathrm{dCov}(X,Y)}{\sqrt{\mathrm{dVar}(X)\ \mathrm{dVar}(Y)}} \tag{S8}$$

where the distance covariance dCov (and variance dVar) is computed by

$$\mathrm{dCov}^2(X,Y) = \frac{1}{n^2}\sum_{j=1}^{n}\sum_{k=1}^{n} A_{j,k}\, B_{j,k} \tag{S9}$$

where $A_{j,k}\, B_{j,k}$ are the doubly-centered distance matrices of X and Y (see ref. (*43*)), respectively, and n is the sample size (in the present context the length of the time series minus cutoffs due to $\tau_{\max}$). Distance correlation is implemented in `Tigramite` based on the code in the original `dcov.test` function in the energy package for the R-language. The prior transformation of X and Y to uniform marginals allows to pre-compute the distribution for each sample size n (implemented in `Tigramite`) and critical values under the independence null hypothesis – thereby avoiding computationally expensive permutation tests for each test of $X \perp\!\!\!\perp Y \mid \mathbf{Z}$. Note that since we are not aware of a way to account for the GP-step

in assessing the degrees of freedom for the subsequent distance correlation test, we simply used the sample size n, which did not seem to inflate false positives in our experiments, at least for not too high dimensions. Figure 3D illustrates a quadratic relationship $X = Z^2 + \eta^X$ and $Y = -Z^2 + \eta^Y$ for $Z \sim \mathcal{N}(0,1)$ where $X \perp\!\!\!\perp Y \mid Z$ can be detected with GPDC, but not with ParCorr.

S4.3 CMI

ParCorr and GPDC, like any two-step procedure for conditional independence testing, which includes a regression followed by an unconditional test on the regression residuals, has an underlying assumption of additive noise and a functional dependence on the conditioning variables. In the presence of dependencies which cannot be represented even in a nonlinear functional form, a regression will not be able to remove these dependencies on the conditioning variables. Figure 3D for CMI illustrates a multiplicative case with $X = Z\eta^X$ and $Y = -Z\eta^Y$ for $Z \sim \mathcal{N}(0,1)$, where both ParCorr and GPDC fail to establish $X \perp\!\!\!\perp Y \mid Z$. Then the two-step procedure should be replaced with fully non-parametric techniques to measuring and testing conditional dependence. Here, a fully non-parametric test (*44*) for continuous data based on conditional mutual information, combined with a local permutation scheme is implemented. The conditional mutual information for continuous and possibly multivariate random variables X, Y, Z is defined as

$$I(X;Y|Z) = \iiint dxdydz\ p(x,y,z) \log \frac{p(x,y|z)}{p(x|z)\cdot p(y|z)} \tag{S10}$$

where we have to assume that the densities $p(\cdot)$ exist. From this definition it is immediately clear that the CMI is zero if and only if $X \perp\!\!\!\perp Y|Z$. From the nearest-neighbor entropy estimator by Kozachenko and Leonenko (*73*), Kraskov et al. (*74*) developed an estimator for mutual information that was generalized to CMI (*75, 76*):

$$\widehat{I}(X;Y|Z) = \psi(k) + \frac{1}{n}\sum_{i=1}^{n}\left[\psi(k_i^z) - \psi(k_i^{xz}) - \psi(k_i^{yz})\right] \tag{S11}$$

with the Digamma function as the logarithmic derivative of the Gamma function $\psi(x) = \frac{d}{dx}\ln\Gamma(x)$ and sample length n. The only free parameter k is the number of nearest neighbors in the joint space of $\mathcal{X}\otimes\mathcal{Y}\otimes\mathcal{Z}$ which defines the local length scale (in maximum norm) ϵ_i around each sample point i. Then k_i^{xz}, k_i^{yz} and k_i^z are computed by counting the number of points with distance strictly smaller than ϵ_i (including the reference point i) in the subspace $\mathcal{X}\otimes\mathcal{Z}$ to get k_i^{xz}, in the subspace $\mathcal{Y}\otimes\mathcal{Z}$ to get k_i^{yz}, and in the subspace $\mathcal{Z}$ to get k_i^z. The decisive advantage of this estimator compared to fixed global bandwidth approaches is its local *data-adaptiveness* (Fig. 3D): The hypercubes around each sample point are smaller where more samples are available. As opposed to GP regression, this feature allows to detect also highly non-smooth dependencies (*44*). Unfortunately, few theoretical results are available for the complex mutual information estimator. While the Kozachenko-Leonenko estimator is asymptotically unbiased and consistent (*73, 77*), the variance and finite sample convergence rates are unknown. Hence, the null distribution in the CMI test (*44*) relies on a local permutation test that is also based on nearest neighbors and data-adaptive. Here we set $k_{\mathrm{CMI}} = 50$, and as the nearest neighbors in the local permutation scheme $k_{\mathrm{perm}} = 5$, as well as $B = 500$ for the number of surrogates to approximate the null distribution. These choices are based on the findings in ref. (*44*). CMI is implemented in `Tigramite`, where $k_{\mathrm{CMI}} = \lfloor 0.1n \rfloor$ with sample size n, $B = 500$, and $k_{\mathrm{perm}} = 5$ are the default parameters. Alternative conditional independence tests are, e.g., kernel conditional independence tests (*78–80*). PCMCI-CMI

can be considered a *doubly adaptive* causal discovery method: PCMCI adapts the condition-selection locally to the causal network and CMI adapts the conditional independence estimation locally to the sample density.

S4.4 Discrete data

The previous tests were developed for continuously-valued data. For discrete or symbolic data, the software package `Tigramite` implements a CMI test based on discrete entropy estimation, called CMIsymb. This test directly estimates CMI based on

$$\widehat{I}(X;Y|Z) = \sum_{x,y,z} \widehat{p}(x,y,z) \log \frac{\widehat{p}(x,y,z)\widehat{p}(z)}{\widehat{p}(x,z)\widehat{p}(y,z)} \tag{S12}$$

where the discrete densities are estimated from symbol frequencies. For this test no analytical results for the null distribution are available and a permutation test is recommended.

Section S5. Theoretical properties of PCMCI

S5.1 Computational complexity

PCMCI has polynomial complexity in the number of variables N and $\tau_{\max}$. The computational complexity of the PC_1 stage of PCMCI (Supplementary Algorithm S1) strongly depends on the network structure and the parameter α_{PC}. The sparser the causal dependencies, the faster the convergence. In the worst case, where the network is completely connected (which is rather pathological), the computational complexity of the PC_1 condition-selection stage for N variables amounts to

$$N \sum_{p=0}^{N\tau_{\max}-1} N\tau_{\max} = N^3 \tau_{\max}^2 \tag{S13}$$

conditional independence tests with iteratively increasing cardinality. The MCI stage (Supplementary Algorithm S2) further involves $N^2\tau_{\max}$ tests (for $\tau > 0$). Hence, the worst case total computational complexity in the number of variables is polynomial and given by $N^3\tau_{\max}^2 + N^2\tau_{\max}$–if α_{PC}-optimization is not taken into account, which yields a factor of the number of α_{PC}-values tested in the first term. The maximal dimensionality of the estimations in the MCI stage is $2 + |\widehat{\mathcal{P}}(X^j_t)| + |\widehat{\mathcal{P}}(X^i_{t-\tau})|$. The computational runtime will then depend on how the conditional independence test scales with this dimensionality and the time series length T. In the numerical experiments, we analyze runtimes of the different compared methods for different network sizes N and time series lengths T (Supplementary Fig. S16). In `Tigramite` a `recycle_residuals`-option is implemented that can be used for ParCorr and GPDC to save already computed residuals in memory to be re-used in later tests of PC_1 or MCI.

S5.2 Consistency

We here give a proof of the consistency for the population version of PCMCI, that is, PCMCI estimates the true graph if there are no errors in the conditional independence tests. The proof relies on the three standard assumptions in causal discovery (*5*): (1) *Causal Sufficiency*, implying that there exist no other unobserved variables that directly or indirectly influence any other pair of observed variables, (2) the

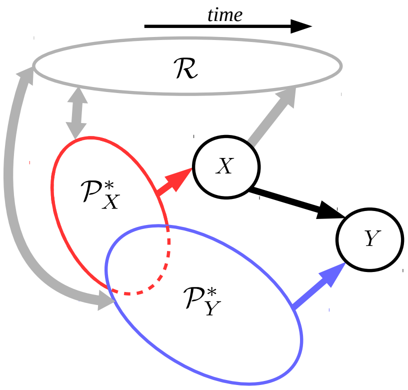

Fig. S1. **Illustration of notation for proving Proposition 1 and Proposition 3.**

Causal Markov Condition, implying that X^j_t is independent of $\mathbf{X}^-_t \setminus \mathcal{P}(X^j_t)$ given its parents $\mathcal{P}(X^j_t)$, and (3) *Faithfulness* which guarantees that the graph entails *all* conditional independence relations that are implied by the Markov condition. Faithfulness says that if two variables are independent conditionally on a set $\mathcal{S}$, then they are also *separated* by $\mathcal{S}$ in the graph. See ref. (*34*) for formal definitions of separation and the assumptions. As part of the Causal Markov Condition in the time series graph context, we also assume *no contemporaneous causal effects*, which excludes causal effects at $\tau = 0$. Only then the lagged parents are sufficient for the Causal Markov condition. Last, we also assume stationarity here so that dependencies (or lack of them) remain unchanged across time. With these assumptions we can state the consistency of PCMCI.

Proposition 1. *(Consistency) Let* $\mathbf{X}$ *be a stochastic process with true time series graph* $\mathcal{G}$ *as defined in Def. 1 (equivalent to definition in Eq. S2) and let* $\widehat{\mathcal{G}}$ *be the estimated graph with PCMCI (Algorithms S1,S2) implemented with a consistent conditional independence test. Assuming Causal Sufficiency, Faithfulness and the Causal Markov Condition, we have that*

$$\widehat{\mathcal{G}} = \mathcal{G} \tag{S14}$$

A proof is given below. Note that the consistency of the population-version of PCMCI is a weaker statement than, for example, *uniform consistency* which bounds the error probability as a function of the sample size giving a *rate of convergence*. Robins *et al.* (*45*) showed that no uniformly consistent causal discovery technique from the class of independence-based methods (*5*) exists, since the convergence can always be made arbitrarily slow by a distribution that is *almost unfaithful* with some dependencies made arbitrarily small. Uniform consistency can only be achieved under further assumptions that exclude these almost unfaithful dependencies (*46*).
To prove consistency, we need the following two lemmas.

Lemma 1. *Let* $\widehat{\mathcal{P}}(X^j_t)$ *denote the estimated condition set of Algorithm S1 for* $X^j \in \mathbf{X}$ *and let* $\mathcal{P}(X^j_t)$ *denote the true parents. Assuming Causal Sufficiency, Faithfulness and the Causal Markov Condition together with a consistent conditional independence test, we have that*

$$\mathcal{P}(X^j_t) \subseteq \widehat{\mathcal{P}}(X^j_t) \quad \forall j \in \{1, \ldots, N\} \tag{S15}$$

that is, the estimated parents are a superset of the true parents.

Proof. Suppose $X^i_{t-\tau} \notin \widehat{\mathcal{P}}(X^j_t)$. Causal Sufficiency implies that $X^i_{t-\tau}$ was observed and independence can be tested. For $p_{\max} = \infty$ (default parameter value in PC_1) and with a consistent conditional independence test, PC_1 removes $X^i_{t-\tau}$ from $\widehat{\mathcal{P}}(X^j_t)$ if and only if $X^i_{t-\tau} \perp\!\!\!\perp X^j_t | \widehat{\mathcal{P}}(X^j_t) \setminus \{X^i_{t-\tau}\}$ in the last iteration of PC_1. Now Faithfulness implies that then $X^i_{t-\tau} \not\to X^j_t$ and hence $X^i_{t-\tau} \notin \mathcal{P}(X^j_t)$. □

In Lemma 2 we prove that PC_1 estimates exactly the true parents.

Lemma 2. *Let $\widehat{\mathcal{P}}(X^j_t)$ denote the estimated condition set of Algorithm S1 for $X^j \in \mathbf{X}$. Assuming Faithfulness, the Causal Markov Condition, and Causal Sufficiency we have that*

$$\widehat{\mathcal{P}}(X^j_t) = \mathcal{P}(X^j_t) \quad \forall j \in \{1, \ldots, N\} \tag{S16}$$

that is, the estimated parents are the true parents.

Proof. From Lemma 1 we know that $\widehat{\mathcal{P}}(X^j_t)$ is a superset of $\mathcal{P}(X^j_t)$, so we only need to check whether *all* parents in $\widehat{\mathcal{P}}(X^j_t)$ are also in $\mathcal{P}(X^j_t)$. Assume the contrary that $X^i_{t-\tau} \in \widehat{\mathcal{P}}(X^j_t)$, but $X^i_{t-\tau} \notin \mathcal{P}(X^j_t)$. By the contraposition of Faithfulness, $X^i_{t-\tau} \in \widehat{\mathcal{P}}(X^j_t)$ implies that $X^i_{t-\tau} \not\perp\!\!\!\perp X^j_t | \widehat{\mathcal{P}}(X^j_t) \setminus \{X^i_{t-\tau}\}$. Define $W = \widehat{\mathcal{P}}(X^j_t) \setminus \{\mathcal{P}(X^j_t), X^i_{t-\tau}\}$. The Causal Markov Condition reads $W \cup X^i_{t-\tau} \perp\!\!\!\perp X^j_t | \mathcal{P}(X^j_t)$. From the weak union property of conditional independence it follows that $X^i_{t-\tau} \perp\!\!\!\perp X^j_t | \mathcal{P}(X^j_t) \cup W$, which is equivalent to $X^i_{t-\tau} \perp\!\!\!\perp X^j_t | \widehat{\mathcal{P}}(X^j_t) \setminus \{X^i_{t-\tau}\}$, contrary to the assumption. Hence $\widehat{\mathcal{P}}(X^j_t) = \mathcal{P}(X^j_t)$. □

With these two Lemmas we can prove Proposition 1.

Proof. (Proposition 1) From Lemma 2 under the assumptions of Causal Sufficiency, Faithfulness, Causal Markov Condition, and with a consistent conditional independence test the first stage of PCMCI estimates the true set of parents, that is $\widehat{\mathcal{P}}(X^j_t) = \mathcal{P}(X^j_t)$. The MCI test (Eq. (3), Algorithm S2) establishes the absence of a link, that is, $X^i_{t-\tau} \to X^j_t \notin \widehat{\mathcal{G}}$ if and only if

$$X^i_{t-\tau} \perp\!\!\!\perp X^j_t \mid \widehat{\mathcal{P}}(X^j_t) \setminus \{X^i_{t-\tau}\}, \widehat{\mathcal{P}_{p_X}}(X^i_{t-\tau}) \tag{S17}$$

$$\overset{Lemma\ 2}{\Longleftrightarrow} X^i_{t-\tau} \perp\!\!\!\perp X^j_t \mid \mathcal{P}(X^j_t) \setminus \{X^i_{t-\tau}\}, \mathcal{P}_{p_X}(X^i_{t-\tau}) \tag{S18}$$

We need to prove

$$1) \quad X^i_{t-\tau} \to X^j_t \notin \mathcal{G} \quad \Longrightarrow \quad X^i_{t-\tau} \to X^j_t \notin \widehat{\mathcal{G}} \tag{S19}$$

$$2) \quad X^i_{t-\tau} \to X^j_t \in \mathcal{G} \quad \Longrightarrow \quad X^i_{t-\tau} \to X^j_t \in \widehat{\mathcal{G}} \tag{S20}$$

Let $X = X^i_{t-\tau}$, $Y = X^j_t$, $\mathcal{P}^*_X = \mathcal{P}^*_{p_X}(X^i_{t-\tau}) = \mathcal{P}_{p_X}(X^i_{t-\tau}) \setminus \mathcal{P}(X^j_t)$, $\mathcal{P}_Y = \mathcal{P}(X^j_t)$, $\mathcal{P}^*_Y = \mathcal{P}(X^j_t) \setminus \{X^i_{t-\tau}\}$, and $\mathcal{R} = \mathbf{X}^-_t \setminus \{X_{t-\tau}, \mathcal{P}(X^j_t), \mathcal{P}_{p_X}(X^i_{t-\tau})\}$ for notational simplicity (see Supplementary Fig. S1). In addition to the standard assumptions of causal discovery, we will make use of the basic properties of conditional independence: Decomposition, weak union, and contraction, as well as their contrapositions (*68*).

Ad 1)

$$X \to Y \notin \mathcal{G} \ \text{ and } \ (\mathcal{P}^*_X, \mathcal{R}) \cap \mathcal{P}_Y = \emptyset \ \overset{\text{Markov}}{\Longrightarrow} \ X, \mathcal{P}^*_X, \mathcal{R} \perp\!\!\!\perp Y \mid \mathcal{P}_Y \ \overset{\text{Decomposition}}{\Longrightarrow} \ X\mathcal{P}^*_X \perp\!\!\!\perp Y \mid \mathcal{P}_Y \tag{S21}$$

$$\overset{\text{Weak union}}{\Longrightarrow} \ X \perp\!\!\!\perp Y \mid \mathcal{P}_Y, \mathcal{P}^*_X \tag{S22}$$

From Lemma 2 it now follows that

$$X \perp\!\!\!\perp Y \mid \mathcal{P}_Y, \mathcal{P}^*_X \overset{\text{Lemma 2}}{\Longrightarrow} X \perp\!\!\!\perp Y \mid \widehat{\mathcal{P}}_Y, \widehat{\mathcal{P}}^*_X \overset{\text{Eq. (3)}}{\Longrightarrow} X^i_{t-\tau} \to X^j_t \notin \widehat{\mathcal{G}} \tag{S23}$$

which proves the first part.
Ad 2)

$$X \to Y \in \mathcal{G} \overset{\text{Eq. S2}}{\Longrightarrow} X \not\perp\!\!\!\perp Y \mid \mathcal{P}^*_Y, \mathcal{P}^*_X, \mathcal{R} \overset{\text{Contraposition of weak union}}{\Longrightarrow} X, \mathcal{R} \not\perp\!\!\!\perp Y \mid \mathcal{P}^*_Y, \mathcal{P}^*_X \tag{S24}$$

Now the contraposition of contraction implies that

$$X, \mathcal{R} \not\perp\!\!\!\perp Y \mid \mathcal{P}^*_Y, \mathcal{P}^*_X \implies \text{either} \quad X \not\perp\!\!\!\perp Y \mid \mathcal{P}^*_Y, \mathcal{P}^*_X \quad \text{or} \quad \mathcal{R} \not\perp\!\!\!\perp Y \mid \mathcal{P}^*_Y, \mathcal{P}^*_X, X \tag{S25}$$

But the latter of these independence relations cannot hold since we assume the Causal Markov Condition which implies

$$X, \mathcal{P}^*_X, \mathcal{R} \perp\!\!\!\perp Y \mid \mathcal{P}_Y \overset{\text{weak union}}{\Longrightarrow} \mathcal{R} \perp\!\!\!\perp Y \mid \mathcal{P}^*_Y, \mathcal{P}^*_X, X \tag{S26}$$

Hence

$$X \not\perp\!\!\!\perp Y \mid \mathcal{P}^*_Y, \mathcal{P}^*_X \overset{\text{Lemma 2}}{\Longrightarrow} X \not\perp\!\!\!\perp Y \mid \widehat{\mathcal{P}}^*_Y, \widehat{\mathcal{P}}^*_X \overset{\text{Eq. (3)}}{\Longrightarrow} X^i_{t-\tau} \to X^j_t \in \widehat{\mathcal{G}} \tag{S27}$$

which proves the second part. □

S5.3 False positive control

For finite samples, PCMCI will correctly control false positives at the specified significance level provided that (1) the condition-selection stage PC_1 detects in particular those parents that are necessary to identify conditional independencies, and (2) the MCI test is correctly calibrated. In the following two sections we discuss the effect of condition-selection and autocorrelation as a main factor for calibration.

S5.3.1 Condition-selection stage

As mentioned regarding uniform consistency above, it is difficult to derive theoretical guarantees for the first point, but empirically we found that PC_1 detects a superset of the parents with high probability. The detection performance of the PC_1 stage is shown for different time series lengths, networks sizes, and network densities in Supplementary Figs. S4,S5,S6,S7. Depending on the time series length, even for $N = 100$ still between 80% and 90% of the true parents are detected (black lines). Note that true positives refer to the detection rate for *all* parents here, cross-links as well as auto-links. In all these PC_1 implementations, the hyperparameter α_{PC} was chosen among a range of values based on AIC (see discussion in Materials and Methods section "Choice of α_{PC}"), which empirically yielded a high detection rate. Clearly, a too small α_{PC} will increase the risk of missing a parent. A missing condition in $\widehat{\mathcal{P}}(X^j_t)$ can potentially lead to a false positive in the MCI stage, but it seems that, empirically, those conditions that were missed are weak drivers and not very likely to induce spurious links. As mentioned, PC_1 is tuned to high power and for $N = 100$ also more than 80% of the identified conditions are false (red line), but still the number of conditions is only a small fraction (grey line) of the conditions used for FullCI.

As for the second point, the calibration of the MCI test will depend on the correct specification of the degrees of freedom, next to the parametric assumptions of the test. The degrees of freedom are reduced mainly by autocorrelation effects (discussed in next section), but may also be reduced due to the condition-selection stage. In the present implementation we do not take the latter factor into account. We have not found any sign of inflated false positives in our numerical experiments. Note that the PC_1 condition-selection does *not* decide *which* links are tested in the MCI stage (*all* are tested), only on the conditioning sets used. However, in order to ensure a more conservative false positive control regarding this aspect, one could split the dataset and conduct the PC_1 condition-selection on a separate part of the dataset than that used for the MCI tests. This would, however, clearly lead to a decrease in power and carry the problem of choosing the split in time series.

S5.3.2 Autocorrelation effects

The consistency proof does not require that the MCI test also conditions on the lagged parents $\mathcal{P}(X^i_{t-\tau})$, conditioning on $\mathcal{P}(X^j_t)$ suffices. We condition on the parents of the lagged variable since, for finite sample sizes, this approach helps to account for autocorrelation. Another reason, discussed in Supplementary Sect. S5.5 below, is that then the MCI test statistic value can be interpreted as a notion of *causal strength*, which allows to rank causal links in large-scale studies in a meaningful way. In the following, we provide a mathematical intuition.

Conditional independence testing requires access to the *null distribution* of the test statistic under the null hypothesis of conditional independence. As described in Supplementary Tab. S1, for the conditional independence tests considered in this paper, the null distribution is either analytically given (ParCorr), pre-computed in advance (GPDC), or generated via a local permutation test (CMI). All three methods assume that the data for a particular test $X \perp\!\!\!\perp Y \mid \mathbf{Z}$ is independent and identically distributed (*iid*). Consider the simple two-variable model

$$\begin{aligned} X_t &= aX_{t-1} + \eta^X_t \\ Y_t &= bY_{t-1} + cX_{t-1} + \eta^Y_t \end{aligned} \tag{S28}$$

where $\eta^{X,Y}$ are *iid*. For $c = 0$ we have (unconditional) independence between X and Y. But the Pearson correlation test statistic $\widehat{\rho}(X, Y)$ for this case is not distributed according to a t-distribution with $n-2$ degrees of freedom (Supplementary Tab. S1). In fact, due to the autocorrelation between samples for $a, b > 0$, the unknown true distribution has fewer degrees of freedom and will be typically wider than the assumed null distribution, leading to more false positives. The same holds for the pre-computed distribution for the distance correlation or the permutation-based distribution for mutual information.

Now consider the idea to exclude autocorrelation in Y, which is the idea behind bivariate Granger causality and the information-theoretic transfer entropy (TE) (*67*), using the measure $\rho(X_{t-\tau}; Y_t|Y_{t-1})$. For the above model for $c = 0$, this partial correlation can be simplified to $\rho(X_{t-\tau}; Y_t|Y_{t-1}) = \rho(X_{t-\tau}; \eta^Y_t|Y_{t-1})$ (*39*). η^Y is *iid*, but $X_{t-\tau}$ is not for $a > 0$ and a test would still lead to false positives as analyzed further in ref. (*34*). The conditioning of the standalone PC algorithm also is based only on the parents of Y and, hence, does not control false positives correctly for large autocorrelation in X (see Fig. 5C).

Typical remedies to account for autocorrelation are to adjust the degrees of freedom in some way, using pre-whitening, or by block-shuffling. While these approaches help to some extent for the simple bivariate case, they fail in the multivariate case that is relevant for causal discovery (*34*).

Now consider the MCI test for this example, which, in the ParCorr implementation, can be simplified

as

$$\rho^{\mathrm{MCI}}_{X\to Y}(\tau) = \rho\left(X_{t-\tau}; Y_t | \mathcal{P}(X_{t-\tau}), \mathcal{P}(Y_t) \setminus \{X_{t-\tau}\}\right) \tag{S29}$$
$$= \rho\left(aX_{t-\tau-1} + \eta^X_{t-\tau}; bY_{t-1} + \eta^Y_t \mid Y_{t-1}, X_{t-\tau-1}\right) \tag{S30}$$
$$= \rho\left(\eta^X_{t-\tau}; \eta^Y_t \mid Y_{t-1}, X_{t-\tau-1}\right) \tag{S31}$$
$$= \rho\left(\eta^X_{t-\tau}; \eta^Y_t\right) \tag{S32}$$

Thus, the final Pearson correlation test on the residuals, after regressing out the conditions, only depends on the noise terms and these are *iid*. Therefore, the analytical null distribution for $n-2-2$ degrees of freedom is appropriate here and yields expected false positive rates. A similar reasoning holds for GPDC where also nonlinear auto-dependencies are regressed out.
This case can be generalized to nonlinear additive models as discussed in Refs. (*39, 55*), here we briefly summarize this result in an information-theoretic framework based on the conditional mutual information [Eq. (S10)]

Proposition 2. *(MCI iid-ness) Assume a model with no link between variables $X_{t-\tau} \in \mathbf{X}^-_t$ and $Y_t \in \mathbf{X}_t$*

$$X_{t-\tau} = g_X\left(\mathcal{P}(X_{t-\tau})\right) + \eta^X_{t-\tau}$$
$$Y_t = g_Y\left(\mathcal{P}(Y_t)\right) + \eta^Y_t \tag{S33}$$

where g_X and g_Y are arbitrarily linear or nonlinear deterministic functions of the parents $\mathcal{P}(X_{t-\tau}), \mathcal{P}(Y_t) \in \mathbf{X}^-_t$, the noise terms $\eta^X_{t-\tau}, \eta^Y_t$ are iid*, and we assume*

$$\eta^Y_t, \eta^X_{t-\tau} \perp\!\!\!\perp \mathcal{P}(X_{t-\tau}), \mathcal{P}(Y_t) \tag{S34}$$

Then

$$I^{\mathrm{MCI}}_{X\to Y}(\tau) = I\left(\eta^X_{t-\tau}; \eta^Y_t\right) \tag{S35}$$
$$= 0 \tag{S36}$$

Proof.

$$I^{\mathrm{MCI}}_{X\to Y}(\tau) = I\left(X_{t-\tau}; Y_t | \mathcal{P}(X_{t-\tau}), \mathcal{P}(Y_t)\right) \tag{S37}$$
$$= I\left(g_X\left(\mathcal{P}(X_{t-\tau})\right) + \eta^X_{t-\tau}; g_Y\left(\mathcal{P}(Y_t)\right) + \eta^Y_t | \mathcal{P}(X_{t-\tau}), \mathcal{P}(Y_t)\right) \tag{S38}$$
$$= I\left(\eta^X_{t-\tau}; \eta^Y_t | \mathcal{P}(X_{t-\tau}), \mathcal{P}(Y_t)\right) \tag{S39}$$
$$= I\left(\eta^X_{t-\tau}; \eta^Y_t\right) = 0 \tag{S40}$$

where Eq. (S39) follows from translational invariance of CMI (*68*) and Eq. (S40) from the independence of the noise terms [Eq. (S34)]. □

Importantly, the innovation terms $\eta^X_{t-\tau}, \eta^Y_t$ are *iid*. Then the dependence of MCI only on these innovation terms implies that statistical tests on $I^{\mathrm{MCI}}_{X\to Y}(\tau) = 0$ can be conducted under the *iid-assumption* and the null distribution assumptions discussed above are appropriate, yielding well-calibrated tests. Assumption (S34) is further discussed in ref. (*39*).
Note, however, that this result is derived here for the population version of MCI and its application to empirical estimators should be considered with some caution and would rely on consistency and unbiasedness of these estimators, e.g., linear regression in ParCorr and GP in GPDC. The consistency

properties of GP regression for specific classes of functions have been studied in ref. (*69*). A full analysis of GPDC would require considering those learning theoretic guarantees on regression functions and how they impact the properties of the subsequent distance correlation independence test, which is beyond the scope of this work. For CMI no finite sample consistency results are available (*44*).

Our numerical experiments show that the MCI test largely has the expected rate of false positives even for strongly autocorrelated and nonlinear dependencies. This approach to avoiding time-dependence in the sample we found to outperform other remedies such as pre-whitening or block-shuffling (*34*).

Also the FullCI test is essentially performed on independent samples since the condition on $\mathbf{X}_t^- \setminus \{X_{t-\tau}\}$ removes any dependence with the past. However, in the GPDC implementation, we found inflated false positives (Fig. 5D), which is likely due to high dimensionality, where the autocorrelations are not properly regressed out.

S5.4 Effect size and FullCI

Now we turn to the dependent case where there *is* a causal link and the detection power depends on dimensionality and effect size, assuming sample size and significance level are fixed. Next to the lower dimensionality of the MCI test compared to FullCI, one can prove that the MCI test statistic generally has a larger or equal effect size compared to FullCI. Let I denote conditional mutual information as a general measure of dependence.

Proposition 3. *(MCI is larger or equal than FullCI) With FullCI defined as a conditional mutual information corresponding to Eq.* (S3) *it holds that*

$$I_{X\to Y}^{\mathrm{FullCI}}(\tau) \leq I_{X\to Y}^{\mathrm{MCI}}(\tau) \tag{S41}$$

Proof. To simplify notation (see Supplementary Fig. S1), denote $X = X_{t-\tau}$, $Y = Y_t$, $\mathcal{P}_X = \mathcal{P}_{p_X}(X^i_{t-\tau})$, $\mathcal{P}^*_Y = \mathcal{P}(X^j_t) \setminus \{X^i_{t-\tau}\}$, and $\mathcal{R} = \mathbf{X}_t^- \setminus \mathcal{P}^*_Y, \mathcal{P}_X$. Thus, $\mathcal{R}$ denotes the additional conditions of FullCI compared to MCI. Note that these are independent of Y given $(\mathcal{P}^*_Y, \mathcal{P}_X, X)$, because $\{\mathcal{P}^*_Y, \mathcal{P}_X\} \cup X = \{\mathcal{P}(Y_t) \cup \mathcal{P}(X_{t-\tau})\} \setminus \{X_{t-\tau}\} \cup X_{t-\tau} = \mathcal{P}(Y_t) \cup \mathcal{P}(X_{t-\tau})$ contains all of Y's parents and by the Markov assumption $I(\mathcal{R}; Y | \mathcal{P}^*_Y, \mathcal{P}_X, X) = 0$. Now consider the following two possibilities for decomposing a multivariate mutual information using the chain rule

$$I((X,\mathcal{R}); Y | \mathcal{P}^*_Y, \mathcal{P}_X) = \underbrace{I(X; Y | \mathcal{P}^*_Y, \mathcal{P}_X)}_{\mathrm{MCI}} + \underbrace{I(\mathcal{R}; Y | \mathcal{P}^*_Y, \mathcal{P}_X, X)}_{=0} \tag{S42}$$

$$= \underbrace{I(\mathcal{R}; Y | \mathcal{P}^*_Y, \mathcal{P}_X)}_{\geq 0} + \underbrace{I(X; Y | \mathcal{P}^*_Y, \mathcal{P}_X, \mathcal{R})}_{\mathrm{FullCI}} \tag{S43}$$

$$\Longrightarrow I_{X\to Y}^{\mathrm{MCI}}(\tau) = I(X; Y | \mathcal{P}^*_Y, \mathcal{P}_X) \geq I(X; Y | \mathcal{P}^*_Y, \mathcal{P}_X, \mathcal{R}) = I_{X\to Y}^{\mathrm{FullCI}}(\tau) \tag{S44}$$

□

FullCI and MCI are equal if the additional conditioning variables $\mathcal{R}$ are independent of Y given $\{\mathcal{P}^*_Y, \mathcal{P}_X\}$. If they are not, for example, if variables in $\mathcal{R}$ are causally affected by X (i.e., they are causal descendents of X), then conditioning on them will generally lead to a lower effect size. Hence, the variables $\mathcal{R}$ are irrelevant in that they are not required as a separating set to establish that X and Y are independent (if this is the case) and, further, increase the risk to reduce effect size. Both the lower dimensionality and higher effect size are responsible for the empirically found higher power of the MCI test compared to FullCI (or Granger causality).

S5.5 Causal strength

MCI's effect size is not only always larger (or equal) than FullCI, but also can be interpreted as a measure of causal strength. Here, we employ an information-theoretic concept, based on a number of desirable dependence measure properties (*39*).

Consider model (S33) with an added dependency term of Y on X

$$\begin{aligned} X_{t-\tau} &= g_X\left(\mathcal{P}\left(X_{t-\tau}\right)\right) + \eta^X_{t-\tau} \\ Y_t &= g_Y\left(\mathcal{P}\left(Y_t\right) \setminus \{X_{t-\tau}\}\right) + f\left(X_{t-\tau}\right) + \eta^Y_t \end{aligned} \tag{S45}$$

We now investigate an information-theoretic definition of causal strength based on conditional mutual information:

$$I\left(\eta^X_{t-\tau}; f\left(X_{t-\tau}\right) + \eta^Y_t \mid \mathcal{P}\left(X_{t-\tau}\right)\right) \tag{S46}$$

If we had experimental access for intervening in $\eta^X_{t-\tau}$ at a particular time $t-\tau$, then this notion of causal strength information-theoretically quantifies how much of this momentary perturbation can be detected in Y_t, excluding information contained in the past. This measure directly corresponds to "momentary" dependence in Y_t on $X_{t-\tau}$ that does not come through the parents of $X_{t-\tau}$. There are several information-theoretic proposals for measures of causal strength, see, for example, ref. (*57*). Our definition of causal strength is based on the fundamental concept of *source entropy* as further discussed in ref. (*39*).

MCI for this model is an estimator of causal strength since, similar to the above proof

$$I^{\text{MCI}}_{X\to Y}(\tau) = I\left(X_{t-\tau}; Y_t | \mathcal{P}\left(X_{t-\tau}\right), \mathcal{P}\left(Y_t\right) \setminus \{X_{t-\tau}\}\right) \tag{S47}$$

$$= I\left(g_X\left(\mathcal{P}\left(X_{t-\tau}\right)\right) + \eta^X_{t-\tau}; g_Y\left(\mathcal{P}\left(Y_t\right) \setminus \{X_{t-\tau}\}\right) + f\left(X_{t-\tau}\right) + \eta^Y_t | \mathcal{P}\left(X_{t-\tau}\right), \mathcal{P}\left(Y_t\right) \setminus \{X_{t-\tau}\}\right) \tag{S48}$$

$$= I\left(\eta^X_{t-\tau}; f\left(X_{t-\tau}\right) + \eta^Y_t | \mathcal{P}\left(X_{t-\tau}\right), \mathcal{P}\left(Y_t\right) \setminus \{X_{t-\tau}\}\right) \tag{S49}$$

$$= I\left(\eta^X_{t-\tau}; f\left(X_{t-\tau}\right) + \eta^Y_t | \mathcal{P}\left(X_{t-\tau}\right)\right) \tag{S50}$$

For a linear dependence $f\left(X_{t-\tau}\right) = cX_{t-\tau}$, MCI can be further simplified

$$I^{\text{MCI}}_{X\to Y}(\tau) = I\left(\eta^X_{t-\tau}; c\eta^X_{t-\tau} + \eta^Y_t\right) \tag{S51}$$

which for partial correlation in the Gaussian case becomes

$$\rho^{\text{MCI}}_{X\to Y} = \frac{c\sigma_X}{\sqrt{\sigma^2_Y + c^2\sigma^2_X}} \tag{S52}$$

where $\sigma^2_\cdot$ now denotes the variances of the noise terms η. Thus, for a linear additive dependency, where causal strength can be attributed to a single coefficient c, MCI depends only on this coefficient and on the noise terms, but not on g_Y, g_X. MCI is then independent of dependencies due to the parents $\mathcal{P}\left(X_{t-\tau}\right)$ and $\mathcal{P}\left(Y_t\right)$, which could include auto-dependencies. A causal signal can, thus, be better detected against noise coming from confounding drivers or autocorrelation. This theoretical result is confirmed in the numerical experiments in Fig. 6. PCMCI results in a confined scaling of power with link strength. In sum, larger effect size and lower dimensionality lead to higher detection power of PCMCI compared to FullCI.

Correlation can be very different from the causal effect, that is, from the link coefficient in a linear model. Take the following example

$$
\begin{aligned}
Z_t &= \eta_t^Z \\
X_t &= aZ_{t-1} + \eta_t^X \\
Y_t &= bZ_{t-2} + cX_{t-1} + \eta_t^X
\end{aligned} \tag{S53}
$$

Here the correlation for the link $X_{t-1} \to Y_t$ is

$$
\rho(X_{t-1}, Y_t) = \frac{c\Gamma_X + ab\Gamma_Z}{\sqrt{\Gamma_X}\sqrt{\Gamma_Y}} \tag{S54}
$$

where $\Gamma_\cdot$ denotes the variances. The correlation, thus, depends not only on c, and may even become zero depending on a and b. The MCI partial correlation, on the other hand, estimates the causal strength given by $\frac{c\sigma_X}{\sqrt{\sigma_Y^2 + c^2\sigma_X^2}}$ as derived above.

On the other hand, for nonlinear cases, there can still be various dependencies, because the function f mixes $\eta^X_{t-\tau}$ with $\mathcal{P}(X_{t-\tau})$ (*39*). As for the consistency proof given above, the results here are only derived for the population version of MCI.

Section S6. Numerical experiments

S6.1 Model setup

To evaluate and compare different causal discovery methods, we use a model that mimics the properties of real world data. Here we model six of the major challenges of time series from complex systems: High-dimensionality, nonlinearity, strong autocorrelation, time lagged causal dependencies, observational noise, and non-stationarity. Consider the following model from which we generate 20 ensemble members per number of variables N, number of links L, and coupling strength c: For $i, j \in \{1, ..., N\}$ we randomly choose L links "$i \to j$" with $i \neq j$ and generate time series according to

$$
X_t^j = a_j X_{t-1}^j + c \sum_i f_i(X^i_{t-\tau_i}) + \eta_t^j \tag{S55}
$$

for $j \in \{1, \ldots, N\}$, and where (1) a_j are uniformly randomly drawn from $\{0, 0.2, 0.4, 0.6, 0.8, 0.9\}$ for one half of the ensemble and from $\{0.6, 0.8, 0.9, 0.95\}$ for another, more autocorrelated, half of the 20 network ensemble members, except for the high-density experiments where a_j are only drawn from $\{0, 0.2, 0.4, 0.6, 0.8, 0.9\}$. (2) $\eta^j \sim \mathcal{N}(0,1)$ is *iid* Gaussian noise. (3) For ParCorr experiments: $f_i(x) = f^{(1)}(x) = x$; for nonlinear model experiments, among the L links in each network 50% are linear functions $f^{(1)}(x) = x$, 25% are nonlinear $f^{(2)}(x) = (1 - 4e^{-x^2/2})x$ and 25% are nonlinear $f^{(3)}(x) = (1 - 4x^3 e^{-x^2/2})x$. (4) τ_i is uniformly randomly drawn from $\{1, 2\}$. (5) c is constant for all links in a model and its absolute value differs among the experiments (see descriptions in Supplementary Tab. S2); the sign of c is positive or negative with equal probability. To guarantee stationarity, the functions $f_i(x)$ are all linear in the limit of large x and we dismiss all models for which the corresponding vector autoregressive model with nonlinear functions f_i replaced by linear ones is nonstationary according to a unit root test (*70*). Figure 5B gives an example realization. With $L = N$ links in each model, we have an average cross-in-degree of 1 for all network sizes (plus an auto-dependency). The cross-link density, on the other hand, decays with N as $\frac{N}{N(N-1)\tau_{\max}} = \frac{1}{(N-1)\tau_{\max}}$. We also tested networks with $L = 2N$ links, but we note

that it is difficult to generate stationary time series for such densely connected processes. The effect of observational noise is evaluated by adding Gaussian noise $\mathcal{N}(0, \sigma^2)$ with different standard deviations σ. Non-stationarity is modeled by adding a sinusoidal dependence $a \sin(2\pi t/25)$ with different amplitudes a. The causality benchmark platform `www.causeme.net` contains these and further datasets to facilitate method evaluation.

S6.2 Performance evaluation

To assess false positive rates and true positive rates for the individual links in each model, 100 time series realizations were generated for each model. Note that the error in the estimate of a false positive rate of 0.05 (or a true positive rate of 0.95) is roughly $\sqrt{0.05(1-0.05)/100} \approx 0.02$. The bottom rows in most figures show boxplots of the distribution of false positive rate and the upper row(s) of the true positive rates for linear (and nonlinear) dependencies. Only cross-links were considered here. As illustrated in Fig. 5A, the left and right boxplots in the figures depict the distributions for all weakly autocorrelated pairs with mean autocorrelation $(\rho(X_{t-1}, X_t) + \rho(Y_{t-1}, Y_t))/2 < 0.7$ among the two variables X and Y of a link, and for strongly autocorrelated pairs $((\rho(X_{t-1}, X_t) + \rho(Y_{t-1}, Y_t))/2 \geq 0.7)$, respectively. The boxes show the 25-75% and whiskers the 1-99% percentile range, the median is marked by a bar and the mean with 'x'. Note the logarithmic y-axis in the bottom panel for false positive rates > 0.1. The tick labels on the top of the figures note the average runtime and its standard deviation across the different model setups. The runtime estimates were evaluated on Intel Xeon E5-2667 v3 8C processors with 3.2GHz. These runtimes will depend on implementation. An overview of runtimes for the experiments is shown in Supplementary Fig. S16. In Supplementary Tab. S2 we list the model setups for the numerical experiments. Supplementary Tab. S3 gives details on the compared methods. The experiments were evaluated on a high-performance cluster.

S6.3 AN(C)OVA analysis

In Supplementary Fig. S17 and Tabs. S4–S12 we show results of an analysis of variance (ANOVA) and covariance (ANCOVA) of the numerical experiments. Supplementary Tab. S2 lists the ANOVA results tables corresponding the the different experiments. We use the `statsmodels` package and estimate the ANOVA model $R \sim C(W)$ where R is the true positive rate, the false positive rate, or the runtime, and $W = N$, T, σ, a in the different experiment analyses and C indicates that we treat this as a categorical variable. Every ANOVA analysis yields coefficients for the change of the mean of the response variable, e.g., detection power, for every level of the independent variable, e.g., N. With $N = 2$ as a reference level (intercept), this results in M coefficients Δ_i for the change of detection power for $N_i = 5, 10, 20, 40, 60, 80, 100$ with respect to $N = 2$. To summarize these results, in Supplementary Tabs. S4–S8 we depict $R \pm \bar{\Delta}$ where R is the reference level and $\bar{\Delta} = \frac{1}{M}\sum_{i=1}^{M} \frac{\Delta_i}{W_i - W}$ gives the average relative change of R per one unit increase in W (or rescaled for better readability). We mark $\bar{\Delta}$ in bold if at least one coefficient Δ_i is significantly different from zero at a 1% significance level. Furthermore, we study the ANCOVA model $R \sim C(N) + C(T) + C(N) * C(T)$ on the experiment named ANCOVA in Supplementary Tab. S2 to also study interaction effects between N and T, that is, how the dependence of R on N changes for different values of T, and vice versa. In Supplementary Tabs. S9–S12 we show all corresponding coefficients and further statistics. An entry "C(N)[T.40]" denotes the ANOVA coefficient corresponding to $N = 40$ (T stands for treatment in statsmodel output) and "C(N)[T.40]:C(T)[T.300]" an interaction coefficient. Supplementary Figure S17 depicts the true positive (top row) and false positive rates (bottom row) for different N (x-axis) and different T (colored lines) for different methods.

S6.4 Chaotic coupled logistic maps

In Supplementary Fig. S18 we depict a comparison of PCMCI in the CMI implementation with the nonlinear state-space method convergent cross-mapping (CCM) (*14*), see Supplementary Sect. S2.4. As in ref. (*34*), we consider three coupled logistic maps with optionally added dynamical noise and here additionally investigate the effect of strong common driver coupling

$$\begin{aligned} Z_t &= Z_{t-1}(r - rZ_{t-1} + \sigma\eta_t^Z) \mod 1 \\ X_t &= X_{t-1}(r - rX_{t-1} - cZ_{t-1} + \sigma\eta_t^X) \mod 1 \\ Y_t &= Y_{t-1}(r - rY_{t-1} - cZ_{t-1} + \sigma\eta_t^Y) \mod 1 \end{aligned} \tag{S56}$$

with uniformly distributed independent noise η and $r = 4$ leading to chaotic dynamics. Here the coupling coefficient c controls the degree of common driving and σ the amount of dynamical noise in the system. To evaluate true and false positive rates, 200 realizations with time series length $T = 150, 300$ were generated. All methods were evaluated at a significance level of 5%. PCMCI was estimated with $\alpha_{\mathrm{PC}} = 0.1$, $\tau_{\min} = 0$, $\tau_{\max} = 2$ and CMIknn parameters $k_{CMI} = 0.1n$, $k_{perm} = 5$ and $B = 500$ permutation surrogates (*44*), where n is the sample size.

S7 Supplementary Algorithms

Algorithm S1. Pseudo-code for condition-selection algorithm $\mathrm{PC}_{q_{\max}}$ to estimate parents of X^j_t. We use this algorithm as a pre-selection stage in PCMCI with $p_{\max} = N\tau_{\max}$ (i.e., no restriction on the maximum number of parents) and $q_{\max} = 1$. For the standalone PC-stable algorithm, we set $q_{\max}$ to a large value of 10.

Require: Time series dataset $\mathbf{X} = (X^1, X^2, \ldots, X^N)$, selected variable X^j, maximum time lag $\tau_{\max}$, significance threshold α_{PC}, maximum condition dimension $p_{\max}$ (default $p_{\max} = N\tau_{\max}$), maximum number of combinations $q_{\max}$ (default $q_{\max} = 1$), conditional independence test function

function $\mathrm{CI}(X, Y, \mathbf{Z})$
    Test $X \perp\!\!\!\perp Y \mid \mathbf{Z}$ using test statistic measure I
    return p-value, test statistic value I
Initialize preliminary set of parents $\widehat{\mathcal{P}}(X^j_t) = \{X^i_{t-\tau} : i \in \{1, \ldots, N\},\ \ \tau \in \{1, \ldots, \tau_{\max}\}\}$
Initialize dictionary of test statistic values $I^{\min}(X^i_{t-\tau} \to X^j_t) = \infty\ \ \forall\ X^i_{t-\tau} \in \widehat{\mathcal{P}}(X^j_t)$
for $p = 0, \ldots, p_{\max}$ **do**
    if $|\widehat{\mathcal{P}}(X^j_t)| - 1 < p$ **then**
        Break for-loop
    for all $X^i_{t-\tau}$ in $\widehat{\mathcal{P}}(X^j_t)$ **do**
        $q = -1$
        for all lexicographically chosen subsets $\mathcal{S} \subseteq \widehat{\mathcal{P}}(X^j_t) \setminus \{X^i_{t-\tau}\}$ with $|\mathcal{S}| = p$ **do**
            $q = q + 1$
            if $q \geq q_{\max}$ **then**
                Break from inner for-loop
            Run CI test to obtain (p-value, I) $\leftarrow \mathrm{CI}(X^i_{t-\tau}, X^j_t, \mathcal{S})$
            if $|I| < I^{\min}(X^i_{t-\tau} \to X^j_t)$ **then** ▷ Store min. I of parent among all tests
                $I^{\min}(X^i_{t-\tau} \to X^j_t) = |I|$
            if p-value $> \alpha_{\mathrm{PC}}$ **then** ▷ Removed only after all $X^i_{t-\tau}$ have been tested
                Mark $X^i_{t-\tau}$ for removal from $\widehat{\mathcal{P}}(X^j_t)$
                Break from inner for-loop
    Remove non-significant parents from $\widehat{\mathcal{P}}(X^j_t)$
    Sort parents in $\widehat{\mathcal{P}}(X^j_t)$ by $I^{\min}(X^i_{t-\tau} \to X^j_t)$ from largest to smallest
return $\widehat{\mathcal{P}}(X^j_t)$

Algorithm S2. Pseudo-code for MCI causal discovery stage. Here we state the algorithm for $\tau \geq 0$, then causal links for $\tau = 0$ correspond to contemporaneous links, which are left undirected here.

Require: Time series dataset $\mathbf{X} = (X^1, X^2, \ldots, X^N)$, sorted parents $\widehat{\mathcal{P}}(X_t^j)$ for all variables X^j estimated with Algorithm S1, maximum time lag $\tau_{\max}$, maximum number p_X of parents of variable X^i, and conditional independence test function CI

1: **for all** $(X_{t-\tau}^i, X_t^j)$ with $i, j \in \{1, \ldots, N\}$, $\tau \in \{0, \ldots, \tau_{\max}\}$, excluding (X_t^j, X_t^j) **do**

2: Remove $X_{t-\tau}^i$ from $\widehat{\mathcal{P}}(X_t^j)$ if necessary

3: Define $\widehat{\mathcal{P}}_{p_X}(X_{t-\tau}^i)$ as the first p_X parents from $\widehat{\mathcal{P}}(X_t^i)$, shifted by τ

4: Run MCI test to obtain (p-value, I) $\leftarrow$ CI$(X_{t-\tau}^i, X_t^j, \mathbf{Z} = \{\widehat{\mathcal{P}}(X_t^j), \widehat{\mathcal{P}}_{p_X}(X_{t-\tau}^i)\})$

5: Optionally adjust p-values of all links by False Discovery Rate-approach (FDR)

6: **return** p-values or q-values (for FDR-adjusted tests) and MCI test statistic values

Algorithm S3. Pseudo-code for adaptive Lasso regression for inference of non-zero coefficients and p-values for linear model $Y = \mathbf{X}\beta$. Lasso implemented in python's `sklearn` package `LassoCV` with default parameters (except `fit_intercept=False`) and with λ_n chosen by cross-validation using `TimeSeriesSplit(n_splits=5)`. In the numerical experiments we used $k_{\max} = 5$. The first part is adapted from `gist.github.com/agramfort/1610922`

Require: Data $\mathbf{x} \in \mathbb{R}^{n\times d}$, $y \in \mathbb{R}^n$, maximum number $k_{\max}$ of iterations

Standardize $\mathbf{x}$ and y

Initialize weights $w_j = 1$ for $j = 1, \ldots, d$

for $k = 1, \ldots, k_{\max}$ **do**

  Scale features $\mathbf{x}_j^* = \mathbf{x}_j / w_j$ for $j = 1, \ldots, d$

  Solve Lasso problem with λ_n chosen by time series based cross-validation

$$\beta^* = \operatorname*{argmin}_{\beta} \left\| y - \sum_{j=1}^{d} \mathbf{x}_j^* \beta_j \right\|^2 + \lambda_n \sum_{j=1}^{d} |\beta_j| \tag{S57}$$

  Re-weight coefficients $\beta_j^{**} = \beta_j^* / w_j$ for $j = 1, \ldots, d$

  Compute new weights $w_j = 1/(2|\beta_j^{**}|^{\frac{1}{2}} + \epsilon)$ for $j = 1, \ldots, d$, where ϵ is the machine limit for floats

Define active set as $\mathcal{A} = \{j : \beta_j^{**} \neq 0\}$

Solve OLS regression on active set

$$\tilde{\beta} = \operatorname*{argmin}_{\beta} \left\| y - \sum_{j \in \mathcal{A}} \mathbf{x}_j \beta_j \right\|^2 \tag{S58}$$

and record corresponding p-values $\tilde{p}_j$ for $j \in \mathcal{A}$

Define p-values

$$p_j = \begin{cases} \tilde{p}_j & \text{if } j \in \mathcal{A} \\ 1 & \text{otherwise} \end{cases} \tag{S59}$$

return p_j for $j = 1, \ldots, d$

S8 Supplementary Tables

Table S1. Overview of conditional independence tests to test whether X and Y are independent conditional on $\mathbf{Z}$. The tests are discussed in Supplementary Sect. S4. All tests assume continuously-valued data. The Gaussian process (GP) was fitted with `sklearn`'s `GaussianProcessRegressor` with `kernel=RBF()+WhiteKernel()` and `alpha=0`. The bandwidth of the Kernel in `sklearn` is estimated by maximizing marginal likelihood (ML-II). D_Z is the cardinality of $\mathbf{Z}$. Note that the conditional independence tests in the present context are conducted with a sample size of $n = T - 2\tau_{\max}$, where T is the time series length and the first $2\tau_{\max}$ samples are cut off since this is the maximum time lag of a lagged parent in $\widehat{\mathcal{P}}(X^i_{t-\tau})$. Further, we might need to account for missing and masked samples.

	ParCorr	GPDC	CMI
Assumed model	$X = \mathbf{Z}\beta_X + \epsilon^X$<br>$Y = \mathbf{Z}\beta_Y + \epsilon^Y$<br>$\epsilon^{\cdot} \sim \mathcal{N}(0, \sigma_{\cdot}^2)$	$X = h_X(\mathbf{Z}) + \epsilon^X$<br>$Y = h_Y(\mathbf{Z}) + \epsilon^Y$<br>$\epsilon^{\cdot} \sim \mathcal{N}(0, \sigma_{\cdot}^2)$	No parametric assumptions, direct estimation of CMI $I(X;Y\|\mathbf{Z})$ [Eq. (S10)]
Estimation	Get residuals from OLS fit<br>$\widehat{r_X} = X - \mathbf{Z}\widehat{\beta_X}$<br>$\widehat{r_Y} = Y - \mathbf{Z}\widehat{\beta_Y}$,<br>estimate correlation $\widehat{\rho}(\widehat{r_X}, \widehat{r_Y})$	Standardize time series,<br>fit $\widehat{h_X}$, $\widehat{h_Y}$ with GP, get residuals<br>$\widehat{r_X} = X - \widehat{h_X}(\mathbf{Z})$<br>$\widehat{r_Y} = Y - \widehat{h_Y}(\mathbf{Z})$,<br>transform $\widehat{r_X}, \widehat{r_Y}$ to uniform marginals (copula), estimate distance correlation $\widehat{\mathrm{dCor}}(\widehat{r_X}, \widehat{r_Y})$	Rank-transform time series, use nearest-neighbor estimator (*75*) [Eq. (S11)]
Parameter(s)	–	MLE estimation with Radial Basis Function+White kernel, Euclidean distance for dCor	Nearest neighbors $k_{\mathrm{CMI}} = 50$, $k_{\mathrm{perm}} = 5$
Null distribution	Analytically known,<br>$t = \widehat{\rho}(\widehat{r_X}, \widehat{r_Y})\sqrt{\frac{n-2-D_Z}{1-\widehat{\rho}(\widehat{r_X}, \widehat{r_Y})^2}}$<br>follows t-distribution with $n - 2 - D_Z$ degrees of freedom	Pre-computed for each sample size n from $\widehat{\mathrm{dCor}}(u, v)$ with $u, v \sim U(0, 1)$ (valid due to copula transform)	Local permutation test (*44*)

Table S2. Model configurations for different experiments. The model is given in Eq. (S55). For each configuration, 100 time series realizations were generated to evaluate false and true positives. The factor that is varied in each experiment (along the x-axis in the figures) is marked in bold. The coupling functions are $f^{(1)}(x) = x$, $f^{(2)}(x) = (1 - 4e^{-x^2/2})x$, $f^{(3)}(x) = (1 - 4x^3 e^{-x^2/2})x$. An overview of runtimes for the experiments is shown in Fig. S16.

Experiment	Variables N and links L	Functions f_i	Sample size T	Coefficient c	Number of random networks
High-dimensionality ParCorr Figs. 5C,S4,Tab. S4	$\mathbf{N = 2, 5, 10, 20, 40, 60, 80, 100}$ $L = N$	$f^{(1)}$	150	0.287	20 per N
High-dimensionality ParCorr Fig. S5,Tab. S4	$\mathbf{N = 2, 5, 10, 20, 40, 60, 80, 100}$ $L = N$	$f^{(1)}$	300	0.2	20 per N
High-density ParCorr Fig. S6,Tab. S5	$\mathbf{N = 10, 20, 40, 60}$ $L = 2N$	$f^{(1)}$	150	0.287	10 per N
High-density ParCorr Fig. S7,Tab. S5	$\mathbf{N = 10, 20, 40, 60}$ $L = 2N$	$f^{(1)}$	300	0.2	10 per N
Sample size ParCorr Fig. S9,Tab. S7	$N = 20,\ L = N$	$f^{(1)}$	**150**, **300**, **600**	0.2	20
ANCOVA ParCorr Fig. S17,Tabs. S9–S12	$\mathbf{N = 2, 5, 10, 20, 40, 60, 80, 100}$ $L = N$	$f^{(1)}$	**150**, **200**, **300**	0.2	20 per N
Causal effects ParCorr Fig. 6	$N = 20,\ L = N$	$f^{(1)}$	150	**0.2**, **0.247**, **0.287**, **0.324**, **0.414**	20 per c
Observational noise ParCorr Fig. S14,Tab. S8	$N = 20,\ L = N$ Noise $\mathcal{N}(0, \sigma^2)$ with $\sigma = \mathbf{0}, \mathbf{0.1}, \mathbf{0.25}, \mathbf{0.5}, \mathbf{1}, \mathbf{2}$	$f^{(1)}$	150	0.287	20
Non-stationarity ParCorr Fig. S15,Tab. S8	$N = 20,\ L = N$ Trend $a\sin(2\pi t/25)$ with $\mathbf{a} = \mathbf{0}, \mathbf{1}, \mathbf{2}, \mathbf{3}, \mathbf{5}, \mathbf{7}$	$f^{(1)}$	150	0.287	20
Parameter PC_1 α_{PC} ParCorr Fig. S8A	$N = 20,\ L = N$	$f^{(1)}$	150	0.287	20
Parameter PC_1 α_{PC} GPDC Fig. S8B	$N = 10,\ L = N$	50% $f^{(1)}$ 25% $f^{(2)}$ 25% $f^{(3)}$	250	0.287	20
Parameter PC_1 α_{PC} CMI Fig. S8C	$N = 5,\ L = N$	50% $f^{(1)}$ 25% $f^{(2)}$ 25% $f^{(3)}$	500	0.324	20
High-dimensionality GPDC Figs. 5D,S10,Tab. S6	$\mathbf{N = 2, 5, 10, 20, 40}$ $L = N$	50% $f^{(1)}$ 25% $f^{(2)}$ 25% $f^{(3)}$	250	0.287	20 per N

Sample size<br>GPDC<br>Fig. S11,Tab. S7	$N = 10,\ L = N$	50% $f^{(1)}$<br>25% $f^{(2)}$<br>25% $f^{(3)}$	**250**, **500**	0.2	20
High-dimensionality<br>CMI<br>Fig. 5E,S12,Tab. S6	$\mathbf{N = 2, 5, 10}$<br>$L = N$	50% $f^{(1)}$<br>25% $f^{(2)}$<br>25% $f^{(3)}$	500	0.324	20 per N
Sample size<br>CMI<br>Fig. S13,Tab. S7	$N = 5,\ L = N$	50% $f^{(1)}$<br>25% $f^{(2)}$<br>25% $f^{(3)}$	**500**, **1000**	0.324	20

Table S3. Overview of methods compared in numerical experiments. The methods are defined in Supplementary Sects. S2,S3 and the implementations ParCorr/GPDC/CMI are explained in Supplementary Sect. S4. All methods are run with $\tau_{\max} = 5$ except for CCM.

Acronym	Method	Details
Corr / dCor / MI	Pairwise unconditional independence tests	see Supplementary Sect. S2.5
BivCI<br>ParCorr	Bivariate conditional independence tests	see Supplementary Sect. S3.2<br>condition only on past of response variable
FullCI<br>ParCorr	Linear vector-autoregressive model	see Supplementary Sect. S2.1<br>fit with OLS in `statsmodels`
FullCI<br>GPDC / CMI	Independence test conditioning<br>on full past with GPDC or CMI approach	see Supplementary Sect. S2.1
Lasso	Adaptive Lasso regression	see Supplementary Sect. S2.2 and Algorithm S3
CCM	Convergent cross-mapping (*14*)	see Supplementary Sect. S2.4<br>embedding dimension fixed ($E = 2$)<br>or optimized
PC<br>ParCorr / GPDC / CMI	Standalone PC algorithm	see Supplementary Sect. S2.3<br>and Algorithm S1<br>$\alpha_{\mathrm{PC}} = 0.2$, $q_{\max} = 10$
PC_1<br>ParCorr	Condition-selection stage<br>as standalone	see Materials and Methods<br>and Algorithm S1<br>$\alpha_{\mathrm{PC}} = \{0.1, 0.2, 0.3, 0.4\}$ via AIC, $q_{\max} = 1$
PC_1+MCI_{all}<br>ParCorr	PCMCI with α_{PC}-optimization	see Materials and Methods<br>and Algorithms S1,S2<br>$\alpha_{\mathrm{PC}} = \{0.1, 0.2, 0.3, 0.4\}$ via AIC,<br>$q_{\max} = 1$, p_X unrestricted
PC_1^{α}+MCI_{all}<br>ParCorr / GPDC / CMI	PCMCI without α_{PC}-optimization	see Materials and Methods<br>and Algorithms S1,S2<br>$\alpha_{\mathrm{PC}} = 0.2$ (or given), $q_{\max} = 1$, p_X unrestricted
PC_1+MCI_3<br>ParCorr	PCMCI with α_{PC}-optimization<br>and truncated $\widehat{\mathcal{P}}_X$	see Materials and Methods<br>and Algorithms S1,S2<br>$\alpha_{\mathrm{PC}} = \{0.1, 0.2, 0.3, 0.4\}$ via AIC,<br>$q_{\max} = 1$, $p_X = 3$
PC_1^{α}+MCI_3<br>GPDC / CMI	PCMCI without α_{PC}-optimization<br>and truncated $\widehat{\mathcal{P}}_X$	see Materials and Methods<br>and Algorithms S1,S2<br>$\alpha_{\mathrm{PC}} = 0.2$, $q_{\max} = 1$, $p_X = 3$
PC_1+MCI_0<br>ParCorr	PCMCI with α_{PC}-optimization<br>and no condition on $\widehat{\mathcal{P}}_X$	see Supplementary Sect. S3.1<br>and Algorithms S1,S2<br>$\alpha_{\mathrm{PC}} = \{0.1, 0.2, 0.3, 0.4\}$ via AIC,<br>$q_{\max} = 1$, $p_X = 0$
PC_1+MCI_0pw<br>ParCorr	PCMCI with α_{PC}-optimization<br>and no condition on $\widehat{\mathcal{P}}_X$<br>and pre-whitening	see Supplementary Sect. S3.1<br>and Algorithms S1,S2<br>$\alpha_{\mathrm{PC}} = \{0.1, 0.2, 0.3, 0.4\}$ via AIC,<br>$q_{\max} = 1$, $p_X = 0$

Table S4. Summarized ANOVA results for high-dimensionality ParCorr experiments in Supplementary Tab. S2. See Supplementary Sect. S6.3 for an explanation.

High-dimensionality I ParCorr	Corr	BivCI	FullCI	Lasso	PC	PC_1+MCI_{all}
TPR	0.918 − 0.048	0.978 − 0.058	0.847−**0.176**	0.901−**0.062**	0.458 − 0.035	0.879−**0.027**
FPR	0.618−**0.103**	0.286−**0.077**	0.046+**0.003**	0.039−**0.018**	0.024−**0.013**	0.053 − 0.000
Runtime [s]	0.007+**0.737**	0.013+**1.483**	0.001 + 0.009	0.368+**10.156**	0.052+**6.247**	0.098+**16.316**
(at N=2 ± change per 10)						

High-dimensionality II ParCorr	PC_1+MCI_3	PC_1+MCI_0	PC_1+MCI_0pw	PC_1^α+MCI_{all}	PC_1+MCI_{all}
TPR	0.902−**0.033**	0.924−**0.010**	0.900−**0.032**	0.895−**0.029**	0.879−**0.027**
FPR	0.049+**0.002**	0.054 + 0.001	0.160−**0.056**	0.051 + 0.001	0.053 − 0.000
Runtime [s]	0.098+**15.343**	0.097+**15.171**	0.081+**22.445**	0.032+**4.925**	0.098+**16.316**
(at N=2 ± change per 10)					

High-dimensionality I ($T = 300$) ParCorr	Corr	BivCI	FullCI	Lasso	PC	PC_1+MCI_{all}
TPR	0.915 − 0.008	0.976 − 0.024	0.858−**0.073**	0.889−**0.042**	0.557 − 0.056	0.916−**0.035**
FPR	0.538−**0.052**	0.233−**0.036**	0.051−**0.002**	0.036−**0.017**	0.020−**0.010**	0.048+**0.003**
Runtime [s]	0.007+**0.762**	0.014+**1.574**	0.002+**0.038**	0.371+**19.008**	0.048+**8.373**	0.099+**22.110**
(at N=2 ± change per 10)						

High-dimensionality II ($T = 300$) ParCorr	PC_1+MCI_3	PC_1+MCI_0	PC_1+MCI_0pw	PC_1^α+MCI_{all}	PC_1+MCI_{all}
TPR	0.897−**0.008**	0.938−**0.013**	0.914−**0.021**	0.901−**0.013**	0.916−**0.035**
FPR	0.051 − 0.000	0.049+**0.001**	0.142−**0.037**	0.049 + 0.002	0.048+**0.003**
Runtime [s]	0.099+**20.102**	0.097+**19.875**	0.084+**47.705**	0.033+**5.988**	0.099+**22.110**
(at N=2 ± change per 10)					

Table S5. Summarized ANOVA results for high-density ParCorr experiments in Supplementary Tab. S2. See Supplementary Sect. S6.3 for an explanation.

High-dimensionality I ($L = 2N$) ParCorr	Corr	BivCI	FullCI	Lasso	PC	PC_1+MCI_{all}
TPR	0.822 + 0.006	0.879 − 0.001	0.733−**0.290**	0.833−**0.029**	0.525 + 0.010	0.848−**0.034**
FPR	0.352−**0.036**	0.166−**0.027**	0.049+**0.002**	0.021−**0.004**	0.006−**0.001**	0.052+**0.001**
Runtime [s]	0.162+**0.802**	0.317+**1.578**	0.004+**0.030**	3.339+**12.120**	1.209+**5.366**	2.834+**16.558**
(at N=10 ± change per 10)						

High-dimensionality II ($L = 2N$) ParCorr	PC_1+MCI_3	PC_1+MCI_0	PC_1+MCI_0pw	PC_1^α+MCI_{all}	PC_1+MCI_{all}
TPR	0.868−**0.014**	0.910−**0.010**	0.868−**0.016**	0.860−**0.022**	0.848−**0.034**
FPR	0.051+**0.001**	0.052+**0.001**	0.076−**0.003**	0.051 + 0.000	0.052+**0.001**
Runtime [s]	2.738+**15.481**	2.726+**15.286**	3.195+**23.683**	0.875+**4.940**	2.834+**16.558**
(at N=10 ± change per 10)					

High-dimensionality I ($L = 2N$, $T = 300$) ParCorr	Corr	BivCI	FullCI	Lasso	PC	PC_1+MCI_{all}
TPR	0.830 + 0.016	0.915 + 0.007	0.844−**0.112**	0.845−**0.033**	0.593+**0.017**	0.884−**0.019**
FPR	0.293−**0.000**	0.136−**0.013**	0.049+**0.000**	0.019−**0.005**	0.008−**0.001**	0.051+**0.001**
Runtime [s]	0.166+**0.834**	0.335+**1.677**	0.005+**0.093**	3.124+**24.957**	1.371+**8.926**	3.126+**24.053**
(at N=10 ± change per 10)						

High-dimensionality II ($L = 2N$, $T = 300$) ParCorr	PC_1+MCI_3	PC_1+MCI_0	PC_1+MCI_0pw	PC_1^α+MCI_{all}	PC_1+MCI_{all}
TPR	0.895−**0.009**	0.925−**0.006**	0.897−**0.013**	0.893−**0.012**	0.884−**0.019**
FPR	0.050+**0.001**	0.051+**0.001**	0.078−**0.003**	0.051−**0.000**	0.051+**0.001**
Runtime [s]	3.046+**21.898**	3.008+**21.593**	4.269+**45.061**	0.956+**6.429**	3.126+**24.053**
(at N=10 ± change per 10)					

Table S6. Summarized ANOVA results for high-dimensionality GPDC and CMI experiments in Supplementary Tab. S2. See Supplementary Sect. S6.3 for an explanation.

High-dimensionality GPDC	Corr	FullCI	PC	PC_1+MCI_3	PC_1+MCI_{all}
TPR	$0.929 - 0.049$	$0.835 - 0.019$	$0.442 + 0.041$	$0.857 - 0.015$	$0.863 - 0.023$
FPR	0.650−**0.110**	0.056+**0.048**	0.023−**0.022**	0.053+**0.006**	0.056+**0.004**
Runtime [min] (at N=2 ± change per 10)	0.903+**0.930**	4.323+**192.531**	11.239+**510.892**	8.432+**475.658**	8.502+**505.743**

High-dimensionality CMI	Corr	FullCI	PC	PC_1+MCI_3	PC_1+MCI_{all}
TPR	$0.952 - 0.195$	0.612−**0.822**	$0.653 - 0.295$	0.884−**0.633**	0.891−**0.686**
FPR	0.604−**0.456**	0.039−**0.008**	$0.009 - 0.009$	0.048−**0.018**	0.047−**0.018**
Runtime [h] (at N=2 ± change per 10)	0.005+**0.209**	0.043+**3.182**	0.055+**1.381**	0.057+**1.520**	0.057+**1.546**

Table S7. Summarized ANOVA results for sample size experiments in Supplementary Tab. S2. See Supplementary Sect. S6.3 for an explanation.

Sample size ParCorr	FullCI	Lasso	PC	PC_1+MCI_{all}
TPR	0.245+**0.239**	0.500+**0.131**	0.234+**0.129**	0.530+**0.162**
FPR	0.053−**0.002**	0.011−**0.002**	0.005+**0.000**	0.054−**0.001**
Runtime [s]	0.039−**0.002**	14.941−**1.265**	4.786+**0.702**	11.699+**0.995**
(at T=150 ± change per 100)				

Sample size GPDC	FullCI	PC	PC_1+MCI_{all}
TPR	0.645+**0.078**	0.332+**0.088**	0.668+**0.079**
FPR	0.088−**0.005**	0.005 + 0.000	0.059−**0.001**
Runtime [min]	99.252+**158.660**	233.773+**464.634**	217.530+**361.099**
(at T=250 ± change per 100)			

Sample size CMI	FullCI	PC	PC_1+MCI_{all}
TPR	0.290+**0.028**	0.552+**0.042**	0.627+**0.036**
FPR	0.037 + 0.001	0.005 − 0.000	0.039 + 0.000
Runtime [h]	0.530+**0.237**	0.413+**0.148**	0.411+**0.134**
(at T=500 ± change per 100)			

Table S8. Summarized ANOVA results for observational noise and non-stationary trend experiments in Supplementary Tab. S2. See Supplementary Sect. S6.3 for an explanation.

Observational noise ParCorr	FullCI	Lasso	PC	PC_1+MCI_{all}
TPR	0.433−**0.188**	0.746−**0.241**	0.403−**0.100**	0.815−**0.296**
FPR	0.052−**0.001**	0.011+**0.003**	0.004+**0.002**	0.053+**0.003**
Runtime [s]	0.035 + 0.005	15.179+**0.897**	4.816+**0.608**	11.612+**0.636**
(at σ=0.0 ± change per 1)				

Non-stationarity ParCorr	FullCI	Lasso	PC	PC_1+MCI_{all}
TPR	0.436−**0.013**	0.745−**0.074**	0.402−**0.079**	0.812−**0.029**
FPR	0.052+**0.001**	0.011+**0.006**	0.004+**0.001**	0.053+**0.002**
Runtime [s]	0.036−**0.000**	16.669 − 0.198	4.995+**0.842**	12.128+**0.983**
(at a=0.0 ± change per 1)				

Table S9. ANCOVA results for FullCI on the ANCOVA experiment in Supplementary Tab. S2 to study interaction effects between N and T. See Supplementary Sect. S6.3 for further details. The upper and lower table show the ANCOVA results for detection power and false positives, respectively, as a response variable.

	**coef**	**std err**	**t**	**P>|t|**	**[0.025**	**0.975]**
**Intercept**	0.5780	0.020	29.417	0.000	0.539	0.617
**C(N)[T.5]**	-0.0657	0.022	-3.052	0.002	-0.108	-0.023
**C(N)[T.10]**	-0.1474	0.021	-7.155	0.000	-0.188	-0.107
**C(N)[T.20]**	-0.3329	0.020	-16.533	0.000	-0.372	-0.293
**C(T)[T.200]**	0.1415	0.028	5.092	0.000	0.087	0.196
**C(T)[T.300]**	0.2805	0.028	10.095	0.000	0.226	0.335
**C(N)[T.5]:C(T)[T.200]**	0.0112	0.030	0.368	0.713	-0.048	0.071
**C(N)[T.10]:C(T)[T.200]**	0.0349	0.029	1.199	0.231	-0.022	0.092
**C(N)[T.20]:C(T)[T.200]**	0.0642	0.028	2.254	0.024	0.008	0.120
**C(N)[T.5]:C(T)[T.300]**	0.0518	0.030	1.702	0.089	-0.008	0.111
**C(N)[T.10]:C(T)[T.300]**	0.0995	0.029	3.414	0.001	0.042	0.157
**C(N)[T.20]:C(T)[T.300]**	0.1951	0.028	6.851	0.000	0.139	0.251

	**coef**	**std err**	**t**	**P>|t|**	**[0.025**	**0.975]**
**Intercept**	0.0516	0.002	27.991	0.000	0.048	0.055
**C(N)[T.5]**	-0.0033	0.002	-1.708	0.088	-0.007	0.000
**C(N)[T.10]**	-0.0039	0.002	-2.116	0.034	-0.008	-0.000
**C(N)[T.20]**	0.0018	0.002	0.992	0.321	-0.002	0.005
**C(T)[T.200]**	-0.0002	0.003	-0.085	0.932	-0.005	0.005
**C(T)[T.300]**	-0.0011	0.003	-0.405	0.686	-0.006	0.004
**C(N)[T.5]:C(T)[T.200]**	0.0020	0.003	0.732	0.464	-0.003	0.007
**C(N)[T.10]:C(T)[T.200]**	0.0028	0.003	1.056	0.291	-0.002	0.008
**C(N)[T.20]:C(T)[T.200]**	0.0004	0.003	0.142	0.887	-0.005	0.005
**C(N)[T.5]:C(T)[T.300]**	0.0019	0.003	0.688	0.492	-0.003	0.007
**C(N)[T.10]:C(T)[T.300]**	0.0019	0.003	0.731	0.465	-0.003	0.007
**C(N)[T.20]:C(T)[T.300]**	-0.0032	0.003	-1.216	0.224	-0.008	0.002

Table S10. ANCOVA results for Lasso on the ANCOVA experiment in Supplementary Tab. S2 to study interaction effects between N and T. See Supplementary Sect. S6.3 for further details. The upper and lower table show the ANCOVA results for detection power and false positives, respectively, as a response variable.

	**coef**	**std err**	**t**	**P>|t|**	**[0.025**	**0.975]**
**Intercept**	0.6155	0.052	11.940	0.000	0.514	0.717
**C(N)[T.5]**	0.0137	0.056	0.243	0.808	-0.097	0.124
**C(N)[T.10]**	-0.0680	0.054	-1.257	0.209	-0.174	0.038
**C(N)[T.20]**	-0.1155	0.053	-2.186	0.029	-0.219	-0.012
**C(N)[T.40]**	-0.1422	0.052	-2.725	0.006	-0.245	-0.040
**C(N)[T.60]**	-0.1971	0.052	-3.792	0.000	-0.299	-0.095
**C(N)[T.80]**	-0.2053	0.052	-3.958	0.000	-0.307	-0.104
**C(N)[T.100]**	-0.2365	0.052	-4.564	0.000	-0.338	-0.135
**C(T)[T.200]**	0.1345	0.073	1.845	0.065	-0.008	0.277
**C(T)[T.300]**	0.2735	0.073	3.752	0.000	0.131	0.416
**C(N)[T.5]:C(T)[T.200]**	-0.0134	0.080	-0.168	0.867	-0.170	0.143
**C(N)[T.10]:C(T)[T.200]**	-0.0095	0.076	-0.125	0.901	-0.159	0.140
**C(N)[T.20]:C(T)[T.200]**	-0.0141	0.075	-0.189	0.850	-0.161	0.132
**C(N)[T.40]:C(T)[T.200]**	-0.0079	0.074	-0.107	0.915	-0.153	0.137
**C(N)[T.60]:C(T)[T.200]**	-0.0152	0.074	-0.206	0.836	-0.159	0.129
**C(N)[T.80]:C(T)[T.200]**	-0.0117	0.073	-0.159	0.874	-0.155	0.132
**C(N)[T.100]:C(T)[T.200]**	-0.0182	0.073	-0.248	0.804	-0.162	0.125
**C(N)[T.5]:C(T)[T.300]**	-0.0123	0.080	-0.154	0.878	-0.169	0.144
**C(N)[T.10]:C(T)[T.300]**	-0.0164	0.076	-0.215	0.830	-0.166	0.133
**C(N)[T.20]:C(T)[T.300]**	-0.0158	0.075	-0.211	0.833	-0.162	0.131
**C(N)[T.40]:C(T)[T.300]**	0.0109	0.074	0.148	0.882	-0.134	0.156
**C(N)[T.60]:C(T)[T.300]**	-0.0114	0.074	-0.155	0.877	-0.155	0.133
**C(N)[T.80]:C(T)[T.300]**	-0.0005	0.073	-0.006	0.995	-0.144	0.143
**C(N)[T.100]:C(T)[T.300]**	-0.0124	0.073	-0.169	0.866	-0.156	0.131

	**coef**	**std err**	**t**	**P>|t|**	**[0.025**	**0.975]**
**Intercept**	0.0421	0.001	63.080	0.000	0.041	0.043
**C(N)[T.5]**	-0.0159	0.001	-22.835	0.000	-0.017	-0.015
**C(N)[T.10]**	-0.0257	0.001	-38.134	0.000	-0.027	-0.024
**C(N)[T.20]**	-0.0310	0.001	-46.324	0.000	-0.032	-0.030
**C(N)[T.40]**	-0.0346	0.001	-51.889	0.000	-0.036	-0.033
**C(N)[T.60]**	-0.0365	0.001	-54.733	0.000	-0.038	-0.035
**C(N)[T.80]**	-0.0373	0.001	-55.934	0.000	-0.039	-0.036
**C(N)[T.100]**	-0.0380	0.001	-56.991	0.000	-0.039	-0.037
**C(T)[T.200]**	-0.0006	0.001	-0.589	0.556	-0.002	0.001
**C(T)[T.300]**	-0.0065	0.001	-6.894	0.000	-0.008	-0.005
**C(N)[T.5]:C(T)[T.200]**	-0.0015	0.001	-1.566	0.117	-0.003	0.000
**C(N)[T.10]:C(T)[T.200]**	-0.0013	0.001	-1.335	0.182	-0.003	0.001
**C(N)[T.20]:C(T)[T.200]**	-0.0013	0.001	-1.406	0.160	-0.003	0.001
**C(N)[T.40]:C(T)[T.200]**	-0.0012	0.001	-1.231	0.218	-0.003	0.001
**C(N)[T.60]:C(T)[T.200]**	-0.0009	0.001	-0.935	0.350	-0.003	0.001
**C(N)[T.80]:C(T)[T.200]**	-0.0008	0.001	-0.864	0.388	-0.003	0.001
**C(N)[T.100]:C(T)[T.200]**	-0.0007	0.001	-0.691	0.490	-0.002	0.001
**C(N)[T.5]:C(T)[T.300]**	0.0010	0.001	1.046	0.296	-0.001	0.003
**C(N)[T.10]:C(T)[T.300]**	0.0025	0.001	2.650	0.008	0.001	0.004
**C(N)[T.20]:C(T)[T.300]**	0.0025	0.001	2.696	0.007	0.001	0.004
**C(N)[T.40]:C(T)[T.300]**	0.0030	0.001	3.211	0.001	0.001	0.005
**C(N)[T.60]:C(T)[T.300]**	0.0037	0.001	3.896	0.000	0.002	0.006
**C(N)[T.80]:C(T)[T.300]**	0.0039	0.001	4.126	0.000	0.002	0.006
**C(N)[T.100]:C(T)[T.300]**	0.0042	0.001	4.497	0.000	0.002	0.006

Table S11. ANCOVA results for PC on the ANCOVA experiment in Supplementary Tab. S2 to study interaction effects between N and T. See Supplementary Sect. S6.3 for further details. The upper and lower table show the ANCOVA results for detection power and false positives, respectively, as a response variable.

	**coef**	**std err**	**t**	**P>|t|**	**[0.025**	**0.975]**
**Intercept**	0.3315	0.049	6.704	0.000	0.235	0.428
**C(N)[T.5]**	-0.0883	0.054	-1.630	0.103	-0.194	0.018
**C(N)[T.10]**	-0.0807	0.052	-1.556	0.120	-0.182	0.021
**C(N)[T.20]**	-0.0970	0.051	-1.914	0.056	-0.196	0.002
**C(N)[T.40]**	-0.0637	0.050	-1.273	0.203	-0.162	0.034
**C(N)[T.60]**	-0.0881	0.050	-1.766	0.077	-0.186	0.010
**C(N)[T.80]**	-0.0895	0.050	-1.799	0.072	-0.187	0.008
**C(N)[T.100]**	-0.0969	0.050	-1.951	0.051	-0.194	0.000
**C(T)[T.200]**	0.0710	0.070	1.015	0.310	-0.066	0.208
**C(T)[T.300]**	0.2255	0.070	3.225	0.001	0.088	0.363
**C(N)[T.5]:C(T)[T.200]**	0.0183	0.077	0.239	0.811	-0.132	0.168
**C(N)[T.10]:C(T)[T.200]**	0.0353	0.073	0.481	0.631	-0.109	0.179
**C(N)[T.20]:C(T)[T.200]**	0.0276	0.072	0.384	0.701	-0.113	0.168
**C(N)[T.40]:C(T)[T.200]**	0.0479	0.071	0.676	0.499	-0.091	0.187
**C(N)[T.60]:C(T)[T.200]**	0.0347	0.071	0.492	0.623	-0.103	0.173
**C(N)[T.80]:C(T)[T.200]**	0.0399	0.070	0.567	0.571	-0.098	0.178
**C(N)[T.100]:C(T)[T.200]**	0.0406	0.070	0.578	0.563	-0.097	0.178
**C(N)[T.5]:C(T)[T.300]**	0.0106	0.077	0.138	0.890	-0.140	0.161
**C(N)[T.10]:C(T)[T.300]**	0.0254	0.073	0.346	0.729	-0.118	0.169
**C(N)[T.20]:C(T)[T.300]**	0.0183	0.072	0.255	0.799	-0.122	0.159
**C(N)[T.40]:C(T)[T.300]**	0.0574	0.071	0.811	0.417	-0.081	0.196
**C(N)[T.60]:C(T)[T.300]**	0.0382	0.071	0.542	0.588	-0.100	0.176
**C(N)[T.80]:C(T)[T.300]**	0.0495	0.070	0.703	0.482	-0.088	0.187
**C(N)[T.100]:C(T)[T.300]**	0.0483	0.070	0.688	0.492	-0.089	0.186

	**coef**	**std err**	**t**	**P>|t|**	**[0.025**	**0.975]**
**Intercept**	0.0211	0.001	34.132	0.000	0.020	0.022
**C(N)[T.5]**	-0.0105	0.001	-16.161	0.000	-0.012	-0.009
**C(N)[T.10]**	-0.0148	0.001	-23.674	0.000	-0.016	-0.014
**C(N)[T.20]**	-0.0165	0.001	-26.546	0.000	-0.018	-0.015
**C(N)[T.40]**	-0.0179	0.001	-28.887	0.000	-0.019	-0.017
**C(N)[T.60]**	-0.0184	0.001	-29.799	0.000	-0.020	-0.017
**C(N)[T.80]**	-0.0188	0.001	-30.315	0.000	-0.020	-0.018
**C(N)[T.100]**	-0.0190	0.001	-30.686	0.000	-0.020	-0.018
**C(T)[T.200]**	-0.0020	0.001	-2.286	0.022	-0.004	-0.000
**C(T)[T.300]**	-0.0011	0.001	-1.270	0.204	-0.003	0.001
**C(N)[T.5]:C(T)[T.200]**	0.0020	0.001	2.203	0.028	0.000	0.004
**C(N)[T.10]:C(T)[T.200]**	0.0020	0.001	2.271	0.023	0.000	0.004
**C(N)[T.20]:C(T)[T.200]**	0.0021	0.001	2.377	0.017	0.000	0.004
**C(N)[T.40]:C(T)[T.200]**	0.0021	0.001	2.453	0.014	0.000	0.004
**C(N)[T.60]:C(T)[T.200]**	0.0022	0.001	2.539	0.011	0.001	0.004
**C(N)[T.80]:C(T)[T.200]**	0.0022	0.001	2.557	0.011	0.001	0.004
**C(N)[T.100]:C(T)[T.200]**	0.0022	0.001	2.547	0.011	0.001	0.004
**C(N)[T.5]:C(T)[T.300]**	0.0009	0.001	0.973	0.331	-0.001	0.003
**C(N)[T.10]:C(T)[T.300]**	0.0011	0.001	1.189	0.234	-0.001	0.003
**C(N)[T.20]:C(T)[T.300]**	0.0014	0.001	1.574	0.115	-0.000	0.003
**C(N)[T.40]:C(T)[T.300]**	0.0015	0.001	1.687	0.092	-0.000	0.003
**C(N)[T.60]:C(T)[T.300]**	0.0016	0.001	1.882	0.060	-6.81e-05	0.003
**C(N)[T.80]:C(T)[T.300]**	0.0017	0.001	1.894	0.058	-5.8e-05	0.003
**C(N)[T.100]:C(T)[T.300]**	0.0017	0.001	1.920	0.055	-3.47e-05	0.003

Table S12. ANCOVA results for FullCI on the ANCOVA experiment in Supplementary Tab. S2 to study interaction effects between N and T. See Supplementary Sect. S6.3 for further details. The upper and lower table show the ANCOVA results for detection power and false positives, respectively, as a response variable.

	**coef**	**std err**	**t**	**P>|t|**	**[0.025**	**0.975]**
**Intercept**	0.6180	0.018	34.392	0.000	0.583	0.653
**C(N)[T.5]**	-0.0301	0.020	-1.529	0.126	-0.069	0.008
**C(N)[T.10]**	-0.0519	0.019	-2.751	0.006	-0.089	-0.015
**C(N)[T.20]**	-0.0877	0.018	-4.764	0.000	-0.124	-0.052
**C(N)[T.40]**	-0.1495	0.018	-8.217	0.000	-0.185	-0.114
**C(N)[T.60]**	-0.1927	0.018	-10.634	0.000	-0.228	-0.157
**C(N)[T.80]**	-0.2185	0.018	-12.082	0.000	-0.254	-0.183
**C(N)[T.100]**	-0.2463	0.018	-13.639	0.000	-0.282	-0.211
**C(T)[T.200]**	0.1365	0.025	5.371	0.000	0.087	0.186
**C(T)[T.300]**	0.2980	0.025	11.726	0.000	0.248	0.348
**C(N)[T.5]:C(T)[T.200]**	0.0057	0.028	0.205	0.838	-0.049	0.060
**C(N)[T.10]:C(T)[T.200]**	0.0152	0.027	0.568	0.570	-0.037	0.067
**C(N)[T.20]:C(T)[T.200]**	0.0105	0.026	0.404	0.686	-0.041	0.062
**C(N)[T.40]:C(T)[T.200]**	0.0238	0.026	0.924	0.355	-0.027	0.074
**C(N)[T.60]:C(T)[T.200]**	0.0276	0.026	1.077	0.281	-0.023	0.078
**C(N)[T.80]:C(T)[T.200]**	0.0223	0.026	0.872	0.383	-0.028	0.072
**C(N)[T.100]:C(T)[T.200]**	0.0227	0.026	0.888	0.375	-0.027	0.073
**C(N)[T.5]:C(T)[T.300]**	-0.0010	0.028	-0.036	0.971	-0.056	0.054
**C(N)[T.10]:C(T)[T.300]**	0.0169	0.027	0.632	0.527	-0.035	0.069
**C(N)[T.20]:C(T)[T.300]**	0.0358	0.026	1.374	0.170	-0.015	0.087
**C(N)[T.40]:C(T)[T.300]**	0.0721	0.026	2.802	0.005	0.022	0.123
**C(N)[T.60]:C(T)[T.300]**	0.0831	0.026	3.242	0.001	0.033	0.133
**C(N)[T.80]:C(T)[T.300]**	0.0898	0.026	3.512	0.000	0.040	0.140
**C(N)[T.100]:C(T)[T.300]**	0.0955	0.026	3.740	0.000	0.045	0.146

	**coef**	**std err**	**t**	**P>|t|**	**[0.025**	**0.975]**
**Intercept**	0.0519	0.002	30.109	0.000	0.049	0.055
**C(N)[T.5]**	0.0006	0.002	0.310	0.757	-0.003	0.004
**C(N)[T.10]**	0.0003	0.002	0.144	0.885	-0.003	0.004
**C(N)[T.20]**	0.0023	0.002	1.355	0.175	-0.001	0.006
**C(N)[T.40]**	0.0032	0.002	1.875	0.061	-0.000	0.007
**C(N)[T.60]**	0.0032	0.002	1.869	0.062	-0.000	0.007
**C(N)[T.80]**	0.0029	0.002	1.696	0.090	-0.000	0.006
**C(N)[T.100]**	0.0027	0.002	1.539	0.124	-0.001	0.006
**C(T)[T.200]**	0.0014	0.002	0.593	0.553	-0.003	0.006
**C(T)[T.300]**	-0.0038	0.002	-1.573	0.116	-0.009	0.001
**C(N)[T.5]:C(T)[T.200]**	-0.0021	0.003	-0.814	0.416	-0.007	0.003
**C(N)[T.10]:C(T)[T.200]**	-0.0020	0.002	-0.823	0.411	-0.007	0.003
**C(N)[T.20]:C(T)[T.200]**	-0.0029	0.002	-1.175	0.240	-0.008	0.002
**C(N)[T.40]:C(T)[T.200]**	-0.0022	0.002	-0.917	0.359	-0.007	0.003
**C(N)[T.60]:C(T)[T.200]**	-0.0020	0.002	-0.800	0.424	-0.007	0.003
**C(N)[T.80]:C(T)[T.200]**	-0.0016	0.002	-0.661	0.509	-0.006	0.003
**C(N)[T.100]:C(T)[T.200]**	-0.0014	0.002	-0.584	0.559	-0.006	0.003
**C(N)[T.5]:C(T)[T.300]**	0.0023	0.003	0.890	0.373	-0.003	0.007
**C(N)[T.10]:C(T)[T.300]**	0.0027	0.002	1.089	0.276	-0.002	0.008
**C(N)[T.20]:C(T)[T.300]**	0.0012	0.002	0.503	0.615	-0.004	0.006
**C(N)[T.40]:C(T)[T.300]**	0.0015	0.002	0.613	0.540	-0.003	0.006
**C(N)[T.60]:C(T)[T.300]**	0.0024	0.002	0.986	0.324	-0.002	0.007
**C(N)[T.80]:C(T)[T.300]**	0.0031	0.002	1.271	0.204	-0.002	0.008
**C(N)[T.100]:C(T)[T.300]**	0.0036	0.002	1.465	0.143	-0.001	0.008

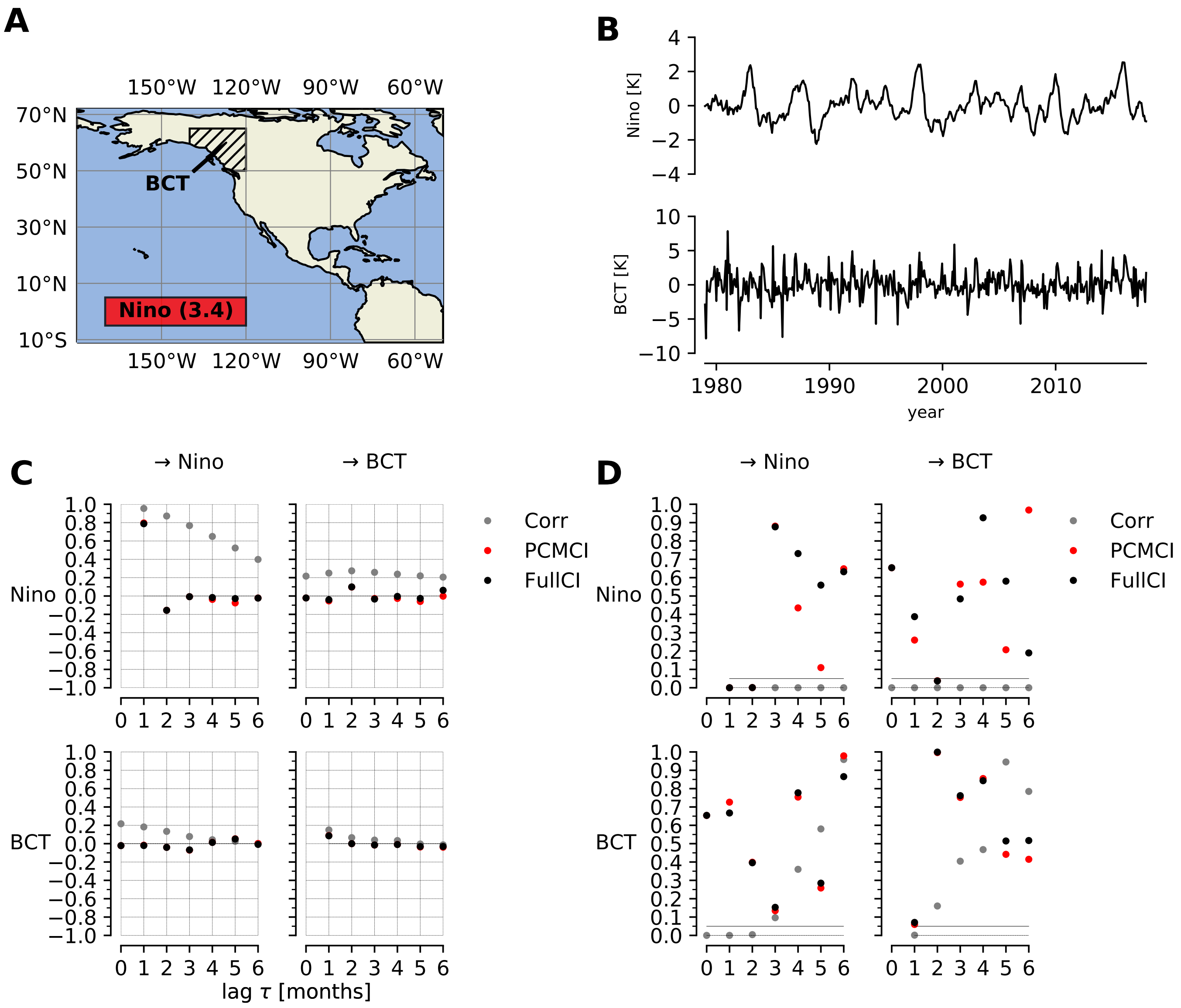

Fig. S2. Motivational climate example. (**A**) We investigate the relationship between the monthly climate index Nino and land temperature anomalies over Northwestern Canada, mostly British Columbia (BCT, hatched region). Nino is defined as the average sea-surface temperature anomaly (HadISST dataset (*27*)) over the red Nino3.4 region (5°North-5°South and 170-120°West). BCT is defined as the land surface temperature (CRUTEM4 dataset (*28*)) over British Columbia and parts of Yukon and the Northwestern Territories, Canada (50-65°North and 120-140°West). The grid location 62.5°North, 132.5°West was excluded since more than 1% of the samples were missing. Anomalized time series have the seasonal cycle removed. We constrain our analysis to the period with reliable satellite data (1979–2017) with a length of $T = 468$ months. To remove any long-term temperature trend, a Gaussian kernel smoothing mean with a bandwidth of $\sigma = 120$ months was removed from the raw time series. (**B**) Time series of Nino and BCT. (**C**) Matrix of lag functions between Nino and BCT for Correlation (Corr), PCMCI, and FullCI (conditional on the whole past of both time series up to $\tau_{\max} = 6$). Note that for autocorrelations (on the diagonal) the zero-lag is not drawn. (**D**) Matrix of p-values. The black line denotes the 5% significance level. Note that the sample size here is $n = T - 2\tau_{\max} = 456$. Note that in Fig. 2 we only depict links detected in at least 20% of the realizations.

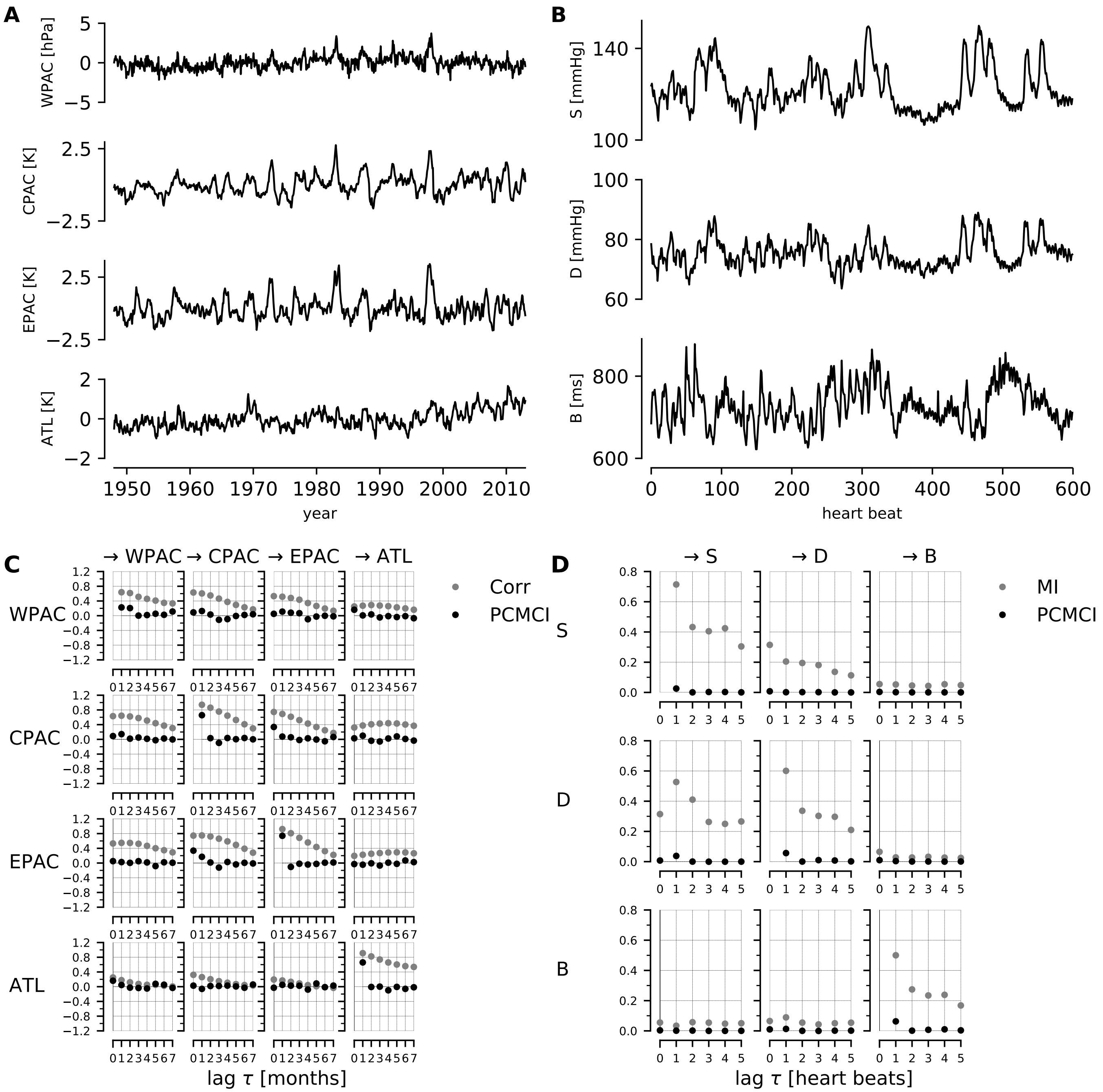

Fig. S3. **Real climate and cardiovascular applications.** See details on the data in Material and Methods. (**A**) Climate time series. (**B**) Cardiovascular time series for one of the 13 healthy women, sampled at heart beats. (**C**) Matrix of lag functions for climate example. (**D**) Matrix of average lag functions for cardiovascular example over all 13 subjects. See notes in Supplementary Fig. S2 for an explanation of lag functions.

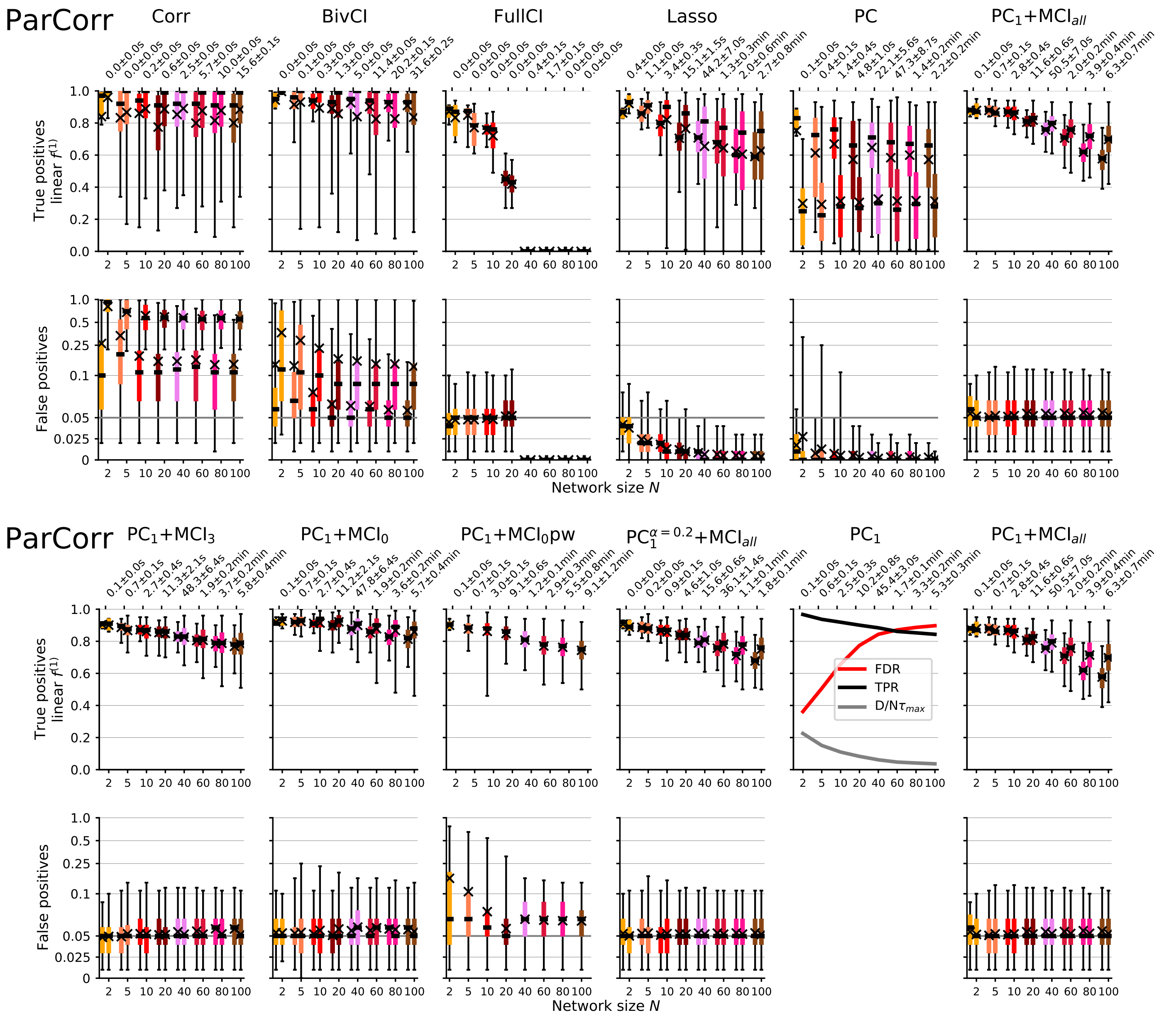

Fig. S4. **Experiments for linear models with short time series length.** Numerical experiments for linear models with different numbers of variables N, number of links $L = N$, and time series length $T = 150$. The detailed setup is listed in Supplementary Tab. S2 and Tab. S3 provides details on the evaluated methods. The second last column in the bottom row gives the dimensionality (grey line, as a fraction of $N\tau_{\max}$), true positive rate (TPR, black line) and false discovery rate (FDR, red line) of the PC_1 condition-selection stage. Note that in this panel true positives refer to the detection rate for *all* parents, cross-links as well as auto-links.

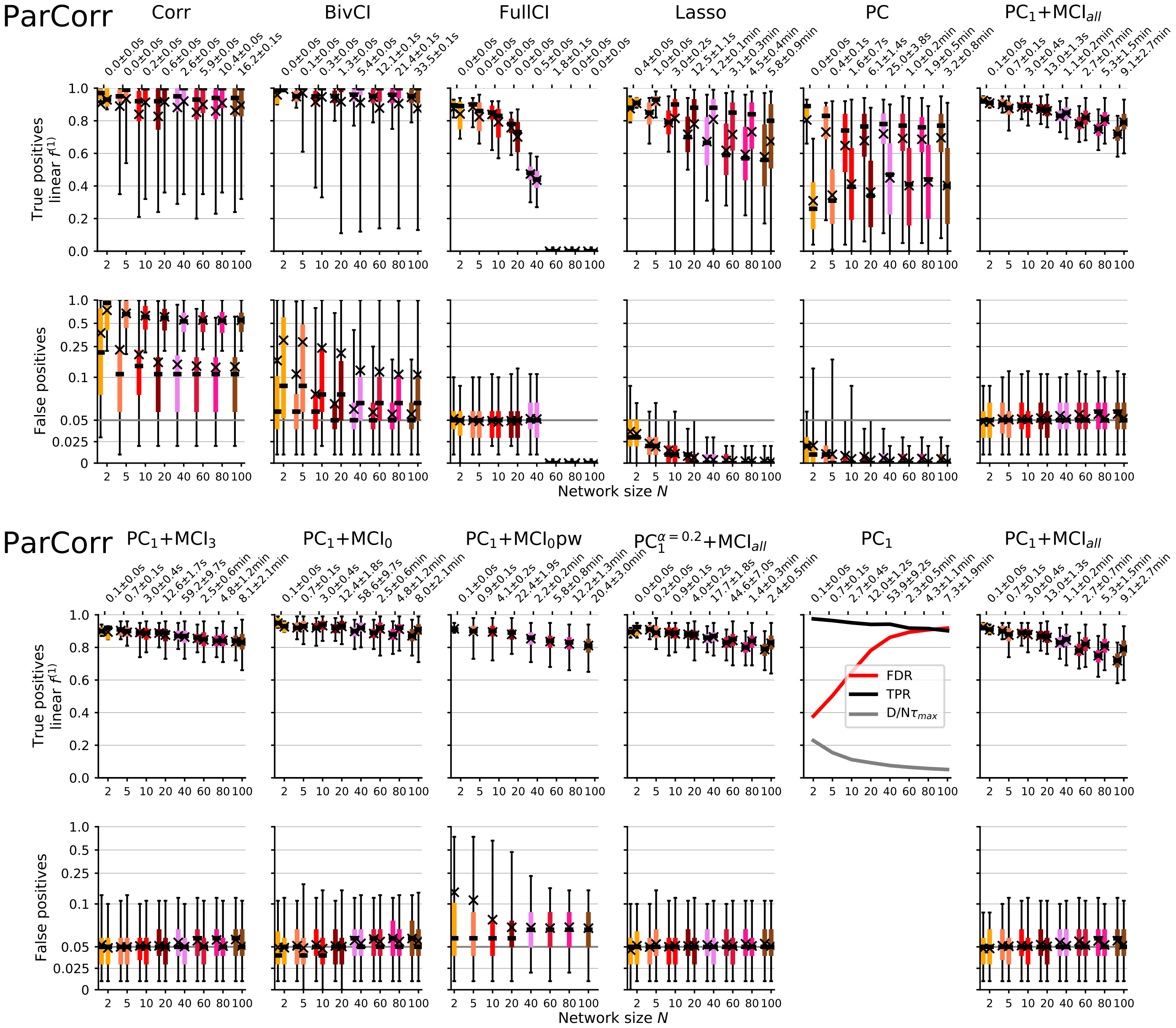

Fig. S5. **Experiments for linear models with longer time series length.** Numerical experiments for linear models with different numbers of variables N, number of links $L = N$, and time series length $T = 300$. The detailed setup is listed in Supplementary Tab. S2 and Tab. S3 provides details on the evaluated methods. The second last column in the bottom row gives the dimensionality (grey line, as a fraction of $N\tau_{\max}$), true positive rate (TPR, black line) and false discovery rate (FDR, red line) of the PC_1 condition-selection stage. Note that in this panel true positives refer to the detection rate for *all* parents, cross-links as well as auto-links.

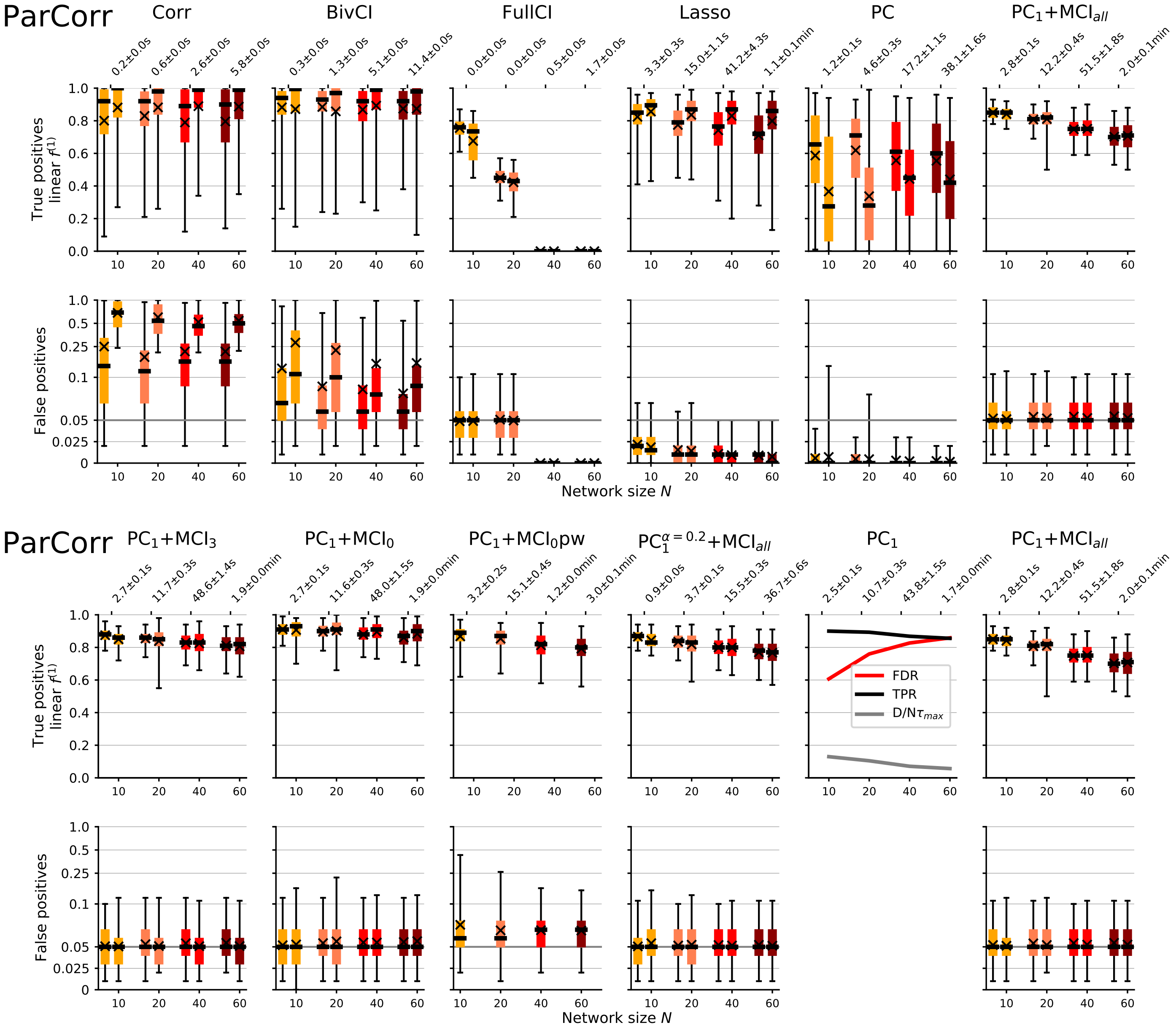

Fig. S6. **Experiments for dense linear models with short time series length.** Numerical experiments for linear models with different numbers of variables N, number of links $L = 2N$, and time series length $T = 150$. The detailed setup is listed in Supplementary Tab. S2 and Tab. S3 provides details on the evaluated methods. The second last column in the bottom row gives the dimensionality (grey line, as a fraction of $N\tau_{\max}$), true positive rate (TPR, black line) and false discovery rate (FDR, red line) of the PC_1 condition-selection stage. Note that in this panel true positives refer to the detection rate for *all* parents, cross-links as well as auto-links.

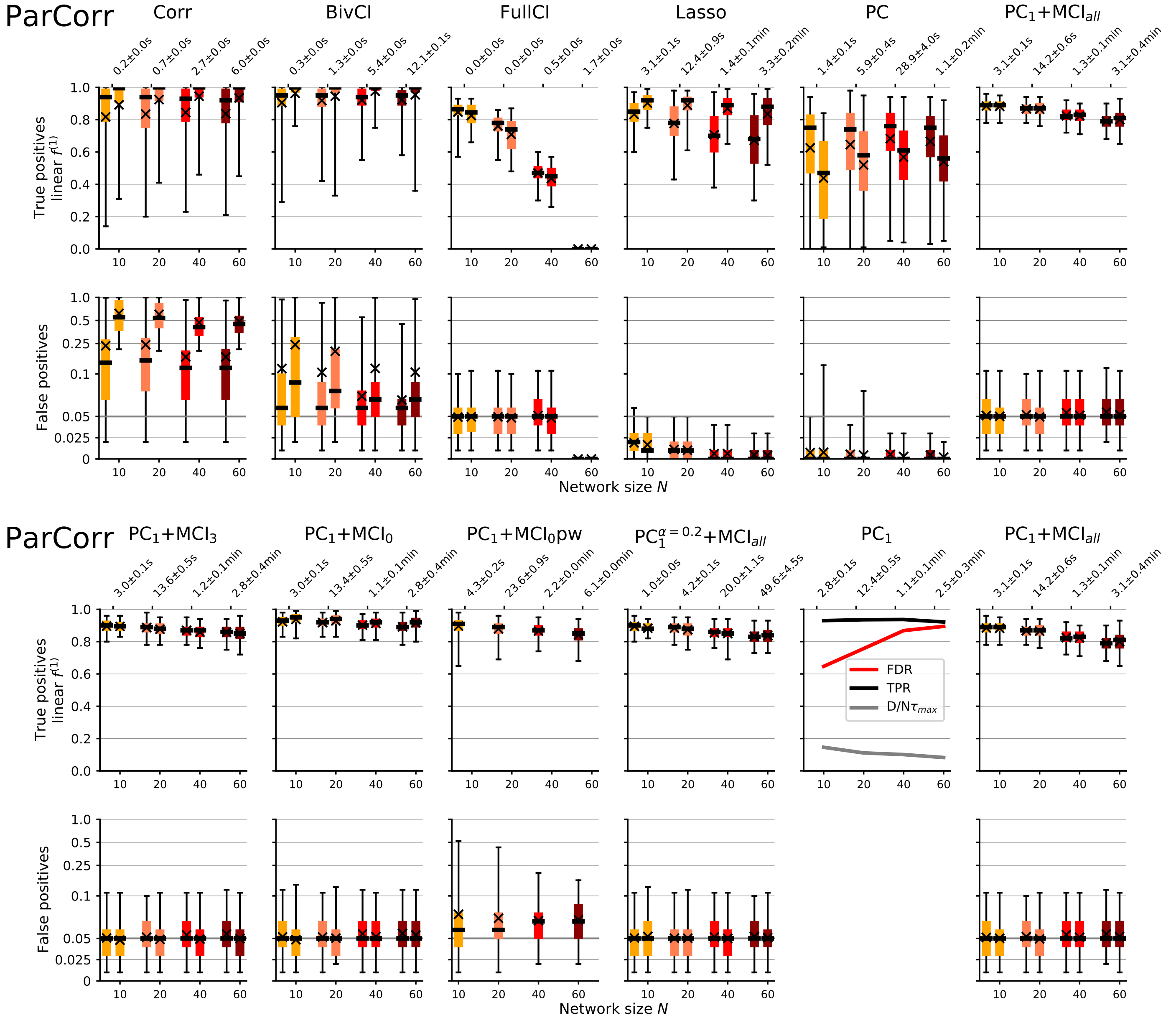

Fig. S7. **Experiments for dense linear models with longer time series length.** Numerical experiments for linear models with different numbers of variables N, number of links $L = 2N$, and time series length $T = 300$. The detailed setup is listed in Supplementary Tab. S2 and Tab. S3 provides details on the evaluated methods. The second last column in the bottom row gives the dimensionality (grey line, as a fraction of $N\tau_{\max}$), true positive rate (TPR, black line) and false discovery rate (FDR, red line) of the PC$_1$ condition-selection stage. Note that in this panel true positives refer to the detection rate for *all* parents, cross-links as well as auto-links.

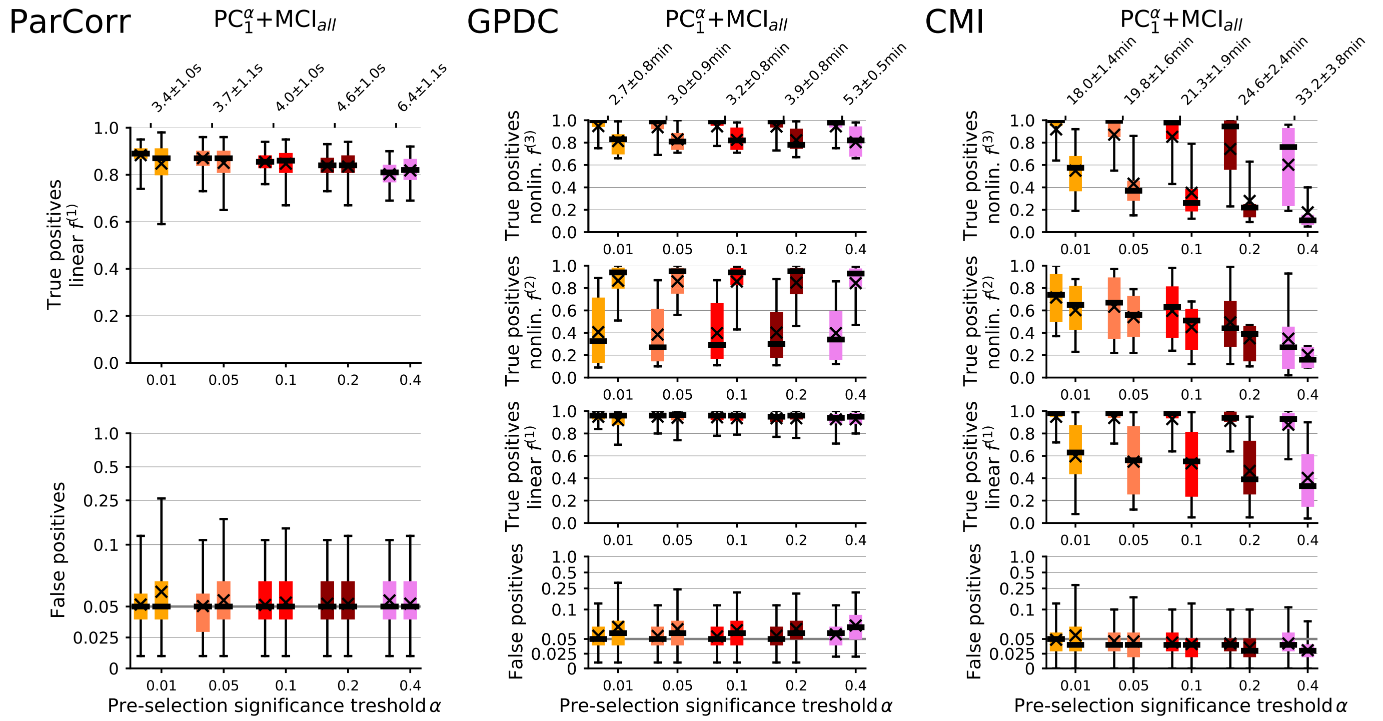

Fig. S8. **Experiments for different method parameters.** Influence of different PC thresholds $\alpha_{\rm PC}$ in the condition-selection Algorithm S1 for ParCorr (left), GPDC (center), and CMI (right). The detailed setup is listed in Supplementary Tab. S2 and Tab. S3 provides details on the evaluated methods. Note that for most numerical experiments for ParCorr we use an AIC-based optimization scheme to choose $\alpha_{\rm PC}$. See discussion in Materials and Methods.

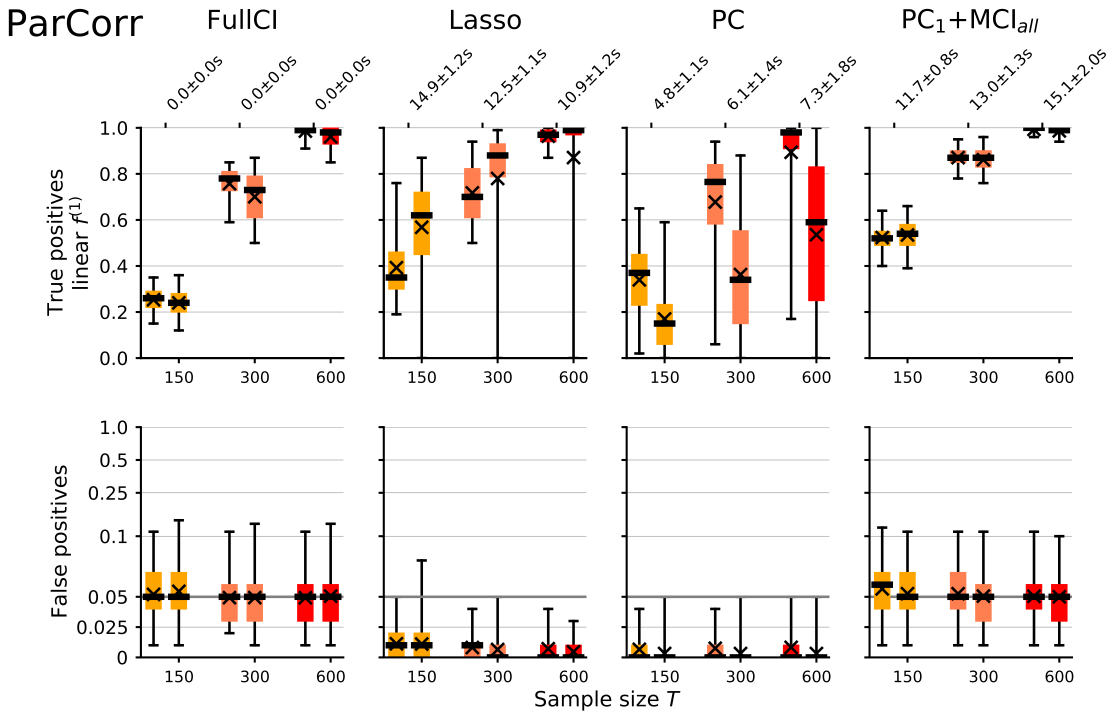

Fig. S9. **Experiments for linear methods with different sample sizes.**
Numerical experiments for linear models with different time series length T, fixed numbers of variables $N = 20$ and number of links $L = N$. The detailed setup is listed in Supplementary Tab. S2 and Tab. S3 provides details on the evaluated methods. For ParCorr, power levels go up with larger samples as expected for all methods. Still, PCMCI always outperforms FullCI, Lasso, and PC. Even for the largest sample size, Lasso still cannot detect some links. The runtime does not linearly increase with sample size like the individual partial correlation tests, because larger samples also lead to a faster convergence, for the PC_1 stage of PCMCI as well as for Lasso. An overview of runtimes for the experiments is shown in Supplementary Fig. S16.

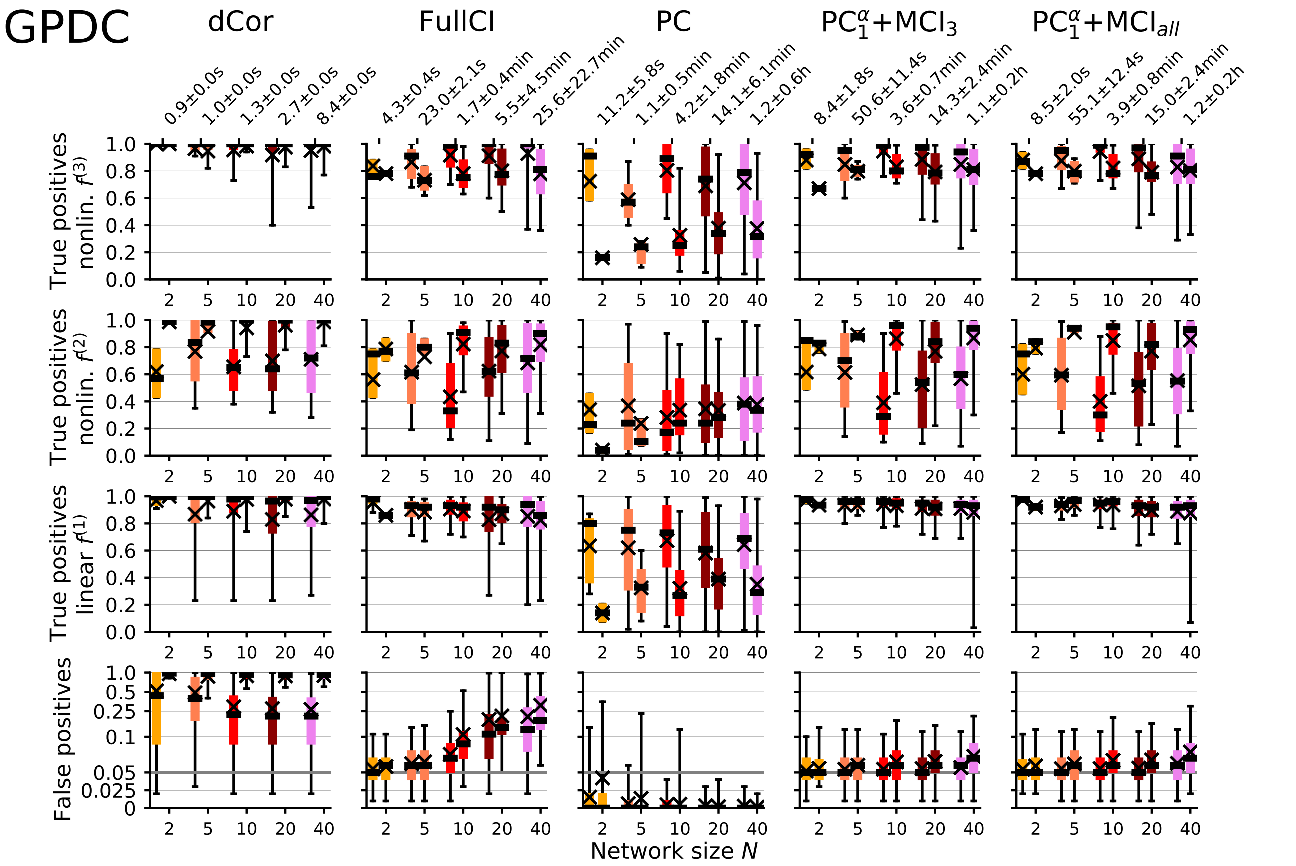

Fig. S10. **Experiments for nonlinear models (part 1).**

Numerical experiments for nonlinear models with different numbers of variables N, number of links $L = N$, and time series length $T = 250$. The detailed setup is listed in Supplementary Tab. S2 and Tab S3 provides details on the evaluated methods

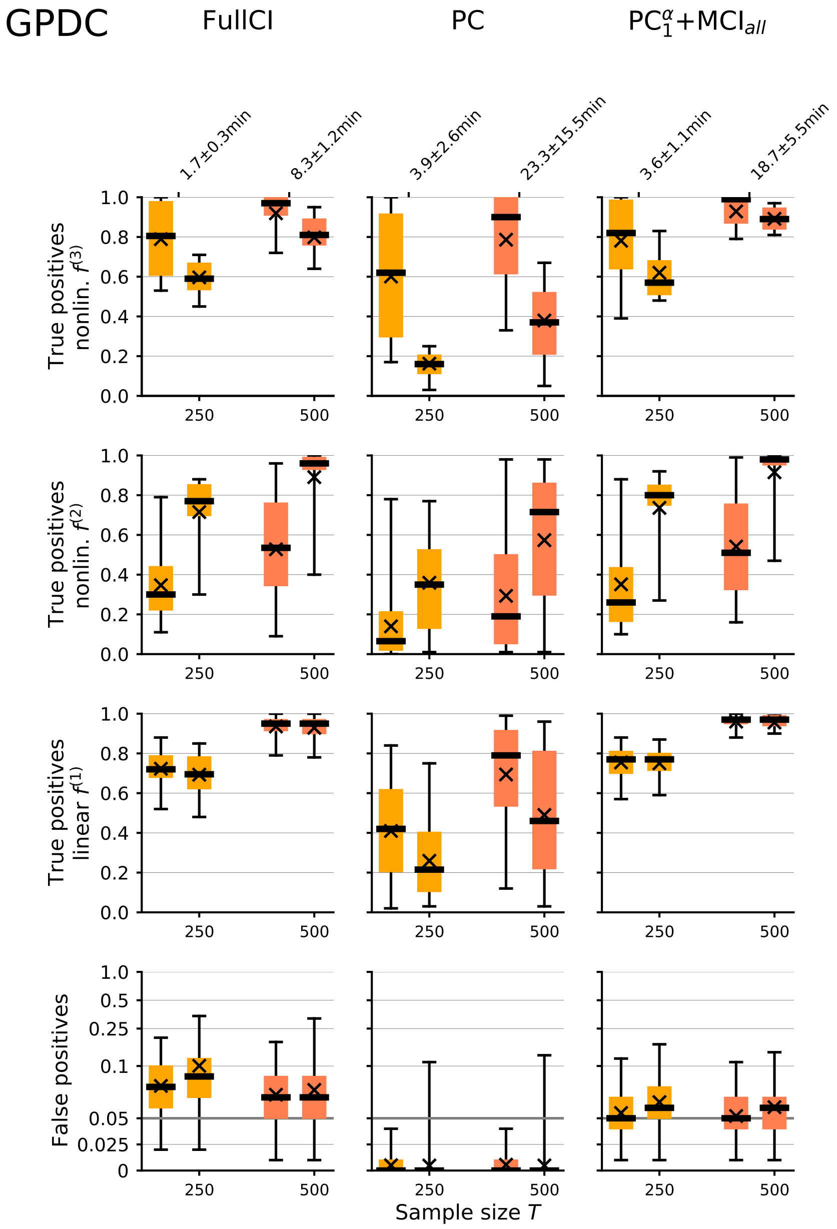

Fig. S11. **Experiments for nonlinear models with different sample sizes (part 1).** Numerical experiments for nonlinear models with different time series length T, fixed numbers of variables $N = 10$ and number of links $L = N$. The detailed setup is listed in Supplementary Tab. S2 and Tab. S3 provides details on the evaluated methods. Similarly, for GPDC power goes up as expected. Additionally, FullCI better controls false positives for larger sample size. Gaussian process regression's runtime scales as $\sim T^3$ with sample size making GPDC not very suitable for large sample sizes. However, there are efficient approximation methods of GP that can help to speed up estimation (*42*).

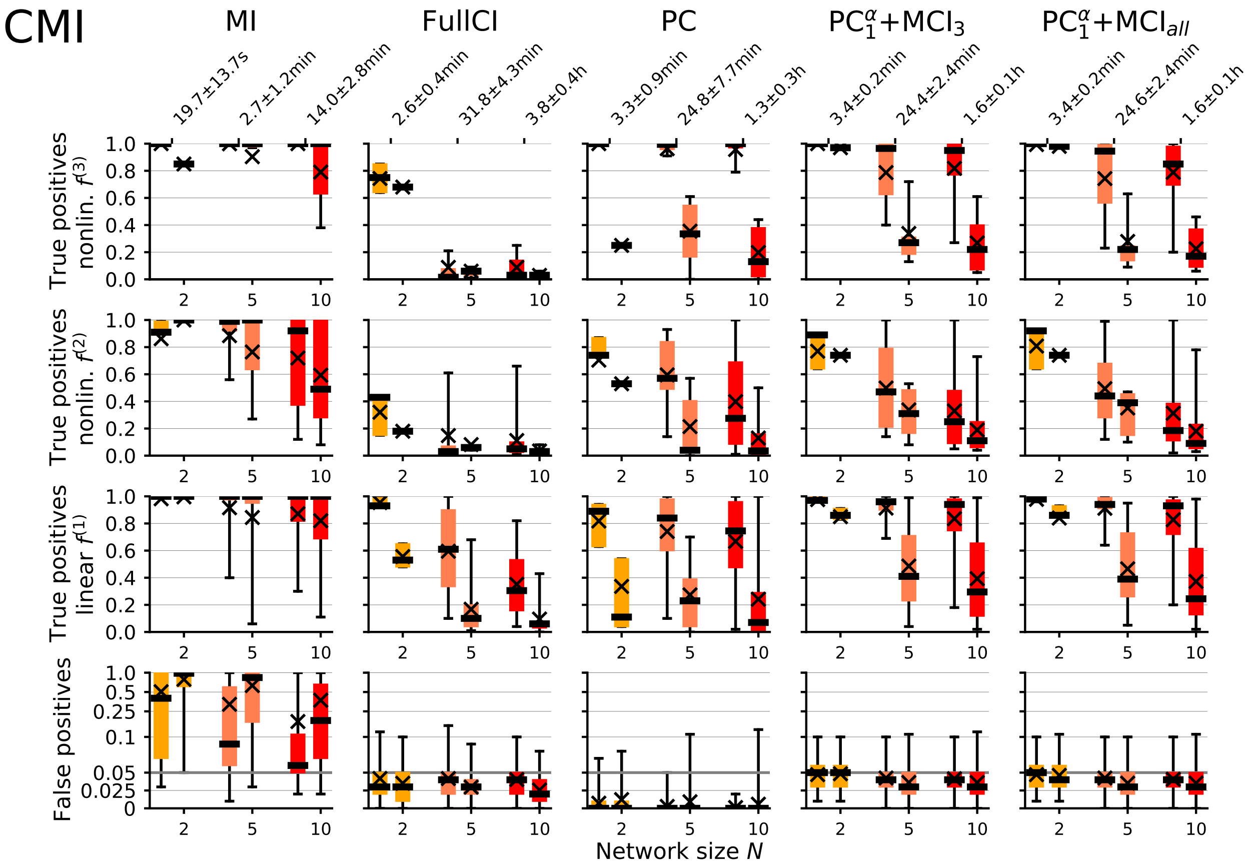

Fig. S12. **Experiments for nonlinear models (part 2).**

Numerical experiments for nonlinear models with different numbers of variables N, number of links $L = N$, and time series length $T = 500$. The detailed setup is listed in Supplementary Tab. S2 and Tab. S3 provides details on the evaluated methods.

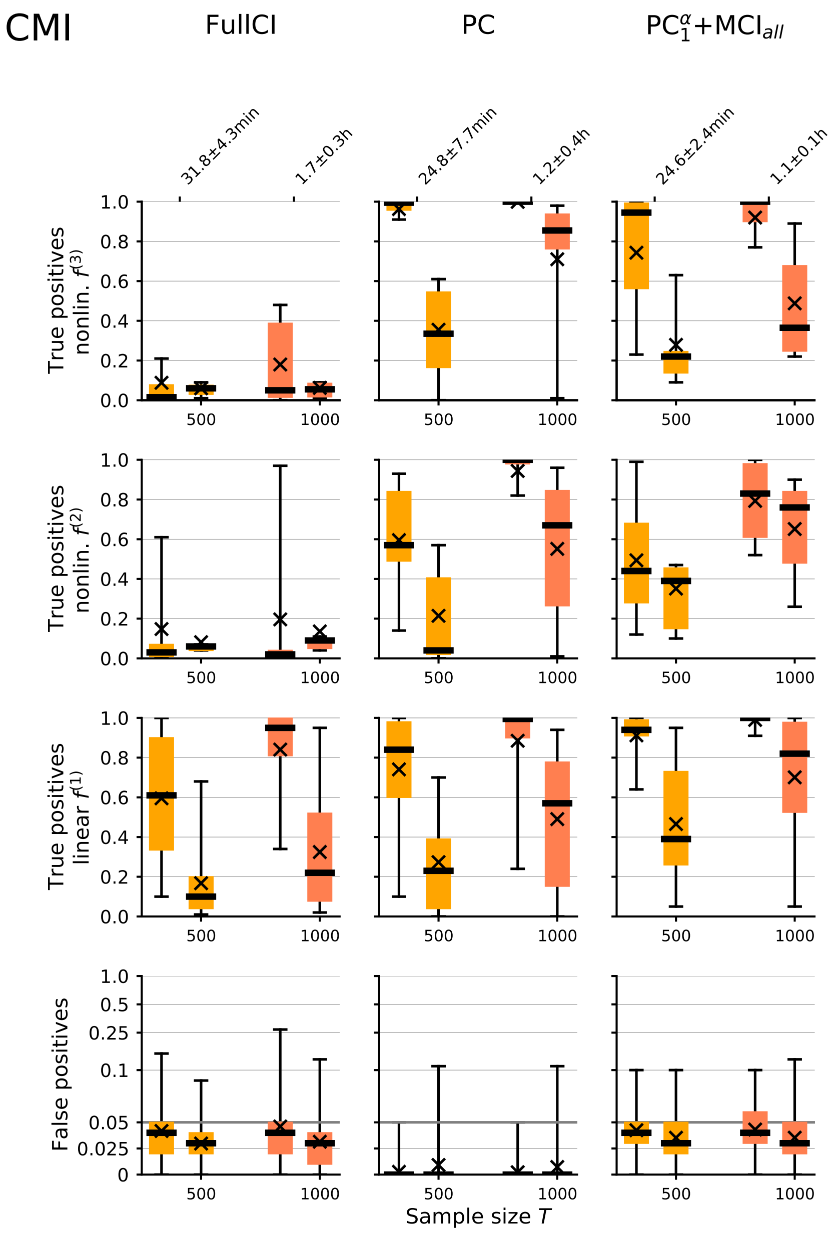

Fig. S13. **Experiments for nonlinear models with different sample sizes (part 2).**
Numerical experiments for nonlinear models with different time series lengths T, fixed numbers of variables $N = 5$ and number of links $L = N$. The detailed setup is listed in Supplementary Tab. S2 and Tab. S3 provides details on the evaluated methods. CMI has increasing power as expected. Here runtime does not increase quadratically like the individual CMI tests. An overview of runtimes for the experiments is shown in Fig. S16.

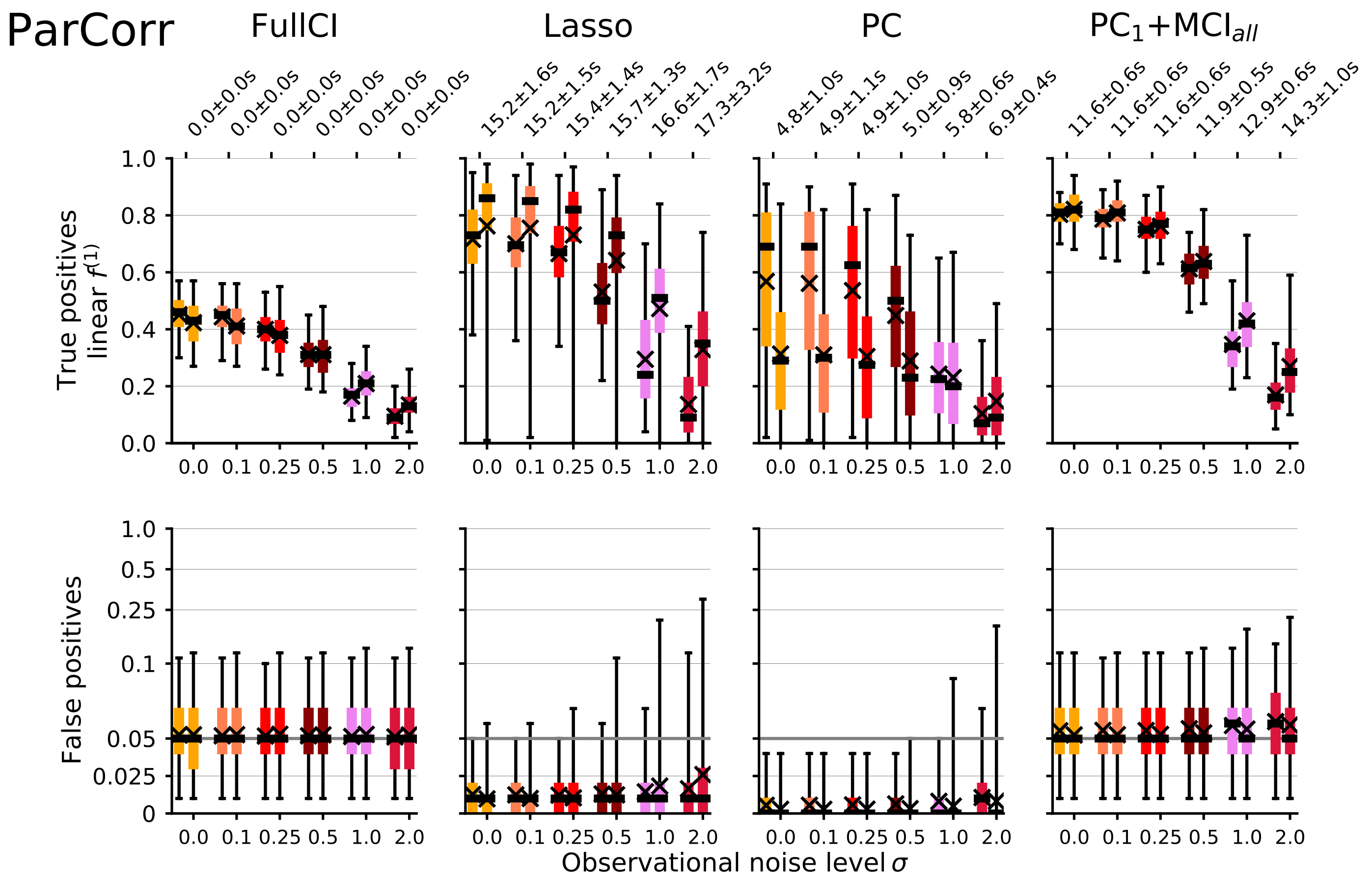

Fig. S14. **Experiments for observational noise models.**

Numerical experiments for linear models with added observational noise, fixed time series length $T = 150$, numbers of variables $N = 20$ and number of links $L = N$. The detailed setup is listed in Supplementary Tab. S2 and Tab. S3 provides details on the evaluated methods. Gaussian noise $\mathcal{N}(0, \sigma^2)$ with different standard deviations σ was added to the data. Note that the original time series were generated with dynamical noise with a standard deviation of one. All methods display a similar sensitivity to observational noise with levels up to 25% of the dynamical noise standard deviation having only minor effects. For levels of the same order as the dynamical noise we observe a stronger degradation, with also the false positives not being well-controlled since common drivers are essentially not well detected any longer. See ref. (*34*) for a discussion on observational error.

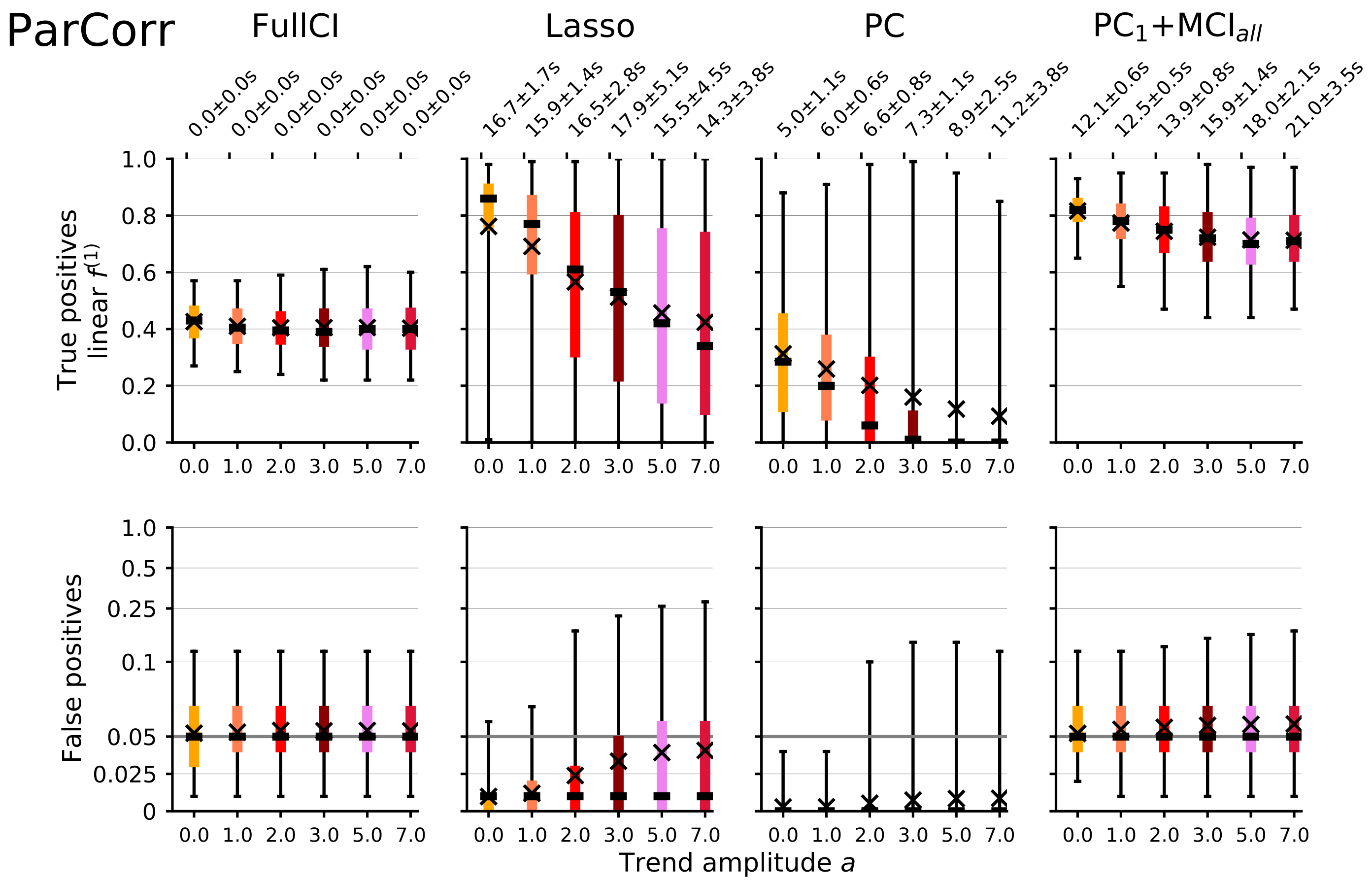

Fig. S15. **Experiments for nonstationary models.**

Numerical experiments for linear models with added non-stationary trend, fixed time series length $T = 150$, numbers of variables $N = 20$ and number of links $L = N$. The detailed setup is listed in Supplementary Tab. S2 and Tab. S3 provides details on the evaluated methods. Non-stationarity is modeled by adding a sinusoidal dependence $a \sin(2\pi t/25)$ with different amplitudes a to the data. Here Lasso and the PC algorithm are especially sensitive regarding both lower detection power and inflated false positives, while PCMCI is more robust even for high trend amplitudes. See ref. (*34*) for a discussion of non-stationarity in causal discovery.

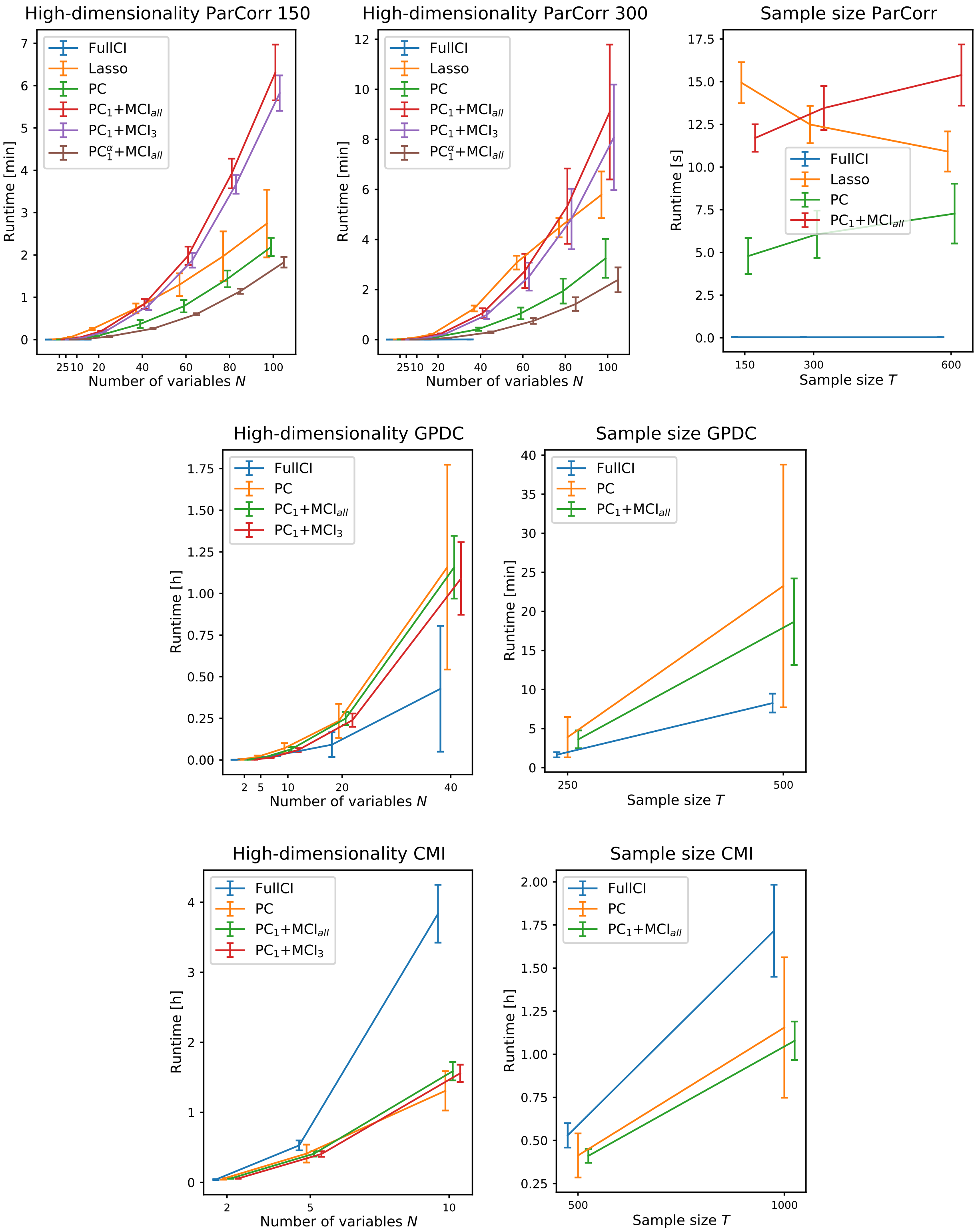

Fig. S16. **Runtimes for numerical experiments.**
Overview of runtimes for methods in numerical experiments as listed in Supplementary Tab. S2. Shown is the average runtime (in seconds, minutes, or hours) across all models together with its standard deviation as errorbars plotted against the number of variables and the sample size. The runtime estimates were evaluated on Intel Xeon E5-2667 v3 8C processors with 3.2GHz and will depend on implementation. Markers are slightly offset on the x-axis for better visibility.

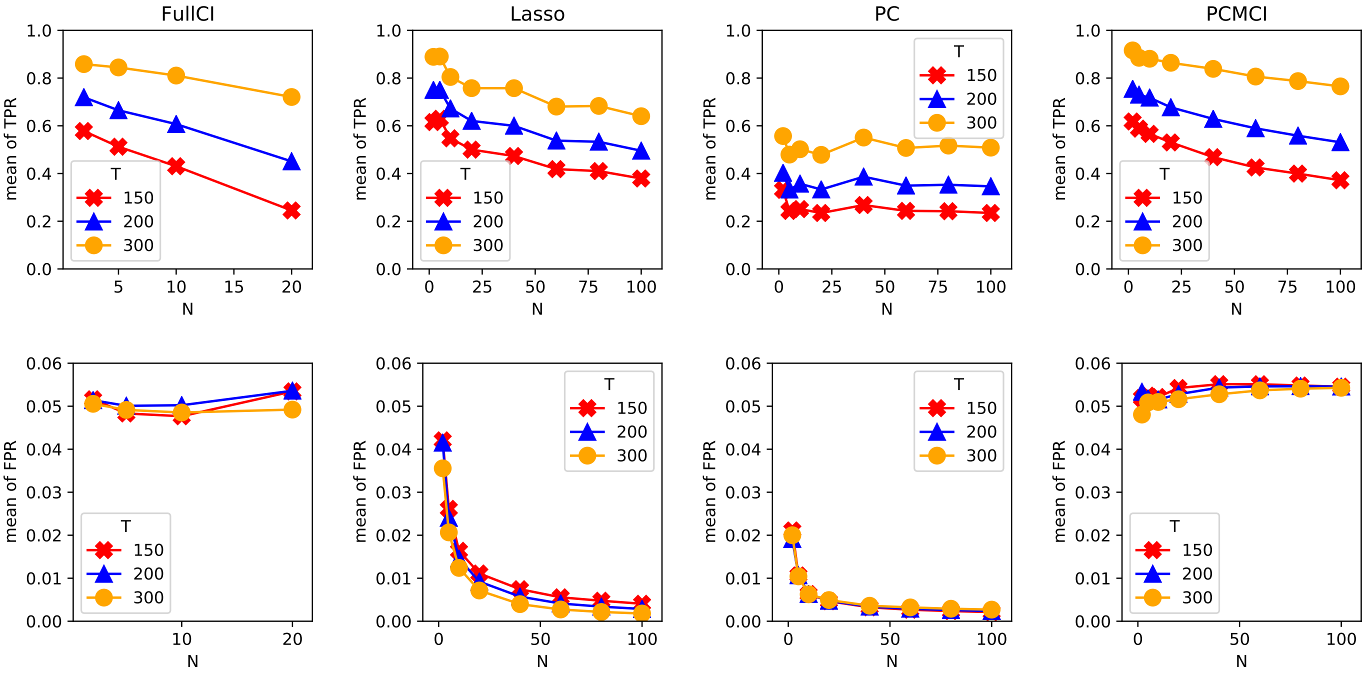

Fig. S17. **ANCOVA interaction plots.** The detailed setup is listed in Supplementary Tab. S2. The top row depicts the average true positives and the bottom row the average false positives for different numbers of variables N (x-axis) and different sample sizes T (colored lines) of the different methods. In the previous figures we show not only averages, but boxplots, which are omitted here to better depict the dependencies for different time series lengths T. An interaction between N and T is present if the difference between, for example, the detection power for $T = 300$ and $T = 150$ changes for different N, here present for FullCI and PCMCI, but not for Lasso and PC.

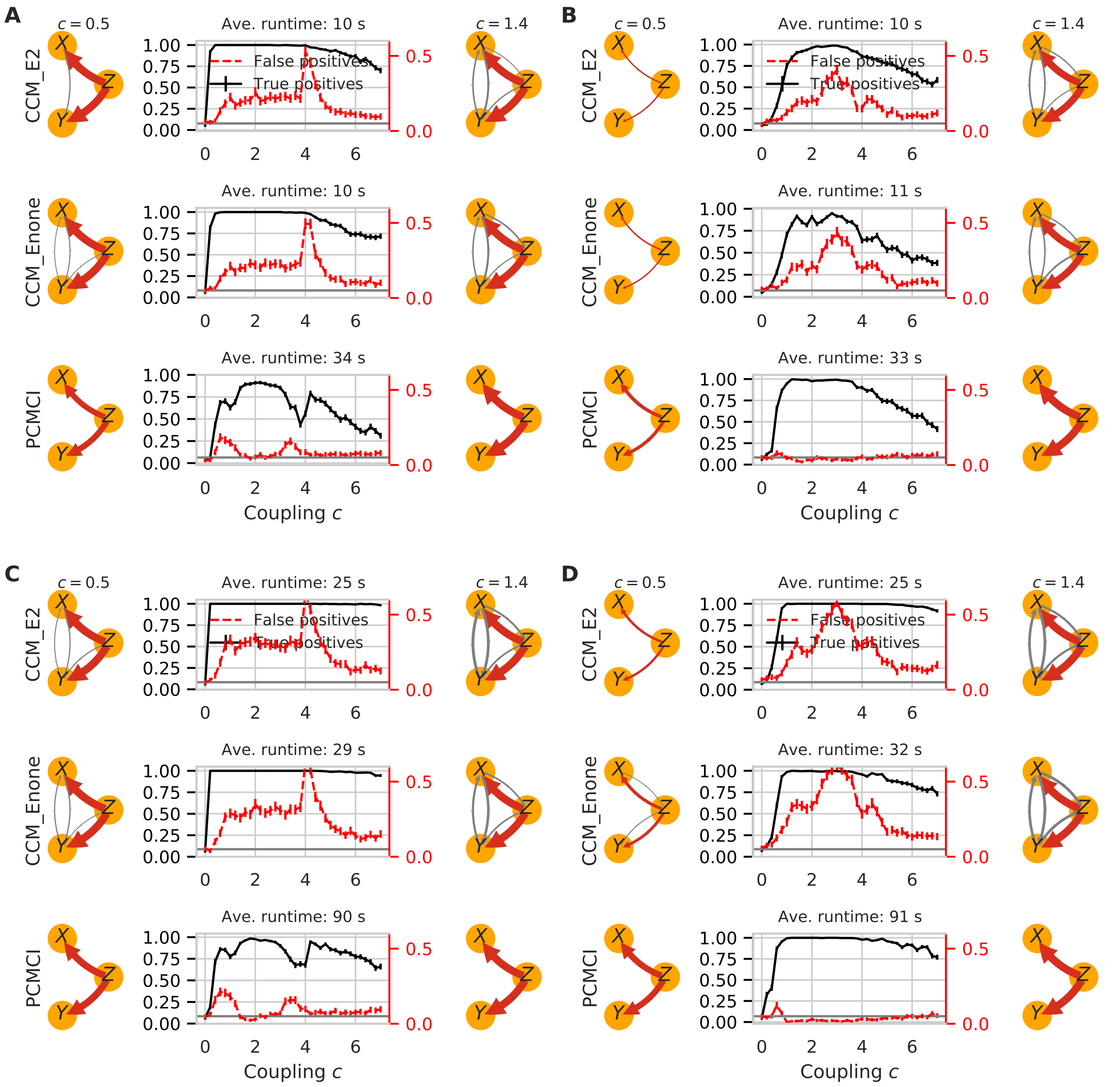

Fig. S18. **Comparison of PCMCI and CCM on logistics maps.**
Comparison of PCMCI in the CMI implementation with the nonlinear state-space method convergent cross-mapping (CCM) on a system of three coupled chaotic logistic maps. See Supplementary Sects. S6,S2.4 for a description of the system and CCM. CCM was estimated with fixed embedding dimension (CCM_E2) and with optimized embedding dimension (CCM_Enone). In the left and right graphs the width of arrows denotes the detection rate, grey edges depict false links (only rates > 0.08 shown). The center panels depict average true (black) and false positive (red) rates for different levels of common driver coupling strength c estimated from 200 realizations. The grey horizontal line depicts the 5% significance level which is the expected level. Errorbars denote standard errors. The panels show the results for (**A**) $T = 150$ and no dynamical noise, (**B**) $T = 150$ and $\sigma = 0.2$ dynamical noise, (**C**) $T = 300$ and no dynamical noise, (**D**) $T = 300$ and $\sigma = 0.2$ dynamical noise.
